# Supramolecular catalysis by recognition-encoded oligomers: discovery of a synthetic imine polymerase†

**DOI:** 10.1039/d0sc02234a

**Published:** 2020-07-06

**Authors:** Luca Gabrielli, Christopher A. Hunter

**Affiliations:** Department of Chemistry, University of Cambridge Lensfield Road Cambridge CB2 1EW UK herchelsmith.orgchem@ch.cam.ac.uk; Department of Chemistry, University of Padova via F. Marzolo 1 Padova 35131 Italy

## Abstract

All key chemical transformations in biology are catalysed by linear oligomers. Catalytic properties could be programmed into synthetic oligomers in the same way as they are programmed into proteins, and an example of the discovery of emergent catalytic properties in a synthetic oligomer is reported. Dynamic combinatorial chemistry experiments designed to study the templating of a recognition-encoded oligomer by the complementary sequence have uncovered an unexpected imine polymerase activity. Libraries of equilibrating imines were formed by coupling diamine linkers with monomer building blocks composed of dialdehydes functionalised with either a trifluoromethyl phenol (**D**) or phosphine oxide (**A**) H-bond recognition unit. However, addition of the **AAA** trimer to a mixture of the phenol dialdehyde and the diamine linker did not template the formation of the **DDD** oligo-imine. Instead, **AAA** was found to be a catalyst, leading to rapid formation of long oligomers of **D**. **AAA** catalysed a number of different imine formation reactions, but a complementary phenol recognition group on the aldehyde reaction partner is an essential requirement. Competitive inhibition by an unreactive phenol confirmed the role of H-bonding in substrate recognition. **AAA** accelerates the rate of imine formation in toluene by a factor of 20. The kinetic parameters for this enzyme-like catalysis are estimated as 1 × 10^−3^ s^−1^ for *k*_cat_ and the dissociation constant for substrate binding is 300 μM. The corresponding **DDD** trimer was found to catalyse oligomerisation the phosphine oxide dialdehyde with the diamine linker, suggesting an important role for the backbone in catalysis. This unexpected imine polymerase activity in a duplex-forming synthetic oligomer suggests that there are many interesting processes to be discovered in the chemistry of synthetic recognition-encoded oligomers that will parallel those found in natural biopolymers.

## Abstract 2

Enzyme-like catalytic properties have been found in synthetic recognition-encoded oligomers.

## Graphical abstract

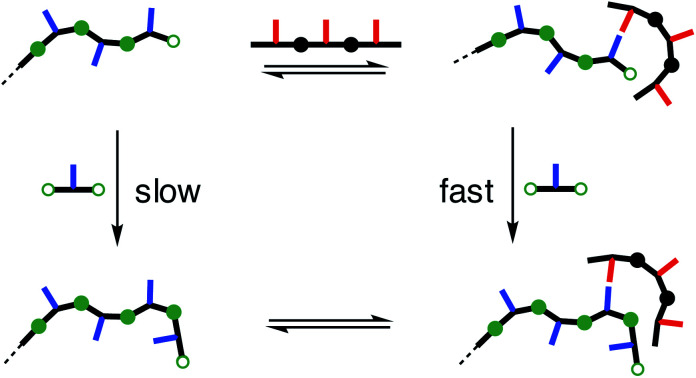

## Introduction

Recognition-encoded oligomers are the basis for the key chemical processes that lead to living systems: molecular replication, self-assembly, molecular recognition and supramolecular catalysis.^1^ The ability to process molecular information encoded as a sequence of monomer building blocks through replication, transcription and translation is currently unique to nucleic acids. Iterative cycles of selection and replication have been used in molecular evolution experiments to select functional nucleic acid sequences with recognition or catalytic properties from large pools of unbiased sequences.^2,3^ Synthetic recognition-encoded oligomers have the potential to display similar properties, which would open the way for directed evolution of function in synthetic polymers. The first steps in this direction have been taken with the development of oligomers that form duplexes in a sequence-selective manner, and a range of such systems have been reported.^4–9^

**Fig. 1:**
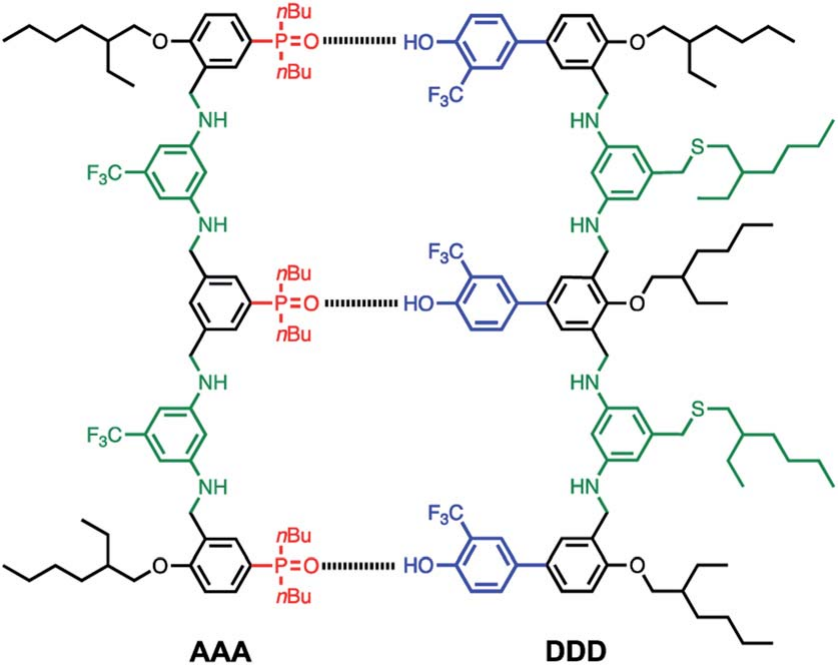
Chemical structure of the **AAA·DDD** duplex. The complementary recognition modules are phosphine oxide H-bond acceptors (red) and 2-trifluoromethyl phenol H-bond donors. Synthesis is based on reductive amination of bisaniline linkers (green) with dialdehydes monomer building blocks. The backbone modules (black) provide a geometry compatible with duplex formation. The 2-ethylhexyl groups ensure solubility in non-polar solvents.

We have recently reported a modular approach for the design of duplex forming molecules.^6^ The basic architecture is illustrated in Fig. 1.

There are four key elements to the design: the *recognition* modules that lead to duplex assembly are H-bond acceptors (red) and H-bond donors (blue) displayed as side chains; the *synthesis* of oligomers is based on reductive amination to couple bisaniline linkers (green) with dialdehyde monomer building blocks; the *backbone* module (black) was chosen to ensure that the oligomer has geometrical properties that are compatible with duplex formation; and the *solubilising groups* ensure solubility in the non-polar solvents required for high affinity H-bonding interactions.

The homo-oligomers illustrated in Fig. 1 form duplexes in toluene and in chloroform solution.^6*j*^ The association constant for the intermolecular H-bond between the phenol and phosphine oxide units used for base-pairing is 3 × 10^3^ M^−1^ in toluene, which leads to high stability, high fidelity duplex formation between complementary oligomers. There is a uniform increase in duplex stability with oligomer length indicating that this supramolecular framework is compatible with cooperative formation of H-bonds along an extended duplex. The duplex architecture is the basis for biological copying of nucleic acid sequence information through template-directed synthesis. Non-enzymatic DNA template-directed synthesis of non-natural sequence-defined oligomers has been performed using amide coupling,^10^ and reductive amination,^11,12^ with further extension to the assembly of sequence defined polymers.^13^

**Fig. 2:**
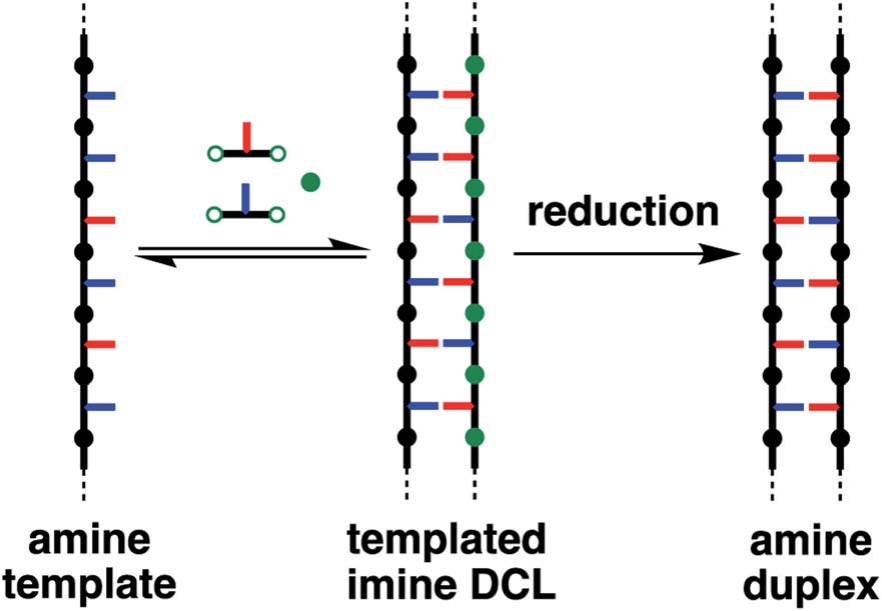
Non-covalent template-directed synthesis of recognition-encoded oligomers. The template is an amine (green) oligomer bearing recognition units (blue and red). Dynamic imine libraries can be built from dialdehydes and diamines equipped with recognition units. The template shifts the equilibrium, favouring the formation of the complementary sequence, which can be trapped as an amine oligomer.

The oligomers shown in Fig. 1 should be able to template their own synthesis in the same way. An attractive feature of the reductive amination chemistry used to synthesise these oligomers is that the coupling chemistry can be carried out sequentially in two steps: imine formation followed by reduction. Imine formation from an aniline and an aldehyde can be carried out under reversible conditions. Thus mixing the bisaniline linker with the two dialdehyde building blocks used to assemble the oligomers in Fig. 1 will lead to a dynamic combinatorial library (DCL) of equilibrating recognition-encoded oligo-imines.^14^ Addition of a recognition-encoded amine template, such as **AAA** shown in Fig. 1, should cause a shift in the DCL equilibrium in favour of the complementary **DDD** oligomer, which would form the most stable duplex with the template (Fig. 2).^15^ The DCL could then be trapped by reduction to obtain the complementary copy of the template as an oligo-amine. Here we describe experiments designed to explore non-covalent template directed synthesis of recognition-encoded oligomers based on the duplex shown in Fig. 1. However, rather than acting as a template for selective formation of **DDD**, it turns out that **AAA** is a catalyst for selective elongation of homo-oligomers of H-bond donor monomers.^16^

## Results and discussion

**Fig. 3:**
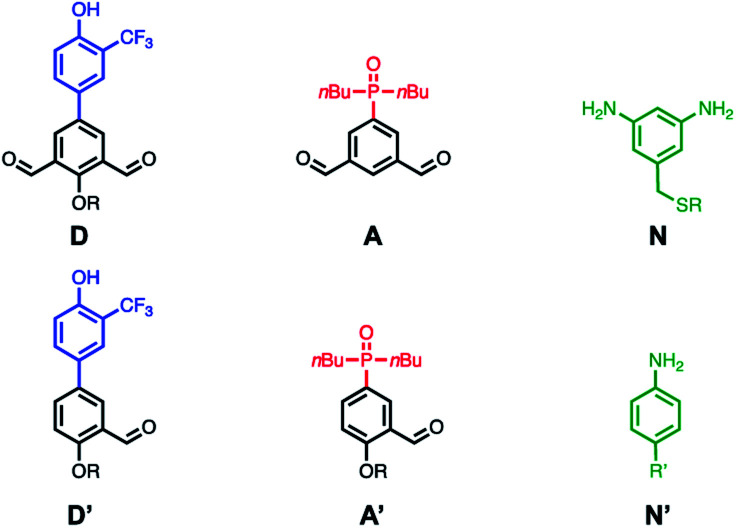
Structures of the acceptor and donor dialdehyde (**D**, **A**) and dianiline (**N**) monomers used for building libraries of imine oligomers. Structures of monoaldehyde (**D′**, **A′**) and monoaniline (**N′**) monomers used as chain end capping units (R = 2-ethylhexyl, R′ = pentyl).

**Fig. 4:**
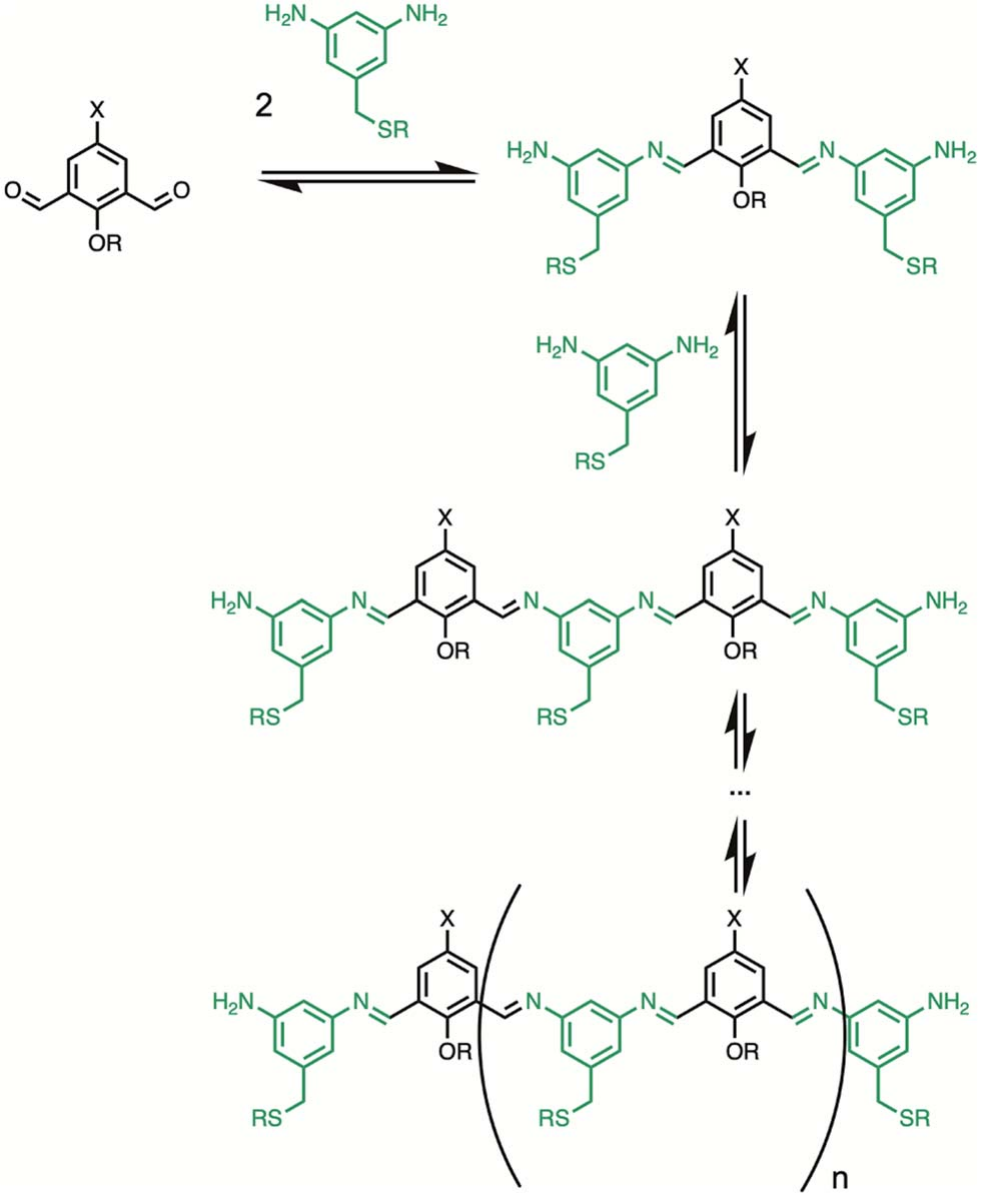
Equilibria involved in the formation of a library of imine oligomers. If no cyclic oligomers are formed, the degree of oligomerization can be controlled with the amount of dianiline. X is a recognition module, and R is a solubilizing group.

The building blocks used for oligomer synthesis are illustrated in Fig. 3. **D** and **D′** are dialdehyde and mono-aldehyde recognition units equipped with 2-trifluoromethylphenol H-bond donor groups. **A** and **A′** are the complementary dialdehyde and mono-aldehyde H-bond acceptor recognition units equipped with phosphine oxide groups. **N** is the bisaniline linker used for the dynamic covalent assembly of oligomers, as illustrated in Fig. 4, and **N′** is a monofunctional aniline chain end capping group. Mixing the linker **N** with either or both of the dialdehydes (**D**, **A**) should lead to formation of a library of interconverting oligomers (Fig. 4).

**Fig. 5:**
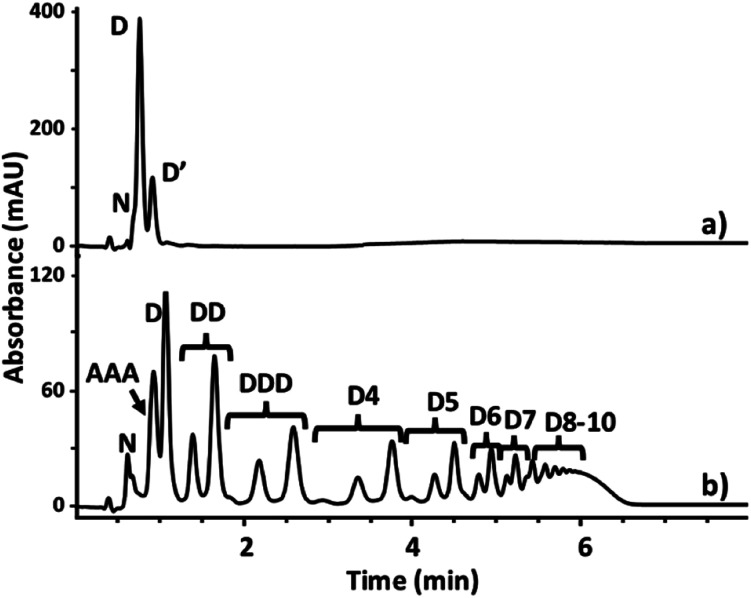
HPLC traces of a toluene-*d*8 solution of donor **D** (10 mM) 5 minutes after the addition of dianiline **N** (16 mM) in the presence of phosphine oxides. (a) 10 mM *n*Bu_3_PO. The peak corresponding to the monomeric mono-imine mono-aldehyde is labelled **D′**. (b) 3.3 mM **AAA**. The imine oligomers are labelled **D**, **DD**, **DDD**, **D4**, **D5***etc.* For each oligomer, three peaks were observed, corresponding to dialdehyde (traces), mono-imine mono-aldehyde and diimine terminal groups. HPLC method: coagent phenyl hydride 2.o 3 cm × 3 mm column, flow rate 0.4 ml min^−1^, injection volume 0.1 μL, 45 °C; eluent A 60% NH_4_OAc 10 mM, pH 5.70, 40% THF; eluent B 85% THF, 10% IPA, 5% A; 45% B for 1 min, then increased to 75% over 3.5 min, and then increased to 80% over 2 min. The UV/vis absorbance was recorded at 292–308 nm.

The effective molarity for the intramolecular interactions leading to duplex formation was previously measured as 30 mM in toluene.^6*j*^ Working at a monomer concentration of 10 mM should therefore favour oligomerisation in the presence of a template. At this concentration, more than 80% of the monomers will be bound to the template, and the **AAA·DDD** duplex will be more than 99% bound.^17^ Even though the aniline linkers were equipped with solubilising groups, mixing **D** and **N** in toluene in the absence of a template lead to precipitation. The presence of one equivalent of tri-*n*-butyl phosphine oxide was sufficient to keep everything in solution, and so the untemplated control experiment was carried out under these conditions. However, mixing **D** and **N** in toluene in the presence of **AAA** lead to rapid precipitation. These initial observations already indicate that **AAA** has a significant effect on the reaction. Based on the assumption that the precipitate was due to formation of polymers, the reaction was tested using excess **N** in order to limit the chain length. More than 1.6 equivalents of **N** was sufficient to keep everything in solution in the presence of **AAA**. The results are shown in Fig. 5.

Imine formation was monitored using HPLC-MS. Imine-based DCLs are typically fixed for HPLC analysis by reduction to the corresponding amines.^18^ However, the reagents required could interfere with H-bonding interactions with the **AAA** template and alter the oligomer population in this experiment, so direct chromatographic analysis of the imine library is preferable. It was possible to minimise imine hydrolysis on the HPLC column by using a mixture of THF and ammonium acetate buffer (pH 5.7) as the eluent, allowing direct analysis of the imine DCL. The HPLC results shown in Fig. 5 show peaks due to oligomers with both aldehyde and imine end groups. However, these HPLC traces were recorded after 5 minutes, and when the HPLC experiment was repeated after an hour, no oligomers with aldehyde end groups were observed, confirming that there is no imine hydrolysis on the HPLC column (see Fig. S79†). The HPLC peaks were assigned using the corresponding mass spectra. The HPLC results in Fig. 5 show that there is very little imine formation after 5 minutes in the absence of template. Addition of the **AAA** leads to a much faster reaction and the formation a complex mixture of different length linear oligomers. However, there is no obvious template effect, and the yield of **DDD** does not appear to be enhanced relative to the other oligomers. When the control reaction without template was left to equilibrate for 13 hours, the resulting HPLC trace was similar to that obtained in the presence of **AAA** (see Fig. S80†). In neither case were any cyclic oligomers detected. Thus **AAA** appears to be a catalyst for imine formation rather than a template.

**Fig. 6:**
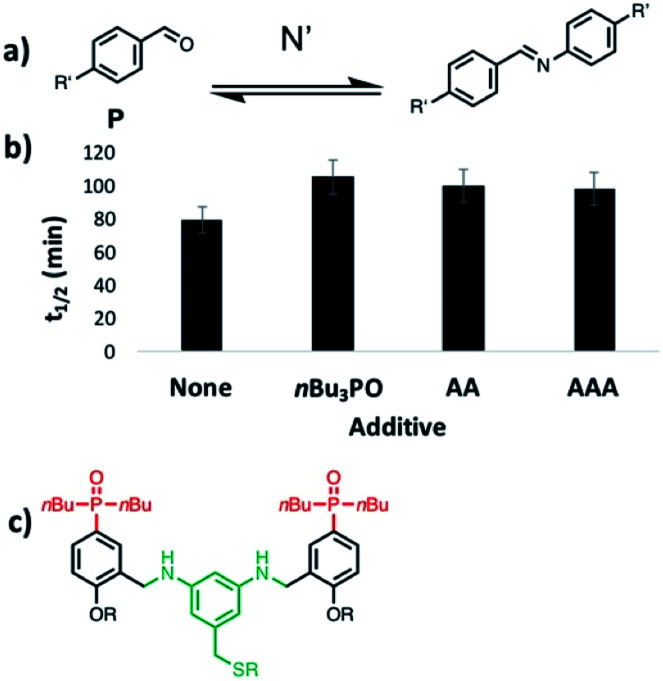
Effects of phosphine oxide oligomers on the rate of imine formation for substrates with no recognition sites. (a) Reaction of 4-pentylbenzaldehyde with 4-pentylaniline (R′ = *n*-pentyl). (b) Half-life of the aldehyde starting material measured by integration of 500 MHz ^1^H NMR signals as a function of time. Reaction conditions: 20 mM aldehyde, 40 mM aniline and 10 mM phosphine oxide in toluene-*d*8 (*i.e.* 10 mM *n*Bu_3_PO, 5 mM **AA**, or 3.3 mM **AAA**). (c) Chemical structure of **AA**.

In order to assess whether H-bonding interactions between the phenol groups on the monomer units and the phosphine oxide recognition groups on **AAA** play an important role the catalytic activity, we investigated the reaction between an aniline and an aldehyde that have no H-bonding sites (**N′** and **P** in Fig. 6a). ^1^H-NMR spectroscopy was used to monitor the rate of appearance of the imine signal at 9 ppm due to the product in the presence of various additives: *n*Bu_3_PO, **AA** or **AAA**. In comparison with the control experiment, none of the phosphine oxide additives have any significant effect on the reaction rate. This observation suggests that H-bonding interactions between the **D** monomer units and **AAA** play an important role in the catalysis of imine formation.

**Fig. 7:**
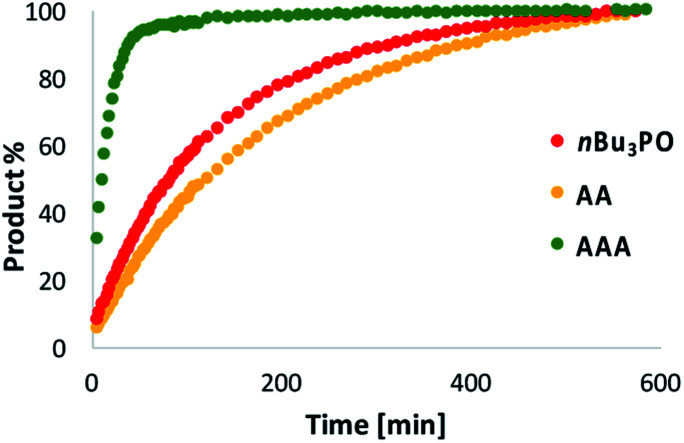
Kinetics of imine formation measured by integration of all imine signals in the 500 MHz ^1^H NMR spectra of a mixture of **N** (20 mM) and **D** (10 mM) in toluene-*d*8 in the presence of 10 mM *n*Bu_3_PO (red data), 5 mM **AA** (yellow data) or 3.3 mM **AAA** (green data).

Next we investigated whether a trimer is required for catalysis. Fig. 7 compares the rate of reaction of **D** with **N** in the presence of a monomeric phosphine oxide (*n*Bu_3_PO), **AA** or **AAA**. Although this reaction generates many different oligomers (see Fig. 5), it was possible to measure the total amount of imine formed by integrating all of the signals between 8.9 and 9.1 ppm in the ^1^H NMR spectrum. In the presence of **AA**, the reaction proceeds at a similar rate to the control experiment with *n*Bu_3_PO. In the presence of **AAA**, the rate of reaction is about one order of magnitude higher, consistent with the HPLC results in Fig. 5. This observation indicates that the trimer is the minimum length oligomer required for catalysis.

**Fig. 8:**
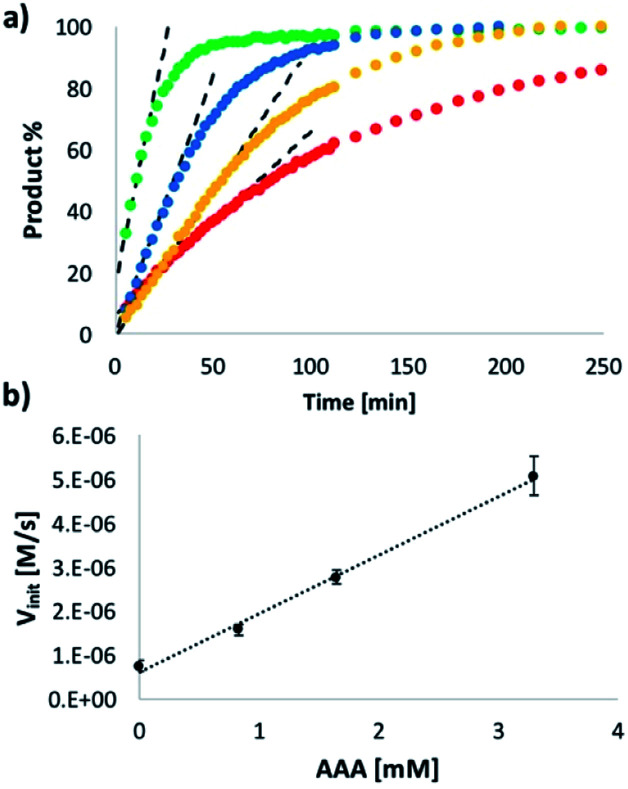
(a) Kinetics of imine formation measured by integration of all imine signals in the 500 MHz ^1^H NMR spectra of a mixture of **N** (20 mM) and **D** (10 mM) in toluene-*d*8 in the presence of different amounts of **AAA**: 0 (red), 0.83 (yellow), 1.65 (blue) or 3.3 (green) mM. The dotted lines show the linear region where the initial rate (*V*_init_) was measured. (b) Relationship between the initial rate (*V*_init_) and the concentration of **AAA**. The straight line of best fit is shown.

The order of reaction with respect to **AAA** was determined by measuring the rate as a function of catalyst concentration using ^1^H-NMR spectroscopy. Fig. 8a shows that the rate of imine formation increases with increasing amounts of **AAA**. The initial rates *V*_init_ are plotted as a function of **AAA** concentration in Fig. 8b, and the linear relationship shows that the reaction is first order in **AAA**, confirming that this oligomer is the active species. These data also show that there is efficient turnover of the catalyst. In all of the reactions in Fig. 8b, **AAA** is present in substoichiometric amounts (8–33 mol%), but it converts 100% of the substrate into product.

**Fig. 9:**
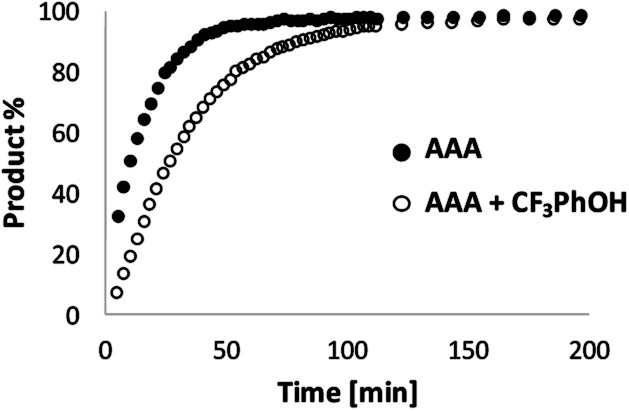
Kinetics of imine formation measured by integration of all imine signals in the 500 MHz ^1^H NMR spectra of a mixture of **N** (20 mM) and **D** (10 mM) in toluene-*d*8 in the presence of 3.3 mM **AAA** in the absence (filled circles) or presence of 10 mM 2-trifluoromethyl phenol (open circles).

The oligomerisation of **N** and **D** is a multi-step process, and the solubility of the oligomeric products limits the substrate concentration range that can be studied, so we have not been able to demonstrate saturation kinetics with this system. Therefore in order to confirm the role played by substrate recognition in the catalytic activity of **AAA**, 2-trifluoromethyl phenol was added as a competitive inhibitor. The results of ^1^H-NMR kinetic experiments are shown in Fig. 9. The rate of reaction between **D** and **N** is significantly slower in the presence of the inhibitor: *V*_init_ drops from 5.2 to 3.6 μM s^−1^ in the presence of one equivalent of 2-trifluoromethyl phenol. Note that the addition of 2-trifluoromethyl phenol was observed to cause a small increase in the rate of imine formation in the reaction of **P** with **N′** (see Fig. S52†), which suggests that the observed inhibition is an underestimate of the true effect of competitive substrate binding. These results confirm that H-bond interactions between **AAA** and **D** play an important role in the catalytic process.

The data in Fig. 8 can therefore be used to estimate the kinetic parameters that describe the enzyme-like properties of **AAA**. The dissociation constant *K*_d_ for the H-bonding interaction between the dialdehyde substrate **D** and the phosphine oxide groups on **AAA** was previously measured as 300 μM in toluene. The reactions in Fig. 8b were all carried out at a substrate concentration of 10 mM and with excess **N**, so if we assume that the value of *K*_M_ is similar to the value of *K*_d_ reported above, the catalyst **AAA** must be saturated under the reaction conditions. Thus the slope of Fig. 8b (1.3 × 10^−3^ s^−1^) is equivalent to the value of *k*_cat_, and the value of the corresponding pseudo first order rate constant for the uncatalyzed reaction *k*_uncat_ is the ratio of the intercept of Fig. 8b and the substrate concentration (6.2 × 10^−5^ s^−1^). Thus we can estimate the rate acceleration for the catalysed reaction *k*_cat_/*k*_uncat_ as approximately 20.

**Fig. 10:**
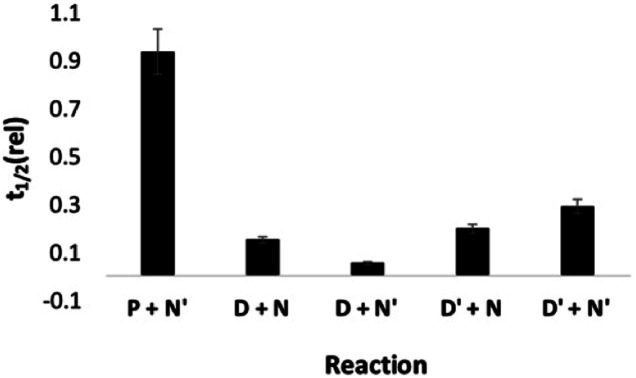
Half-life for reactions in the presence of **AAA** (3.3 mM) relative to the corresponding half-life in the presence of *n*Bu_3_PO (10 mM) (*t*_1/2_ (rel)). Half-lives were measured by integration of the 500 MHz ^1^H NMR signal due to the aldehyde as a function of time for mixtures of 20 mM total aldehyde (20 mM **D′**, 10 mM **D**, or 20 mM **P**) and 40 mM total aniline (40 mM **N′**, or 20 mM **N**) in toluene-*d*8.

Next we investigated the effect of **AAA** on systems that do not form polymers by using the mono-aldehyde **D′** in place of **D** and the mono-aniline **N′** in place of **N** (see Fig. 3). The results are shown in Fig. 10. For all four combinations of substrate, the catalytic effect of **AAA** on imine formation is similar. This result shows that formation of a H-bond with one of the coupling partners is sufficient for catalysis. The aldehyde substrate must have a H-bonding recognition site that allows binding to the catalyst, but reaction with any aniline will then be accelerated. This observation explains why no length selectivity is observed in the product distribution in Fig. 5: **AAA** simply acts as an elongation catalyst, an imine polymerase.

**Fig. 11:**
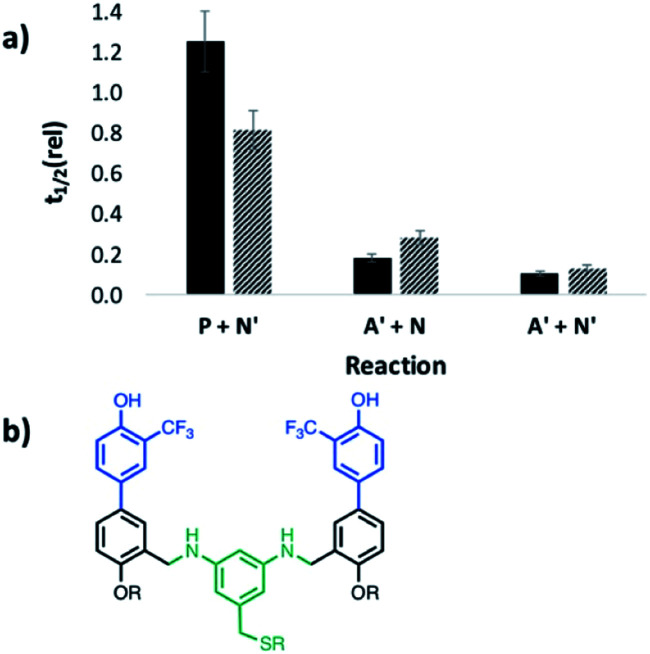
a) Half-life for reactions in the presence of **DD** (5 mM, shaded data) or **DDD** (3.3 mM, black data) relative to the corresponding half-life in the presence of 2-trifluoromethyl phenol (10 mM) (*t*_1/2_ (rel)). Half-lives were measured by integration of the 500 MHz ^1^H NMR signal due to the aldehyde as a function of time for mixtures of 20 mM aldehyde (**P** or **A′**) and 40 mM total aniline (40 mM **N′**, or 20 mM **N**) in toluene-*d*8. (b) Chemical structure of **DD**.

To evaluate the possible role of the phosphine oxide groups in catalysis, this experiment was repeated but with the recognition units swapped between the substrate and catalyst. Addition of **DDD** increased the rate of reaction of the dialdehyde phosphine oxide monomer **A** with the bisaniline linker **N**, but the imine oligomers formed were not sufficiently soluble in toluene to measure reaction rates using ^1^H-NMR spectroscopy (see Fig. S56†). It was possible to measure the rate of imine formation with the corresponding mono-aldehyde **A′**, and the results are shown in Fig. 11. In this case, both the dimer **DD** and the trimer **DDD** are effective catalysts for imine formation with either the monofunctional aniline **N′** or with the bisaniline **N**. Both **DD** and **DDD** increase the rate by an order of magnitude compared to the experiment with the corresponding monomeric phenol, 2-trifluoromethyl phenol. No catalysis was observed for reactions with an aldehyde lacking a recognition site, **P**, which implies that phenol–phosphine oxide H-bonding is important for catalysis by the donor oligomers. It is possible that the phenol oligomers could catalyse imine formation by a different mechanism from the phosphine oxide oligomers, but the similarities between Fig. 10 and 11 suggests that it is the backbone rather than the recognition units that are responsible for the catalysis of imine formation by **AAA**. The phenol recognition module is significantly longer than the phosphine oxide recognition module, and the difference in the geometric relationship between the substrate binding site, the position of the reactive aldehyde, and the backbone might explain why **DD** is a catalyst and **AA** is not.

**Fig. 12:**
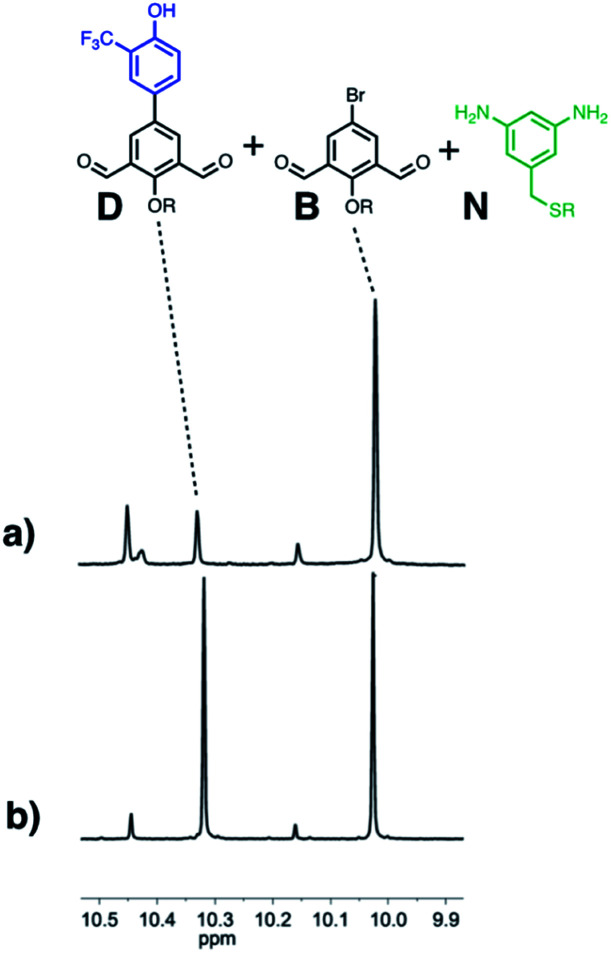
Partial 500 MHz ^1^H-NMR spectra of a mixture of **D** (10 mM) and **B** (10 mM) in toluene-*d*8 solution 5 minutes after the addition of **N** (20 mM) in the presence of (a) **AAA** (3.3 mM) or (b) *n*Bu_3_PO (10 mM). The signals at 10.4–10.5 ppm are due to the aldehyde protons at the end of **D** oligomers; the signal at 10.1–10.2 ppm is due to the aldehyde proton of the mono-imine of **B**.

Finally, the ability of **AAA** to selectively catalyse oligomerisation of **D** in the presence of a dialdehyde lacking any H-bond recognition sites (**B**) was investigated. Fig. 12 shows ^1^H NMR spectra of mixtures of **D**, **B** and **N** 5 minutes after addition of either *n*Bu_3_PO or **AAA**. In the presence of the monomeric phosphine oxide, the major species observed were the two dialdehydes **D** and **B**, and only traces of imine were formed. In the presence of **AAA**, **B** did not react, but **D** was rapidly converted into imine oligomers. When the reaction was monitored for longer time periods in the presence of the monomeric phosphine oxide, the amount of imine formed was similar for the two dialdehydes **D** and **B** (see Fig. S69†), so the differences observed in the presence of **AAA** are not due a difference in the inherent reactivity of the two substrates. Thus H-bonding interactions between the **D** monomers and **AAA** leads to selective oligomerisation in the presence of other possible substrates.

## Conclusions

Oligomers equipped with complementary recognition groups as side chains form stable duplexes. In the system described here, the recognition groups are 2-trifluoromethyl phenol and phosphine oxide, which form strong H-bonds in toluene solution, and the backbone is an oligo-amine made from dialdehyde and bisaniline building blocks. Mixing the dialdehyde and bisaniline components in toluene leads to a dynamic combinatorial library (DCL) of different imine oligomers. The effects of adding recognition-encoded amine oligomers to this reaction mixture was investigated using NMR spectroscopy and HPLC to characterise the rate of reaction and the product distribution. Specifically, addition of the **AAA** amine oligomer to a mixture of the **D** dialdehyde and bisaniline linker did not template formation of the complementary **DDD** imine oligomer, and no change in product distribution was observed.

**Fig. 13:**
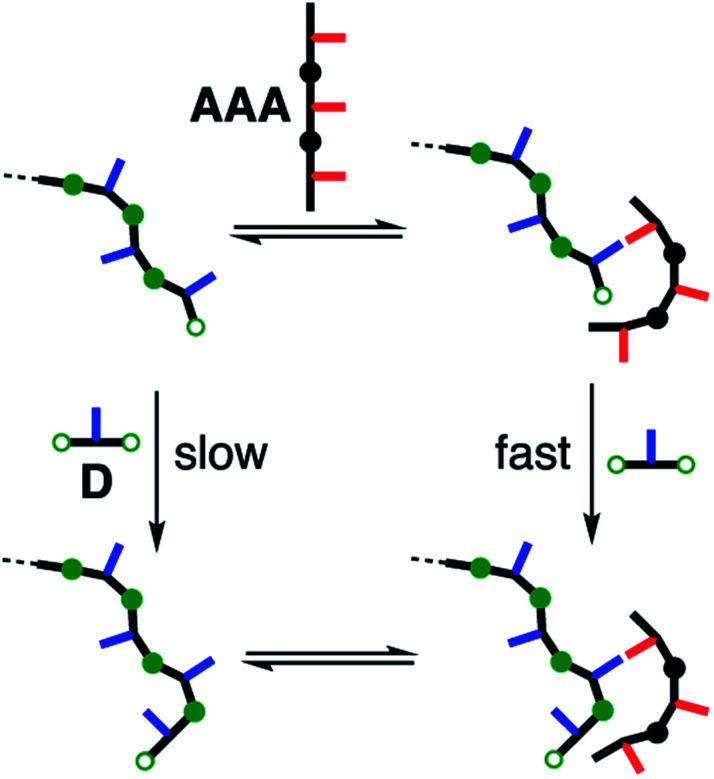
Elongation of an imine oligomer of **D** catalysed by **AAA**. H-bonding interactions between phenol and phosphine oxide recognition units are required for catalysis.

However, addition of **AAA** did increase the rate of imine oligomerisation by an order of magnitude (Fig. 13). **AAA** was found to catalyse imine formation reactions between different substrates, but the minimal requirement for activity is that the aldehyde must have a phenol recognition group to allow the substrate to bind to the catalyst. Addition of a phenol as a competitive inhibitor was found to reduce the rate of reaction, confirming the role of substrate recognition in the observed activity. **AAA** accelerates the rate of imine formation in toluene by a factor of 20. The kinetic parameters for this enzyme-like catalysis are estimated as 1 × 10^−3^ s^−1^ for *k*_cat_ and the dissociation constant for substrate binding is 300 μM. Neither a simple phosphine oxide nor the shorter **AA** oligomer showed any catalytic activity. The corresponding phenol oligomers **DD** and **DDD** were found to catalyse imine formation with an aldehyde bearing a complementary phosphine oxide recognition group, which suggests a key role for the backbone in catalysis. The secondary amines in the backbone are the most likely functional groups to play a role in catalysis. One possible mechanism is intramolecular reaction of a backbone amine of **AAA** with an aldehyde on the end of a growing **D** oligomer, which is bound to **AAA**. The resulting iminium ion intermediate would provide a pathway for nucleophilic covalent catalysis of imine formation. Oligomerisation of the phenol monomer **D** is first order in **AAA**, which is also active in catalytic amounts. This unexpected imine polymerase activity in a synthetic oligomer suggests that there are many interesting processes to be discovered in the chemistry of synthetic recognition-encoded oligomers that will parallel those found in natural biopolymers.^19^

## Conflicts of interest

There are no conflicts to declare.

## Supplementary Material

SC-011-D0SC02234A-s001
