# Supplementary material for: Supramolecular catalysis by recognition-encoded oligomers: discovery of a synthetic imine polymerase
Source: Chem Sci. 2020 Jul 6;11(28):7408–14. doi: 10.1039/d0sc02234a (PMC8159439; doi:10.1039/d0sc02234a)
Supplement: SC-011-D0SC02234A-s001 [file SC-011-D0SC02234A-s001.pdf]

Electronic Supplementary Material (ESI) for Chemical Science.
This journal is © The Royal Society of Chemistry 2020

Supporting Information

Supramolecular catalysis by synthetic recognition-encoded oligomers: discovery of an imine polymerase.

Luca Gabrielli[a,b] and Christopher A. Hunter*[a]

a) Department of Chemistry, University of Cambridge, Lensfield Road, Cambridge CB2 1EW, UK
b) Department of Chemistry, University of Padova, via F. Marzolo 1, Padova 35131, Italy

Table of Contents

1. Experimental procedures S2

2. NMR spectroscopy studies S3

2.1. ^{1}H-NMR Kinetic experiments: **D**-**N** S4

2.2. ^{1}H-NMR Kinetic experiments: Varying the aniline concentration S9

2.3. ^{1}H-NMR Kinetic experiments: Varying the concentration of **AAA** S16

2.4. ^{1}H-NMR Kinetic experiments: **D**-**N** vs **AAA** - Competition experiment S18

2.5. ^{1}H-NMR Kinetic experiments: **D**-**N’** S19

2.6. ^{1}H-NMR Kinetic experiments: **D’**-**N** S21

2.7. ^{1}H-NMR Kinetic experiment: **D’**-**N** S24

2.8. ^{1}H-NMR Kinetic experiment: **P**-**N’** S26

2.9. ^{1}H-NMR Kinetic experiments: **A**-**N** S34

2.10 ^{1}H-NMR Kinetic experiments: **A’**-**N** S36

2.11 ^{1}H-NMR Kinetic experiments: **A’**-**N’** S39

2.12 ^{1}H-NMR Kinetic experiments: **AAA** selectivity S42

3. HPLC-MS studies S43

3.1. HPLC-MS experiments: peaks characterisation S44

3.2. HPLC-MS Kinetic experiments: **D**-**N** S45

4. References S52

1. Experimental Procedures

The monomers and the oligomers used in this work were synthesised following the synthetic procedure that we recently reported.[1] The reagents and materials used in the synthesis were bought from commercial sources, without prior purification. Thin layer chromatography was carried out using with silica gel 60F (Merck) on aluminium. Flash chromatography was carried out on an automated system (Combiflash Companion, Combiflash Rf+ or Combiflash Rf Lumen) using prepacked cartridges of silica (25µ or 50µ PuriFlash® Columns). All NMR spectroscopy was carried out on a Bruker AVI250, AVI400, DPX400, AVIII400 spectrometer using the residual solvent as the internal standard. All chemical shifts (δ) are quoted in ppm and coupling constants given in Hz. HPLC analysis were performed using a modular Agilent 1200 Series HPLC system composed of a HPLC high pressure binary pump, autosampler with injector programming capabilities, Peltier type column oven with 6 µL heat exchanger and a Diode Array Detector with a semi-micro flow cell (1.6uL, 6mm pathlength) to reduce peak dispersion when using short columns as in this case. The flow-path was connected using 0.12 mm ID stainless steel tubing to minimize peak dispersion. For the analysis of system ii, an HPLC method described in Section 3 was used.

2. NMR spectroscopy studies

All NMR kinetic experiments were performed using a Bruker 500 MHz AVIII HD Smart Probe spectrometers. Stock solutions of the selected monomers were freshly prepared in toluene-*d8* at a known concentration. A known volume of aldehyde monomer and the monomeric or oligomeric complementary units were added to an NMR tube. Finally, a known volume of the aniline solution was added to the NMR tube, which was quickly sealed and vigorously shaken, before starting the spectra acquisition.

2.1 ^{1}H-NMR Kinetic experiments: D-N

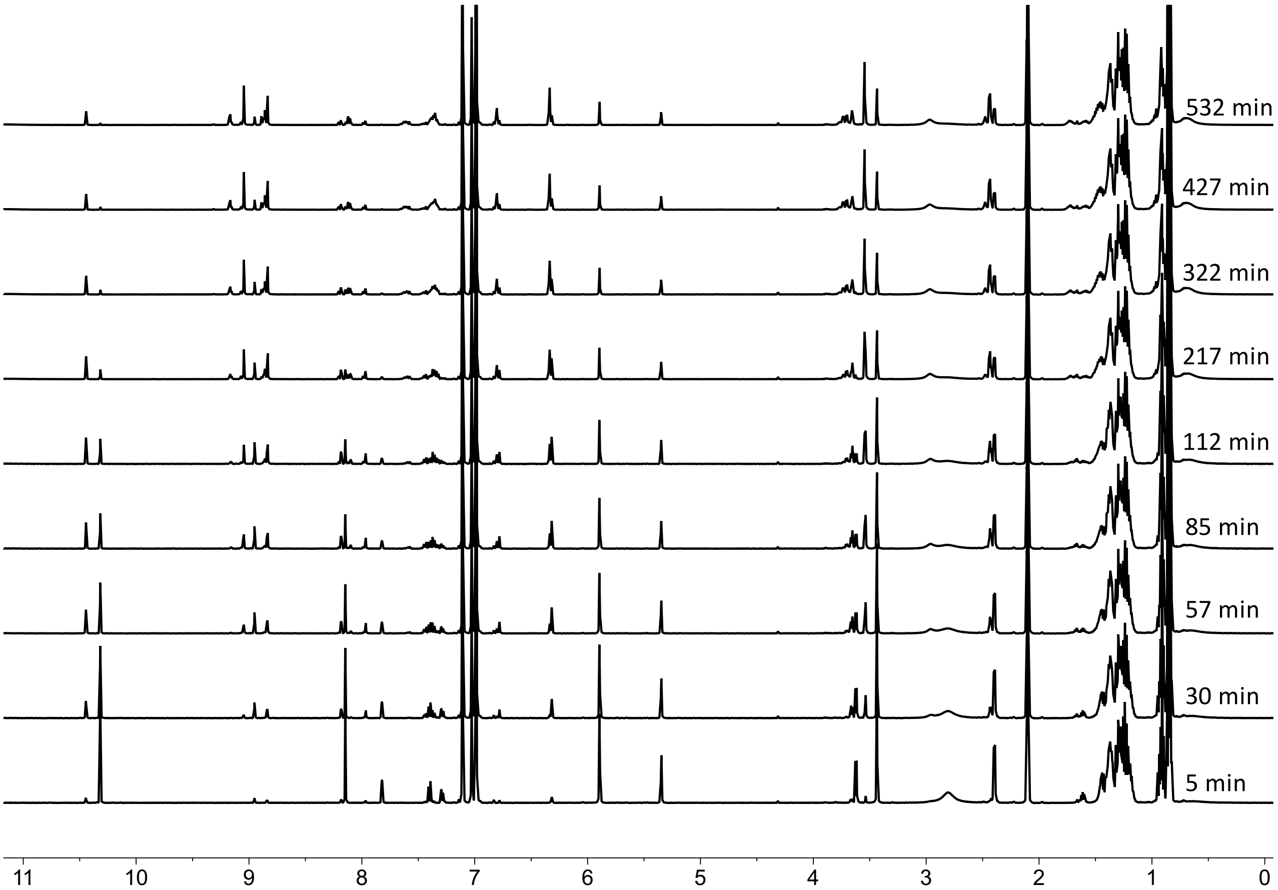

Figure S1. 500 MHz ^{1}H NMR spectra of a mixture of **N** (20 mM) and **D** (10 mM) in toluene-*d*8 in the presence of 10 mM ***n*Bu₃PO**. On the right side of the figure is reported the time (in minutes) after the addition of **N**.

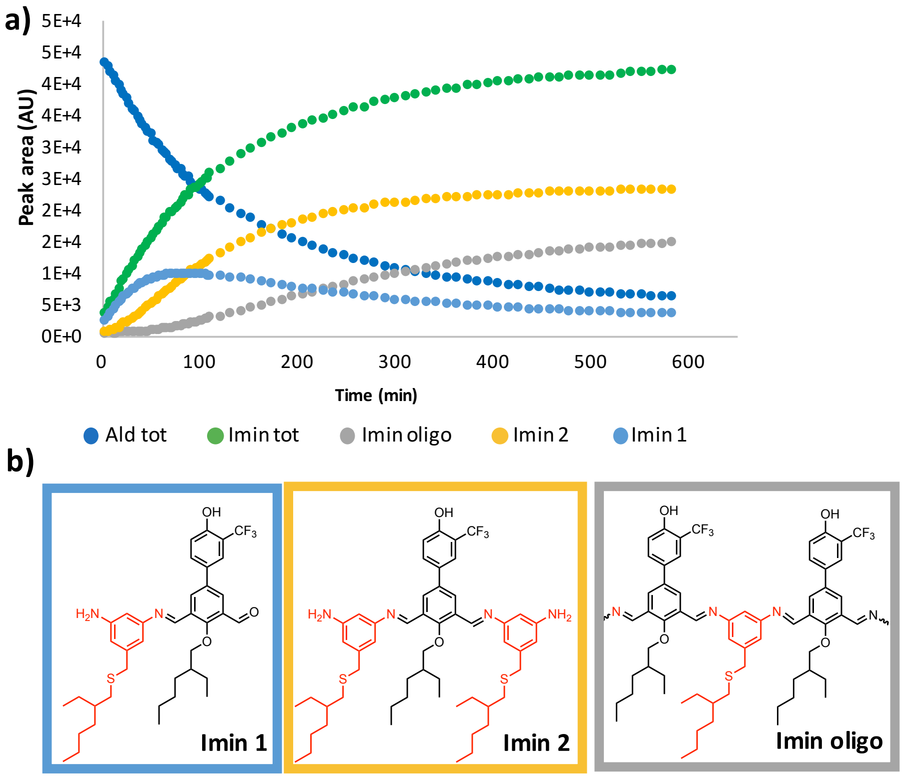

Figure S2. a) Kinetics of imine formation measured by integration of all imine and aldehyde signals in the 500 MHz ^{1}H NMR spectra (**D** 10 mM, **N** 20 mM, ***n*Bu₃PO** 10 mM). Total aldehyde signals (**Ald tot**, 10.51-10.26 ppm) and total imine (**Imin tot**, 9.25-8.92 ppm), together with monoimine (**Imin 1**, 8.97-8.93 ppm), monomeric bis imine (**Imin 2**, 9.10-9.00 ppm) and oligomeric imines (**Imin oliogo**, 9.22-9.12 ppm) are integrated and reported. **b)** Chemical structure of the formed imines.

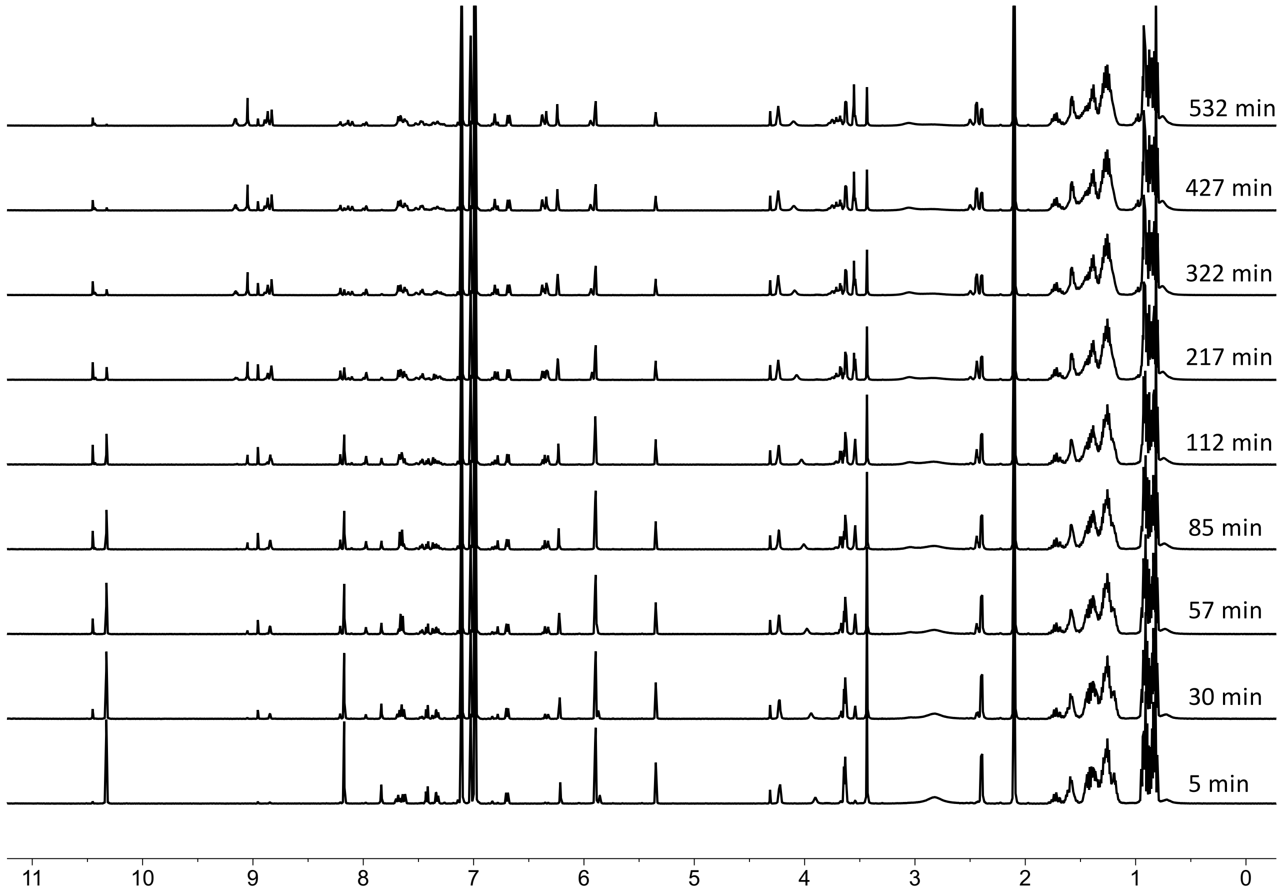

Figure S3. 500 MHz ^{1}H NMR spectra of a mixture of **N** (20 mM) and **D** (10 mM) in toluene-*d*8 in the presence of 5 mM **AA**. On the right side of the figure is reported the time (in minutes) after the addition of **N**.

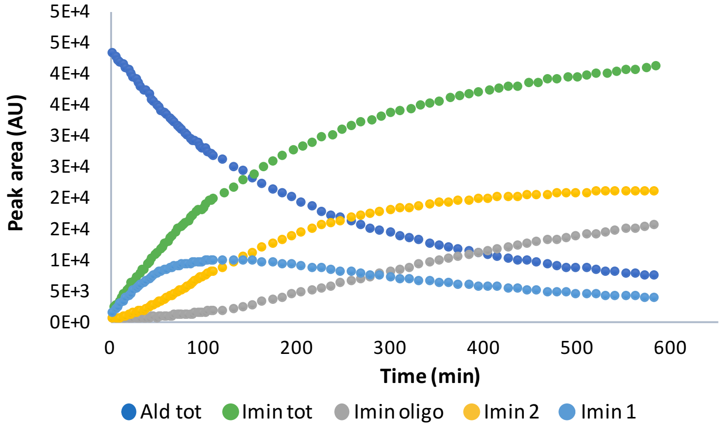

Figure S4. Kinetics of imine formation measured by integration of all imine and aldehyde signals in the 500 MHz ^{1}H NMR spectra (**D** 10 mM, **N** 20 mM, **AA** 5 mM). Total aldehyde signals (**Ald tot**, 10.51-10.26 ppm) and total imine (**Imin tot**, 9.25-8.92 ppm), together with monoimine (**Imin 1**, 8.97-8.93 ppm), monomeric bis imine (**Imin 2**, 9.10-9.00 ppm) and oligomeric imines (**Imin oliogo**, 9.22-9.12 ppm) are integrated and reported.

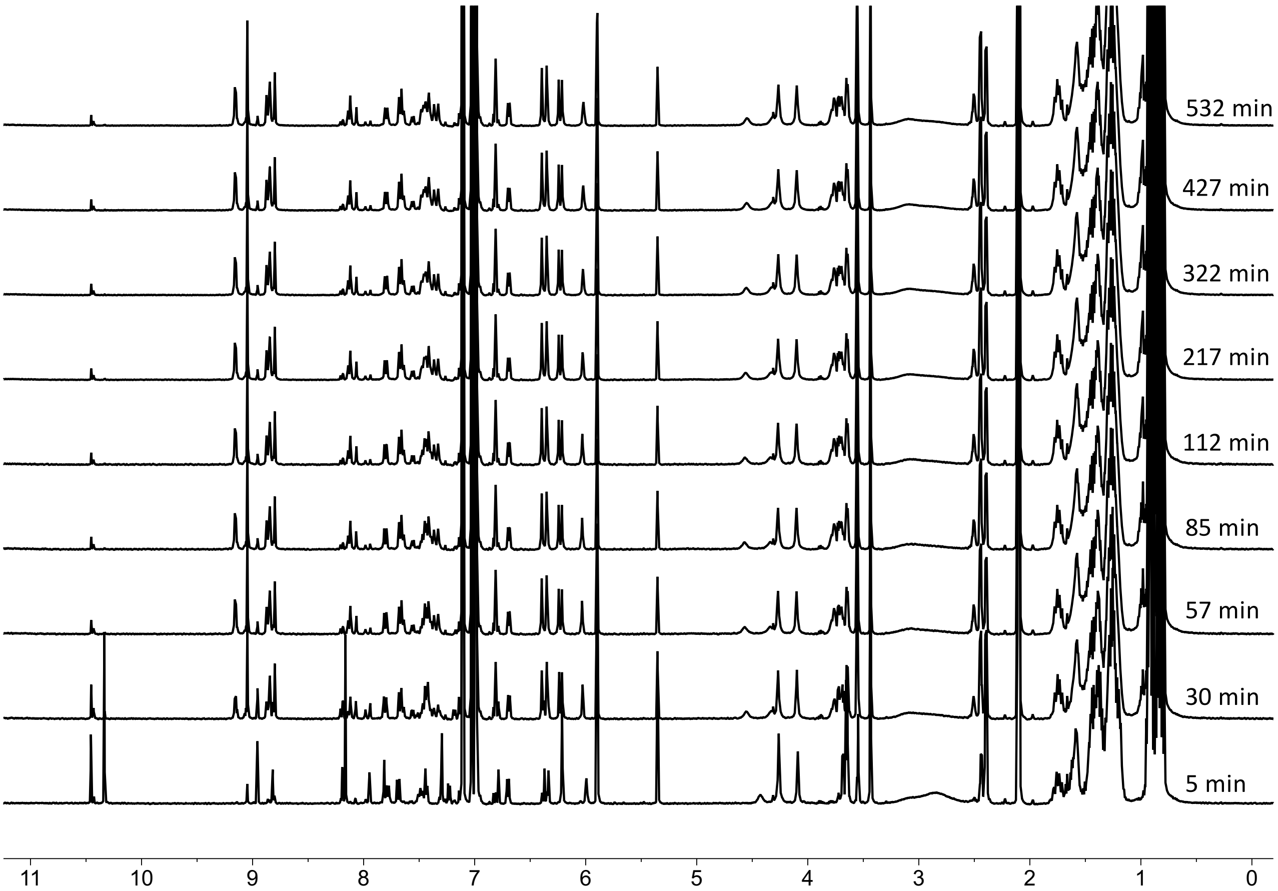

Figure S5. 500 MHz ^{1}H NMR spectra of a mixture of **N** (20 mM) and **D** (10 mM) in toluene-*d*8 in the presence of 3.3 mM **AAA**. On the right side of the Figure Sis reported the time (in minutes) after the addition of **N**.

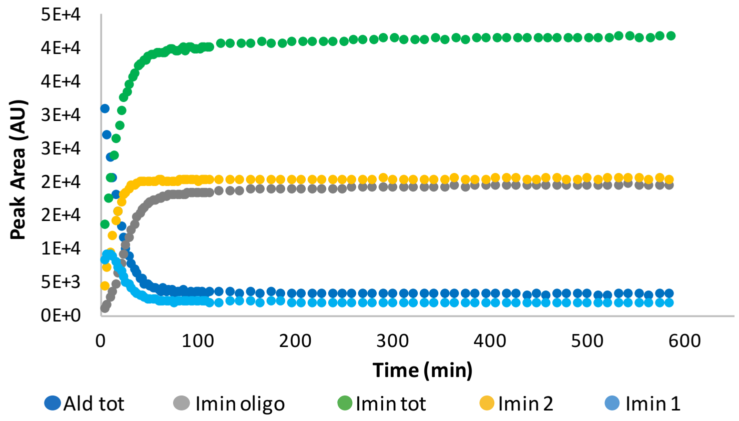

Figure S6. Kinetics of imine formation measured by integration of all imine and aldehyde signals in the 500 MHz ^{1}H NMR spectra (**D** 10 mM, **N** 20 mM, **AAA** 3.3 mM). Total aldehyde signals (**Ald tot**, 10.51-10.26 ppm) and total imine (**Imin tot**, 9.25-8.92 ppm), together with monoimine (**Imin 1**, 8.97-8.93 ppm), monomeric bis imine (**Imin 2**, 9.10-9.00 ppm) and oligomeric imines (**Imin oliogo**, 9.22-9.12 ppm) are integrated and reported.

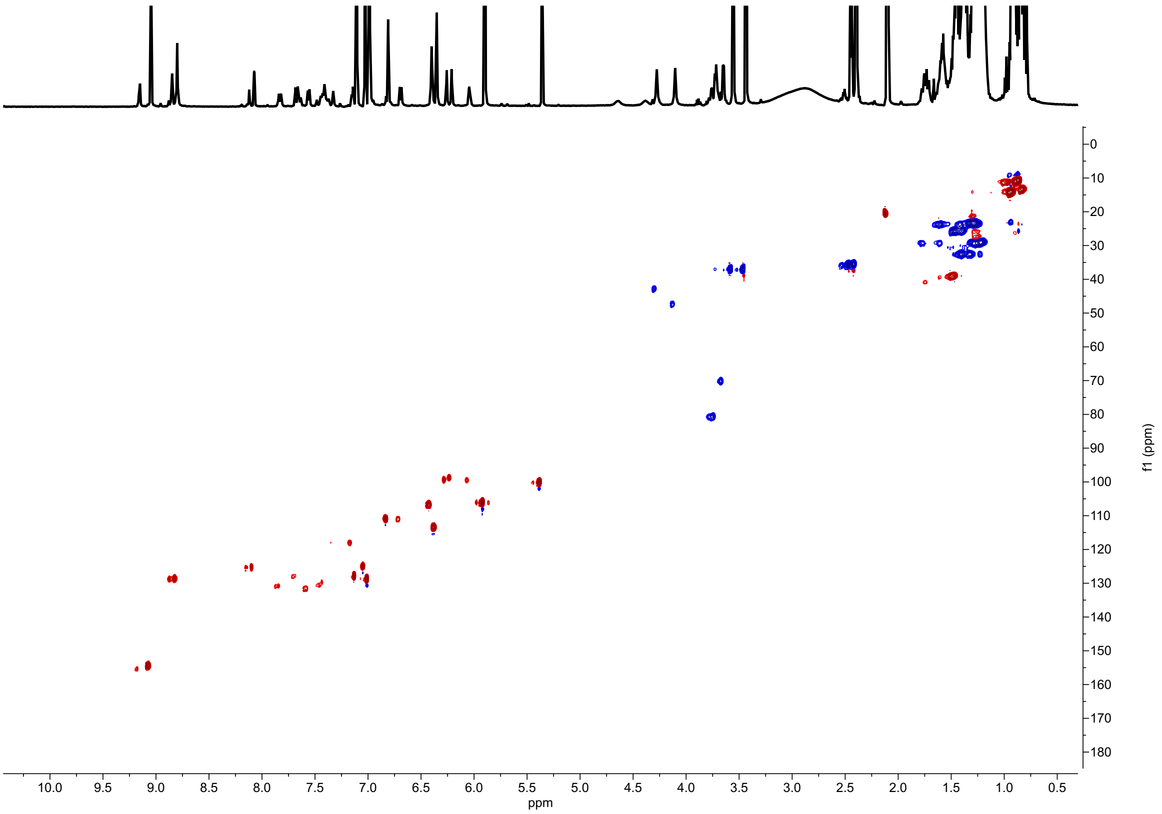

Figure S7. 500 MHz HSQC NMR spectra of a mixture of **N** (20 mM) and **D** (10 mM) in toluene-*d8* in the presence of 3.3 mM **AAA**, after 600 min of reaction.

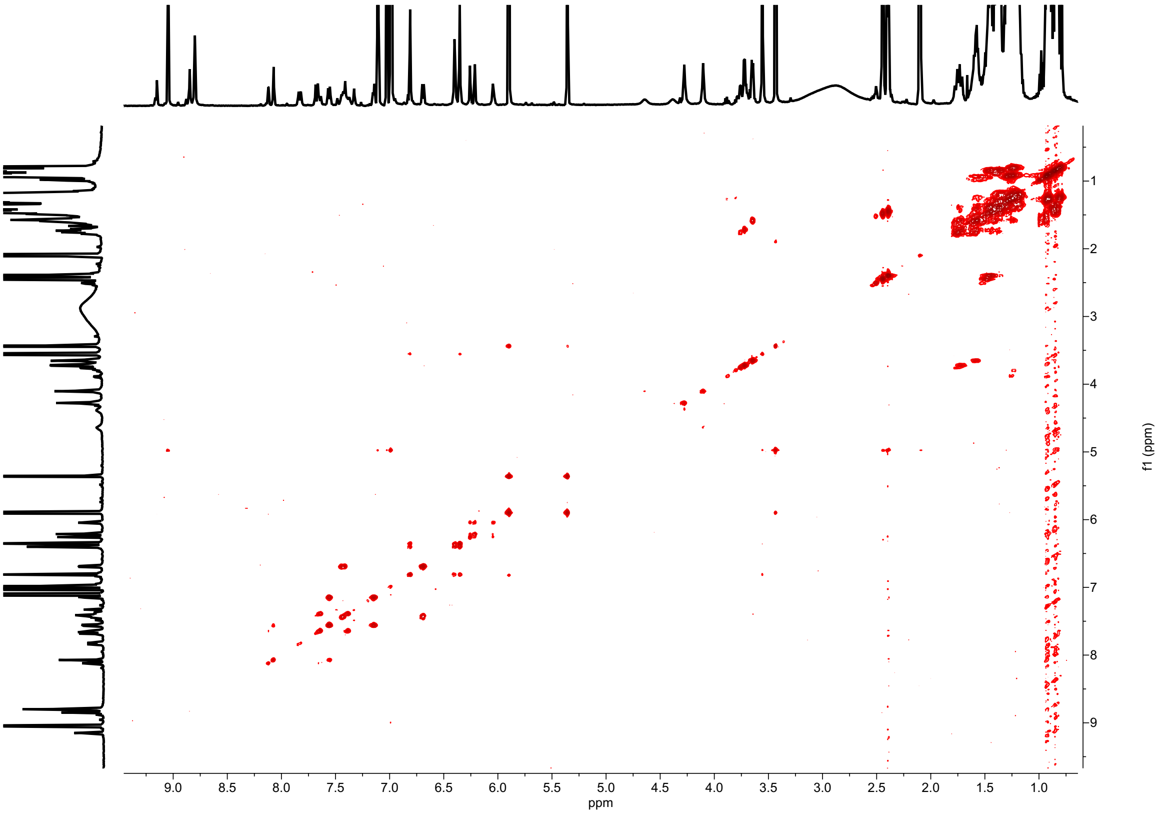

Figure S8. 500 MHz COSY NMR spectra of a mixture of **N** (20 mM) and **D** (10 mM) in toluene-*d8* in the presence of 3.3 mM **AAA**, after 600 min of reaction.

2.2 ^{1}H-NMR Kinetic experiments: Varying the aniline concentration.

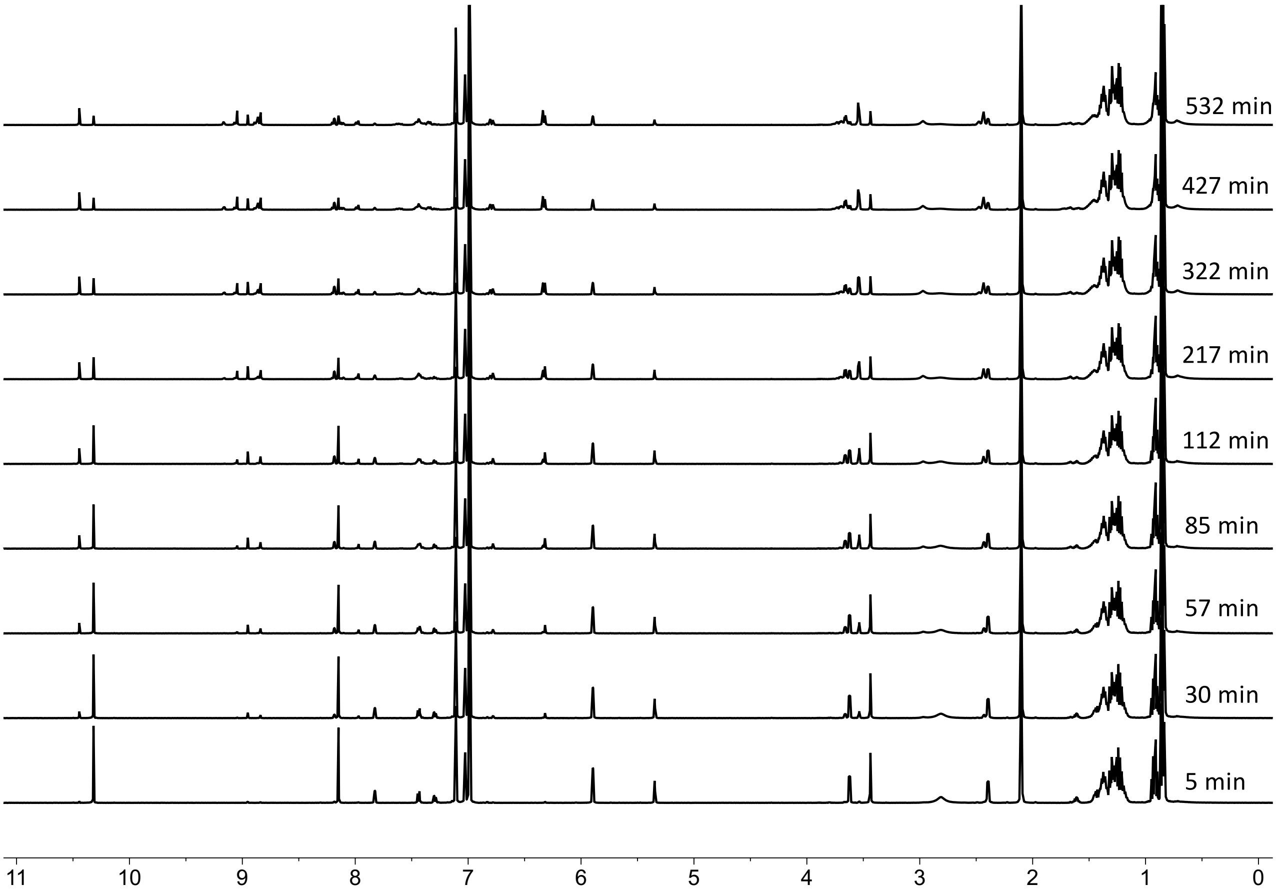

Figure S9. 500 MHz ^{1}H NMR spectra of a mixture of **N** (16 mM) and **D** (10 mM) in toluene-*d*8 in the presence of 10 mM ***n*BuPO$_3$**. On the right side of the figure is reported the time (in minutes) after the addition of **N**.

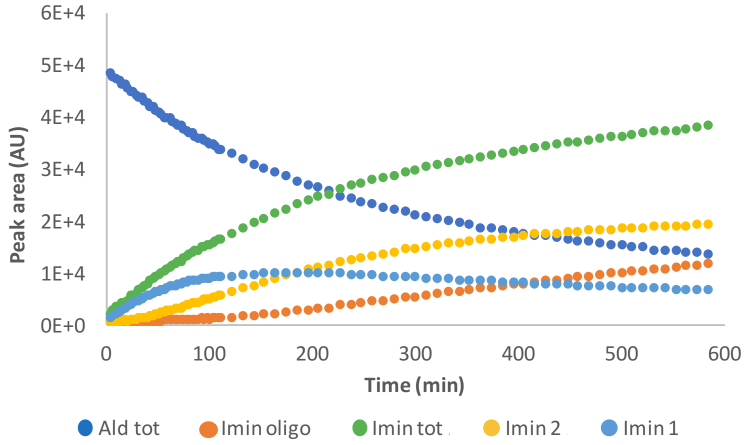

Figure S10. Kinetics of imine formation measured by integration of all imine and aldehyde signals in the 500 MHz ^{1}H NMR spectra (**D** 10 mM, **N** 16 mM, ***n*Bu$_3$PO** 10 mM). Total aldehyde signals (**Ald tot**, 10.51-10.26 ppm) and total imine (**Imin tot**, 9.25-8.92 ppm), together with monoimine (**Imin 1**, 8.97-8.93 ppm), monomeric bis imine (**Imin 2**, 9.10-9.00 ppm) and oligomeric imines (**Imin oliogo**, 9.22-9.12 ppm) are integrated and reported.

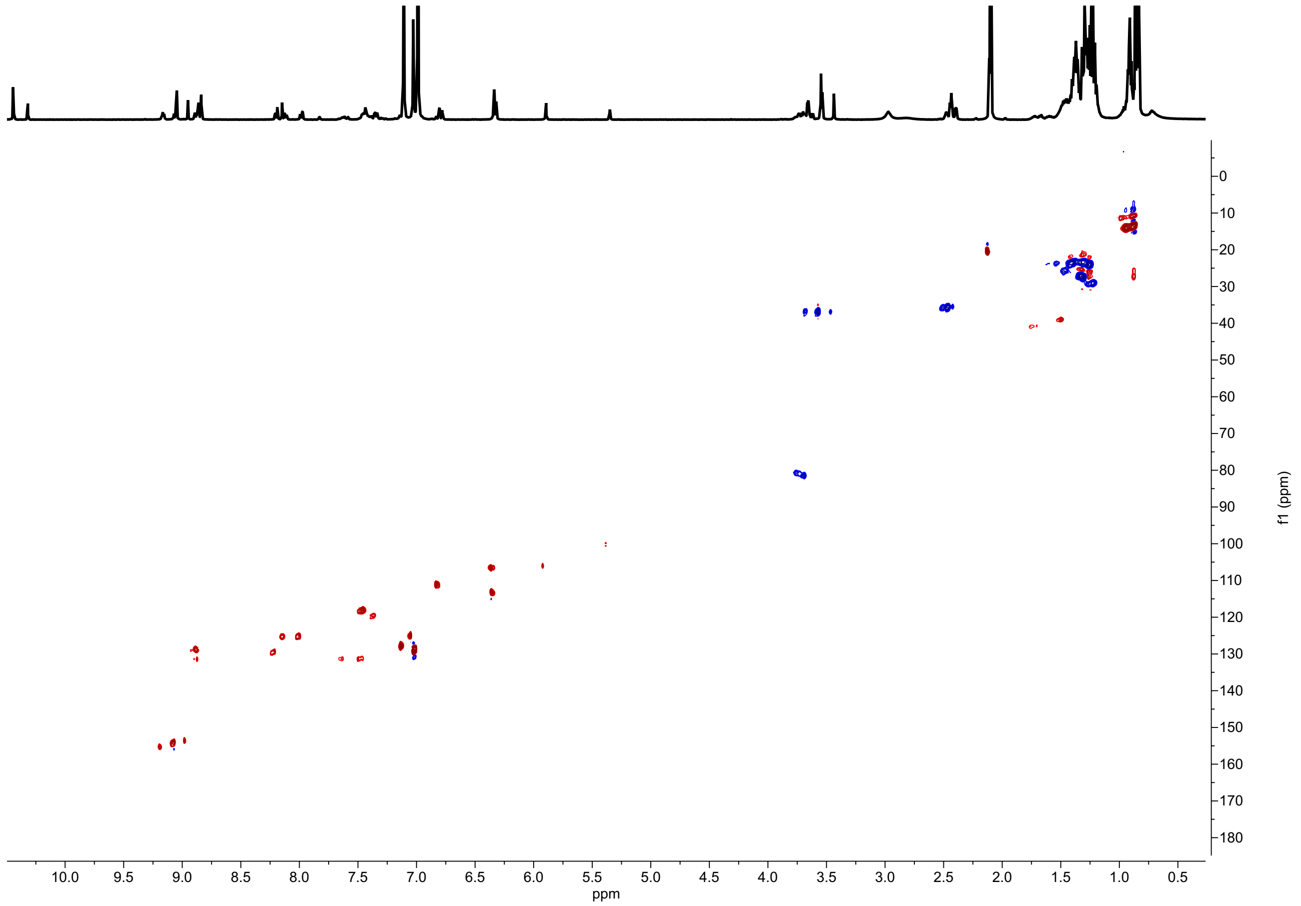

Figure S11. 500 MHz HSQC NMR spectra of a mixture of **N** (16 mM) and **D** (10 mM) in toluene-*d*8 in the presence of 10 mM ***n*BuPO$_3$**, after 600 min of reaction.

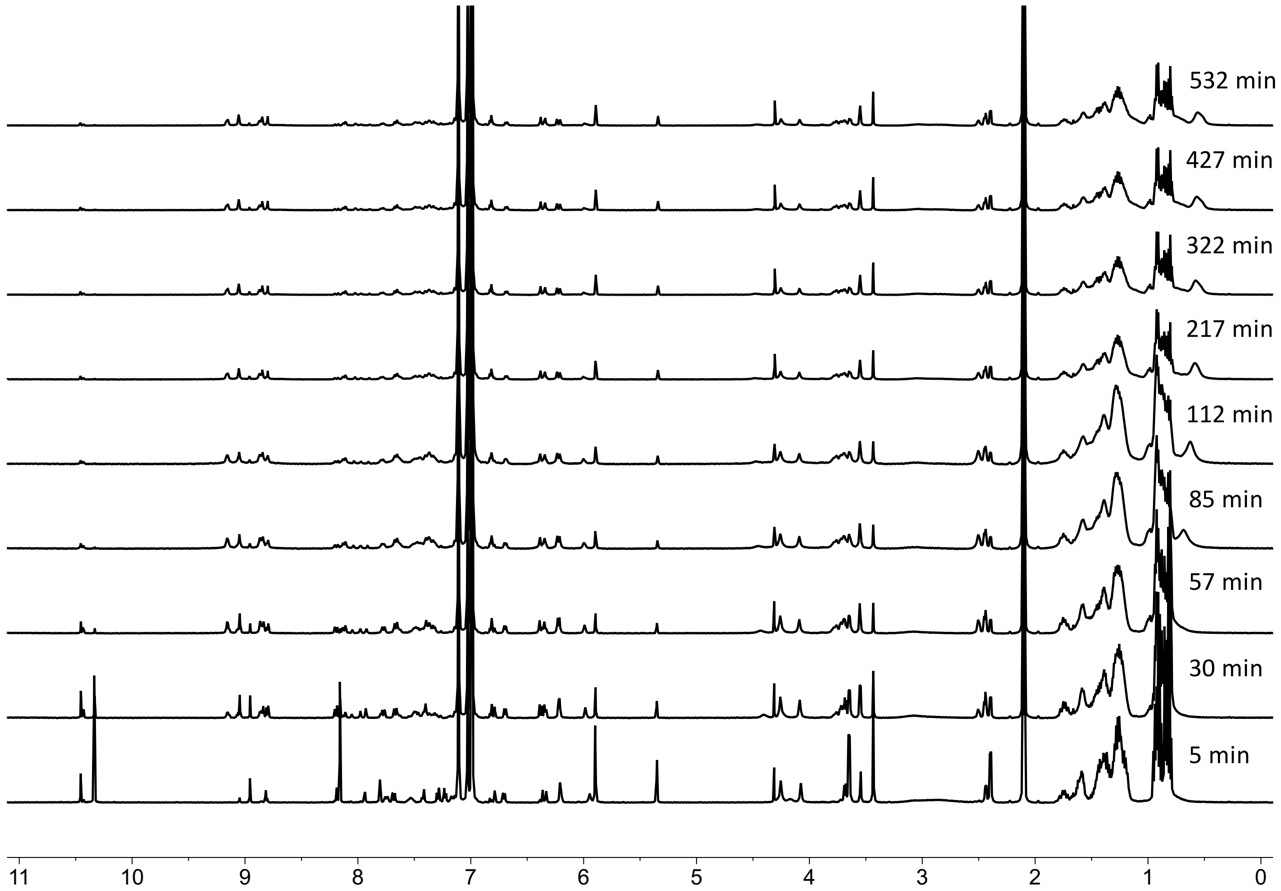

Figure S12. 500 MHz ^{1}H NMR spectra of a mixture of **N** (16 mM) and **D** (10 mM) in toluene-*d*8 in the presence of 3.3 mM **AAA**. On the right side of the figure is reported the time after the addition of **N**.

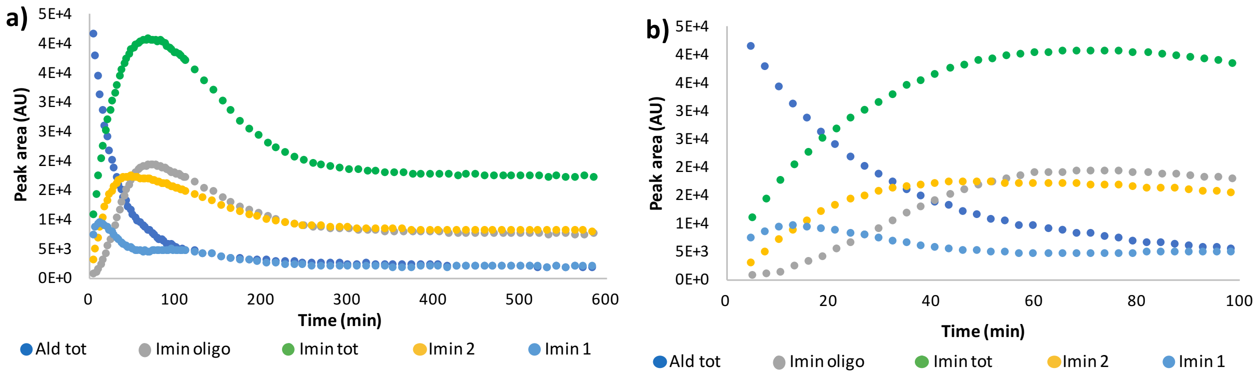

Figure S13. a) Kinetics of imine formation measured by integration of all imine and aldehyde signals in the 500 MHz ^{1}H NMR spectra (**D** 10 mM, **N** 16 mM, **AAA** 3.3 mM). Total aldehyde signals (**Ald tot**, 10.51-10.26 ppm) and total imine (**Imin tot**, 9.25-8.92 ppm), together with monoimine (**Imin 1**, 8.97-8.93 ppm), monomeric bis imine (**Imin 2**, 9.10-9.00 ppm) and oligomeric imines (**Imin oliogo**, 9.22-9.12 ppm) are integrated and reported. The decreasing of total imine after 100 min is due to precipitation of longer insoluble oligomers. **b)** A zoom of the initial 100 min of reaction is reported.

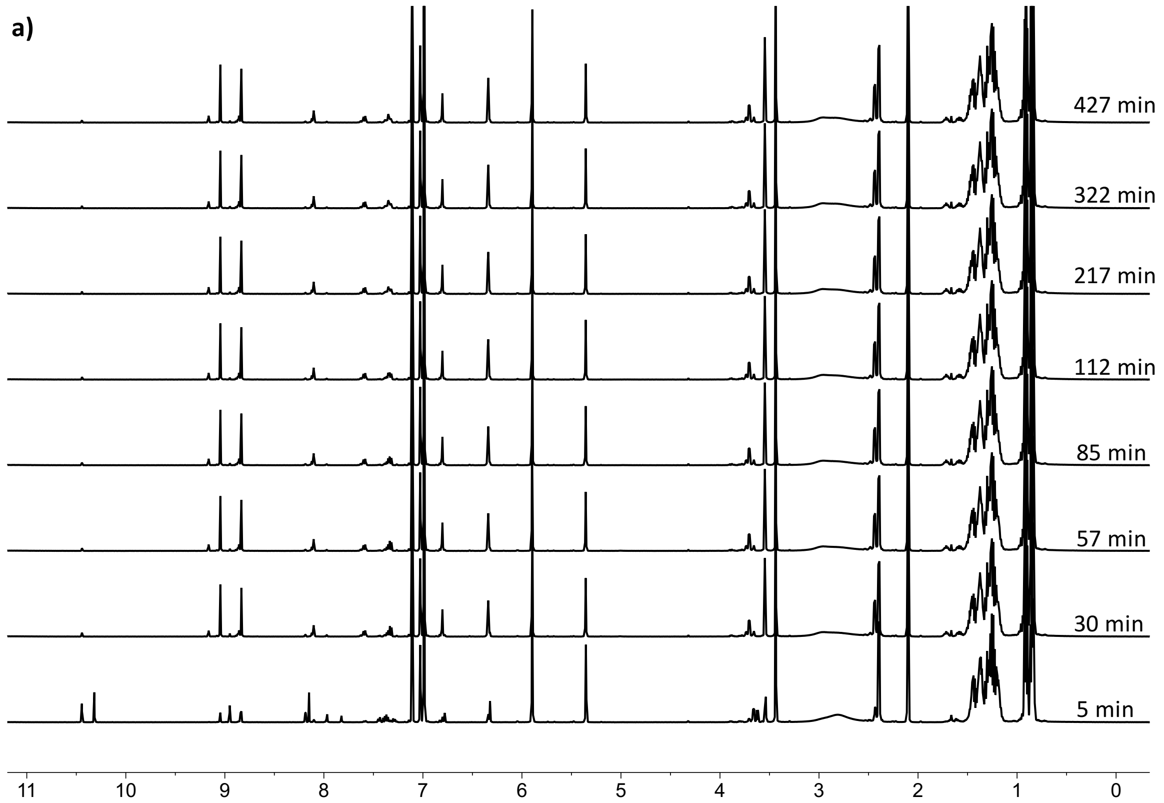

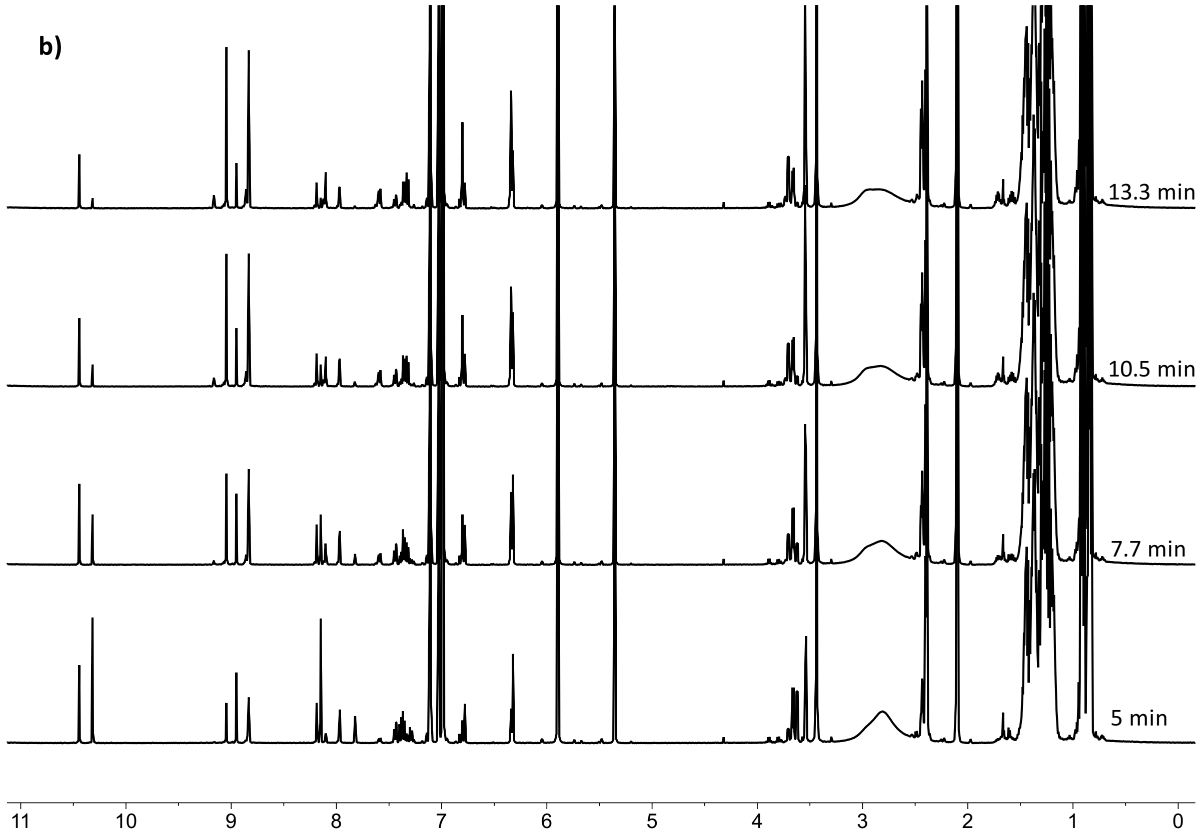

Figure S14. a) 500 MHz ^{1}H NMR spectra of a mixture of **N** (50 mM) and **D** (10 mM) in toluene-*d*8 in the presence of 10 mM ***n*BuPO₃**. On the right side of the figure is reported the time after the addition of **N**. **b)** A zoom of the initial 13 minutes of reaction is reported.

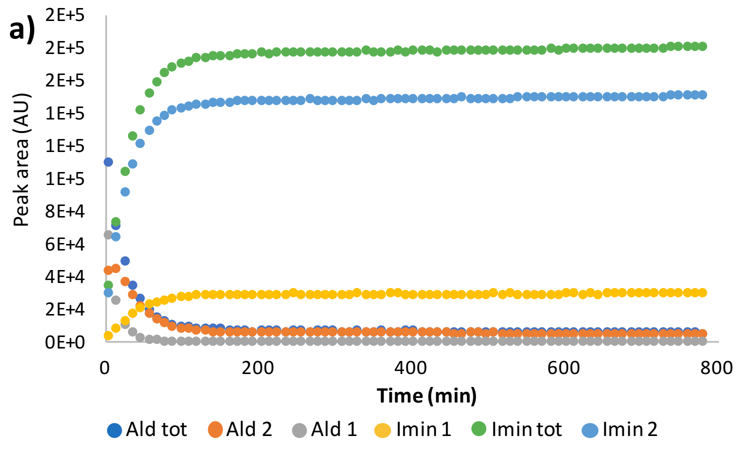

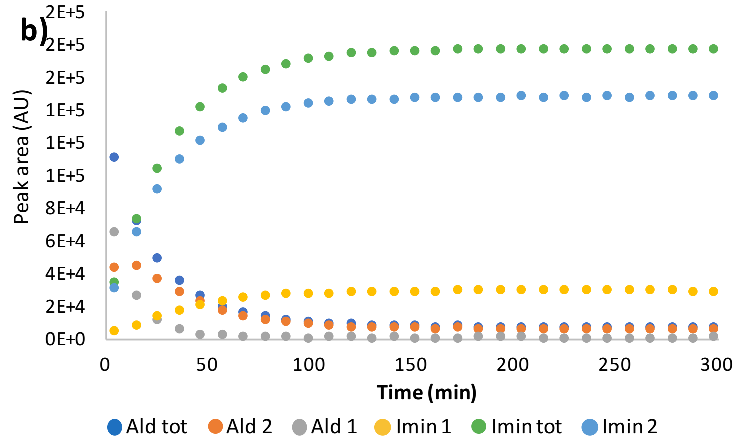

Figure S15. a) Kinetics of imine formation measured by integration of all imine and aldehyde signals in the 500 MHz ^{1}H NMR spectra (**D** 10 mM, **N** 50 mM, ***n*****Bu$_3$PO** 10 mM). Total aldehyde signals (**Ald tot**, 10.51-10.26 ppm) and total imine (**Imin tot**, 9.25-8.92 ppm), together with monoimine (**Imin 1**, 8.97-8.93 ppm), monomeric bis imine (**Imin 2**, 9.10-9.00 ppm) and oligomeric imines (**Imin oliogo**, 9.22-9.12 ppm) are integrated and reported. **b)** A zoom of the initial 300 min of reaction.

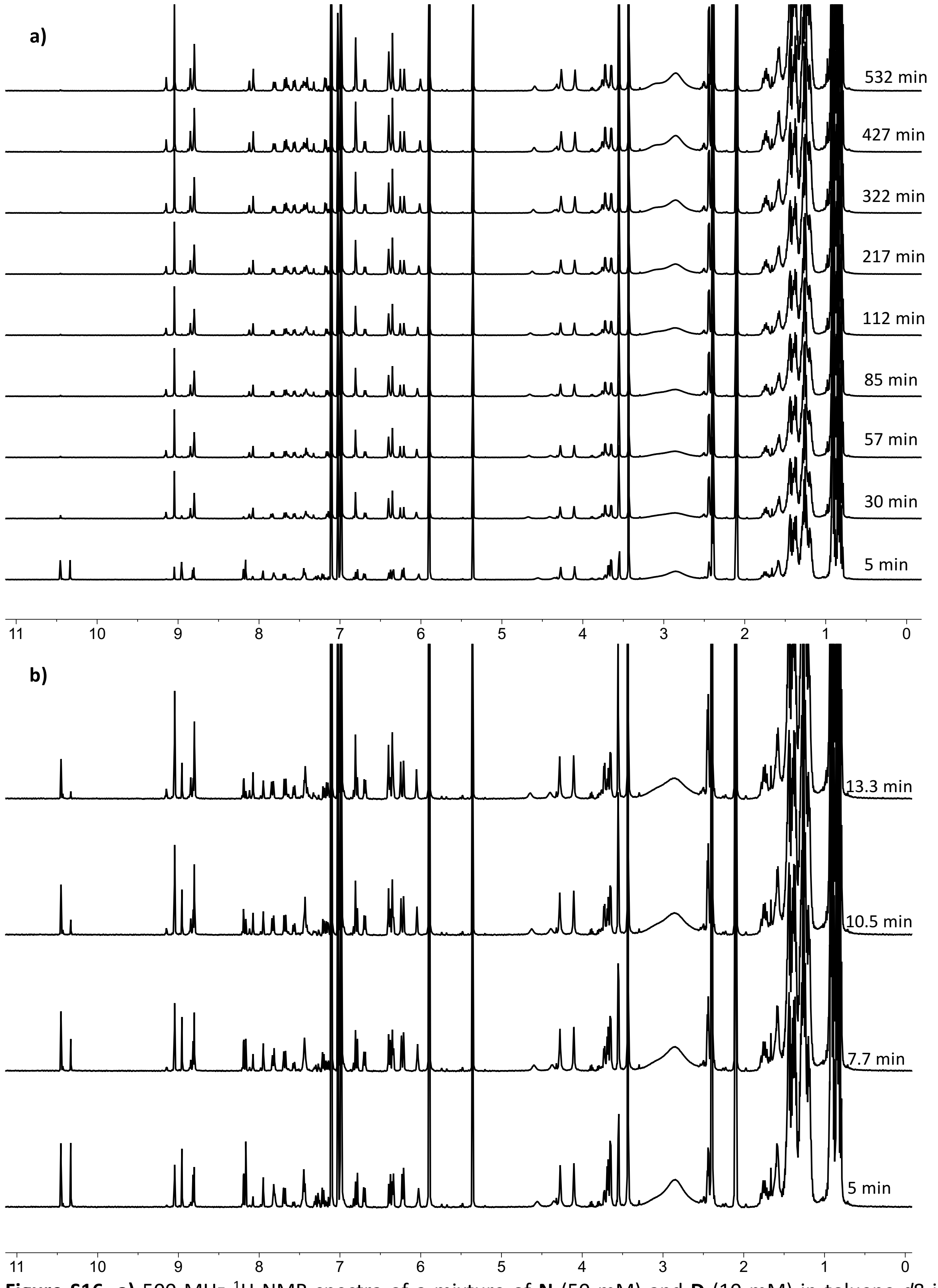

Figure S16. a) 500 MHz ^{1}H NMR spectra of a mixture of **N** (50 mM) and **D** (10 mM) in toluene-*d*8 in the presence of 3.3 mM **AAA**. On the right side of the figure is reported the time after the addition of **N**. **b)** A zoom of the initial 13 minutes of reaction is reported.

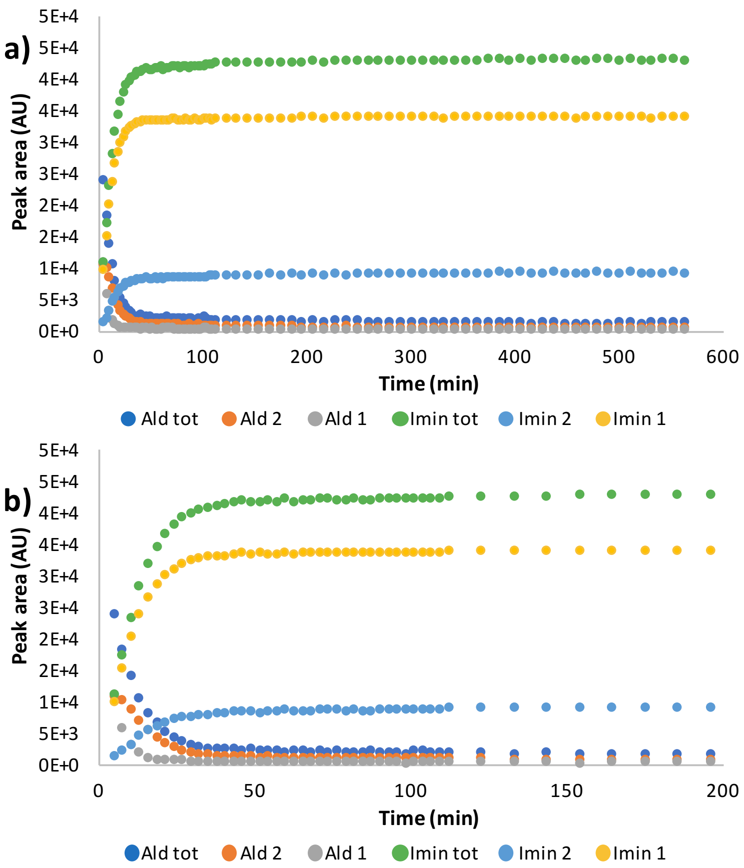

Figure S17. a) Kinetics of imine formation measured by integration of all imine and aldehyde signals in the 500 MHz ^{1}H NMR spectra (**D** 10 mM, **N** 50 mM, **AAA** 3.3 mM). Total aldehyde signals (**Ald tot**, 10.51-10.26 ppm) and total imine (**Imin tot**, 9.25-8.92 ppm), together with monoimine (**Imin 1**, 8.97-8.93 ppm), monomeric bis imine (**Imin 2**, 9.10-9.00 ppm) and oligomeric imines (**Imin oliogo**, 9.22-9.12 ppm) are integrated and reported. **b)** A zoom of the initial 200 min of reaction.

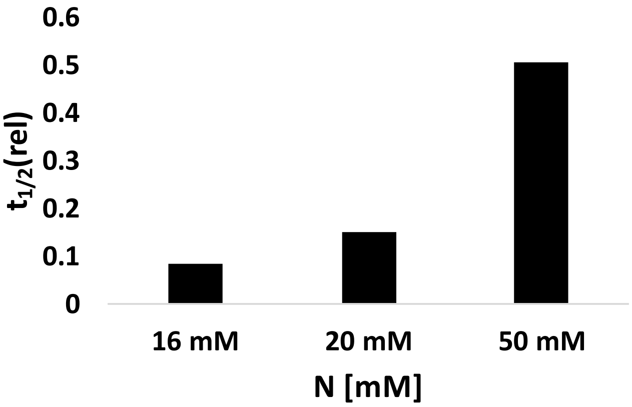

Figure S18. Aniline effect on the template ability of trimer **AAA**. Half-life for reactions in the presence of **AAA** (3.3 mM) relative to the corresponding half-life in the presence of *n*Bu$_3$PO (10 mM) ($t_{1/2}$(rel)). Half-lives were measured by integration of the 500 MHz ^{1}H NMR signal due to the aldehyde as a function of time for mixtures of 10 mM **D** and a variable amount of aniline (16, 20 or 50 mM **N**) in toluene-*d*8.

2.3 ^{1}H-NMR Kinetic experiments: Varying the concentration of AAA.

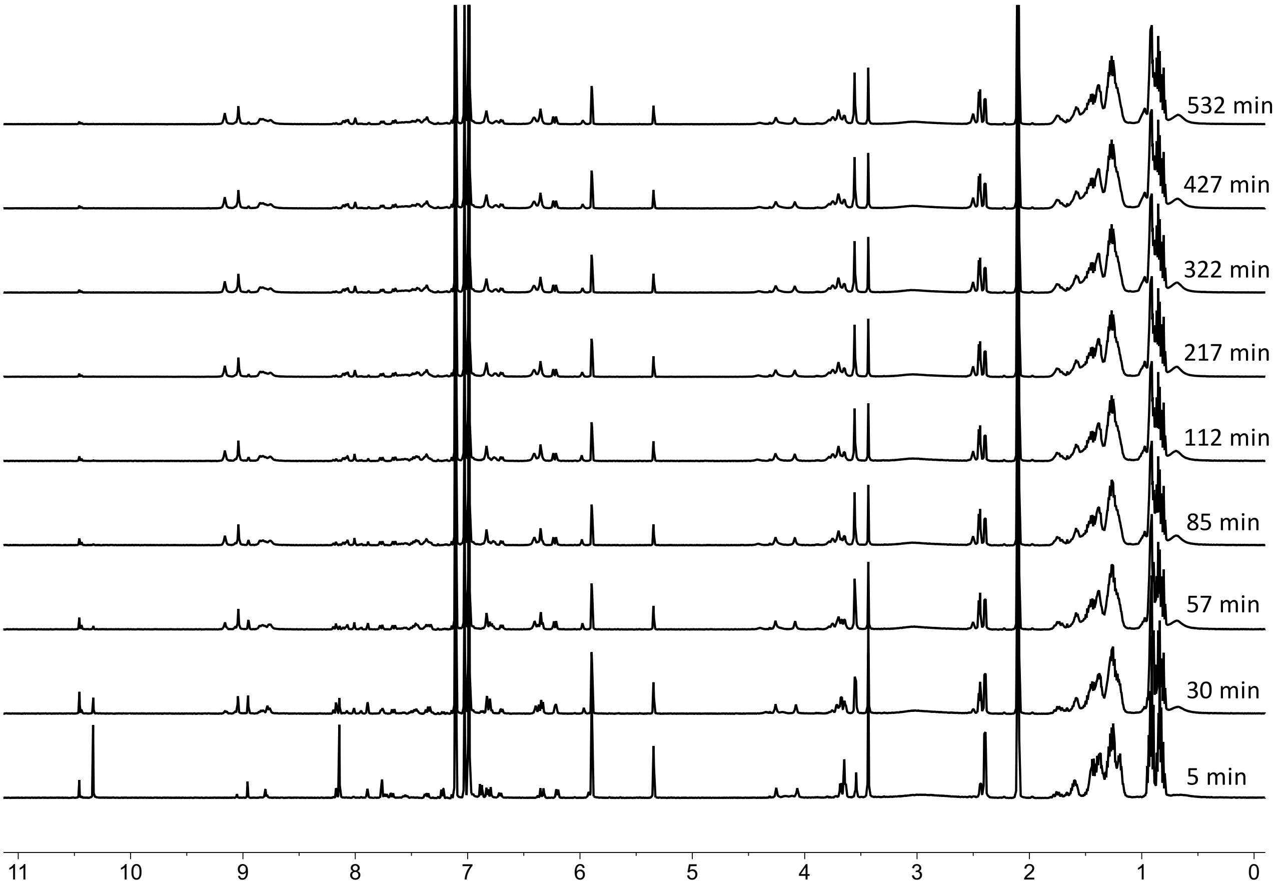

Figure S19. 500 MHz ^{1}H NMR spectra of a mixture of **N** (20 mM) and **D** (10 mM) in toluene-*d*8 in the presence of 1.65 mM **AAA.** On the right side of the figure is reported the time after the addition of **N**.

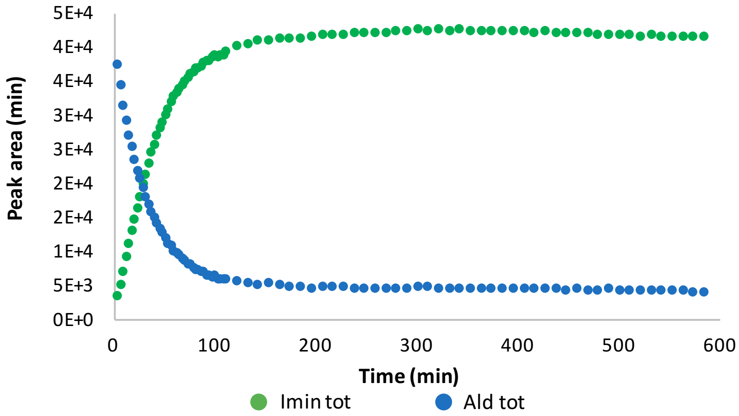

Figure S20 Kinetics of imine formation measured by integration of all imine and aldehyde signals in the 500 MHz ^{1}H NMR spectra (**D** 10 mM, **N** 20 mM, **AAA** 1,65 mM). Total aldehyde signals (**Ald tot**, 10.51-10.26 ppm) and total imine (**Imin tot**, 9.25-8.92 ppm) are integrated and reported.

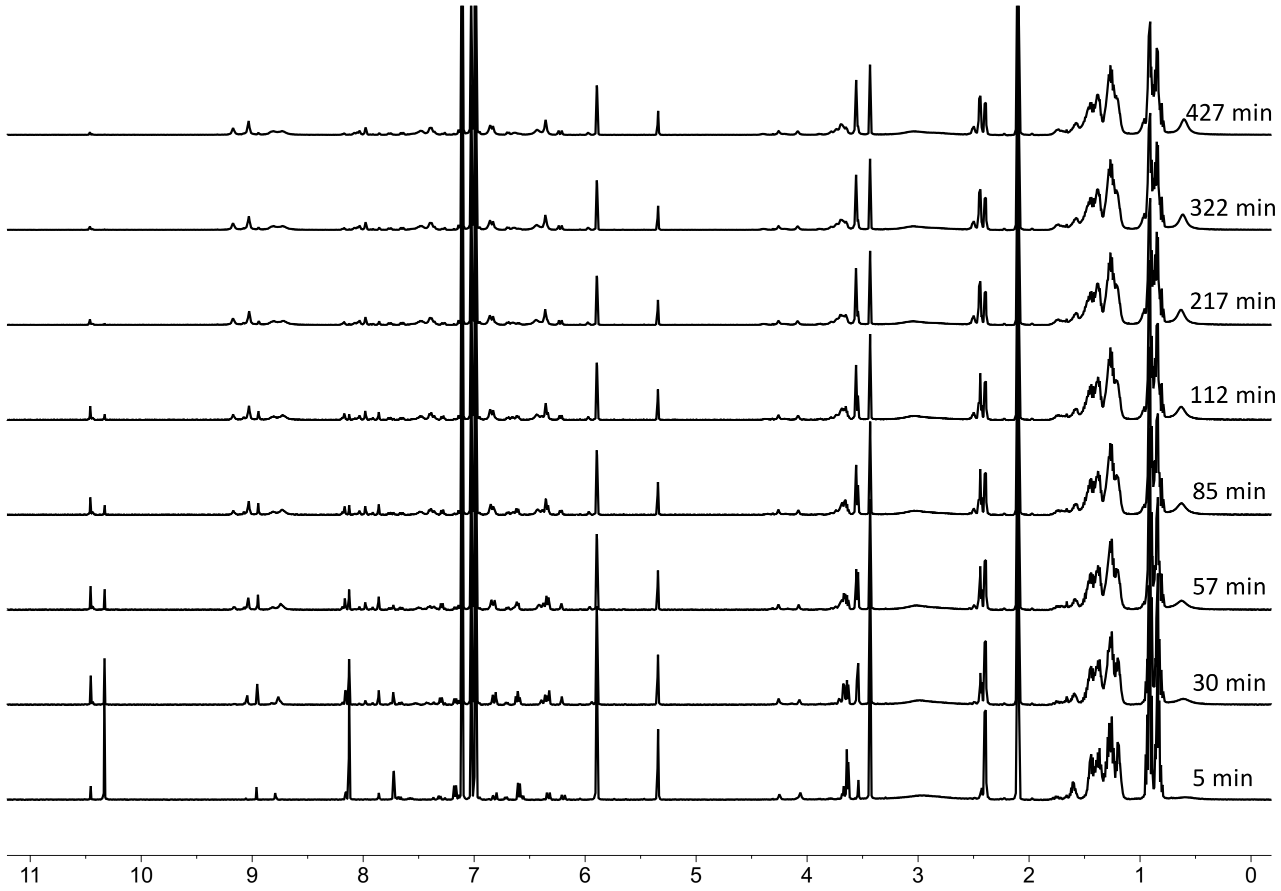

Figure S21. 500 MHz ^{1}H NMR spectra of a mixture of **N** (20 mM) and **D** (10 mM) in toluene-*d*8 in the presence of 0.83 mM **AAA.** On the right side of the figure is reported the time after the addition of **N**.

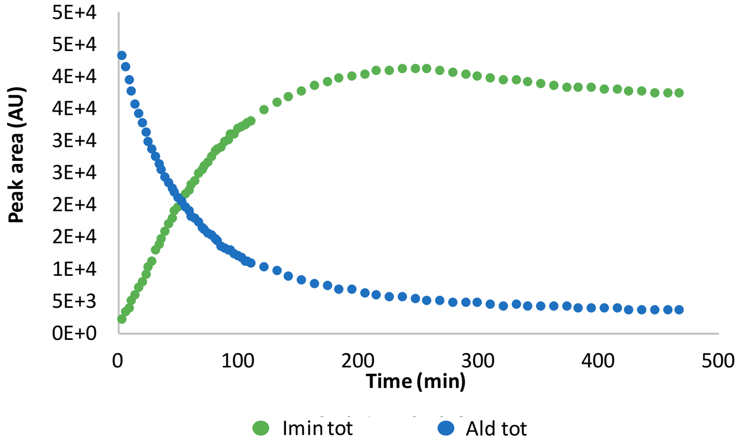

Figure S22. Kinetics of imine formation measured by integration of all imine and aldehyde signals in the 500 MHz ^{1}H NMR spectra (**D** 10 mM, **N** 20 mM, **AAA** 0.83 mM). Total aldehyde signals (**Ald tot**, 10.51-10.26 ppm) and total imine (**Imin tot**, 9.25-8.92 ppm) are integrated and reported. The decreasing of the total imine after 250 min, is because of the low solubility of D oligoimines, in the presence of a low amount of acceptor counterpart.

2.4 ^{1}H-NMR Kinetic experiments: D-N vs AAA - Competition experiment.

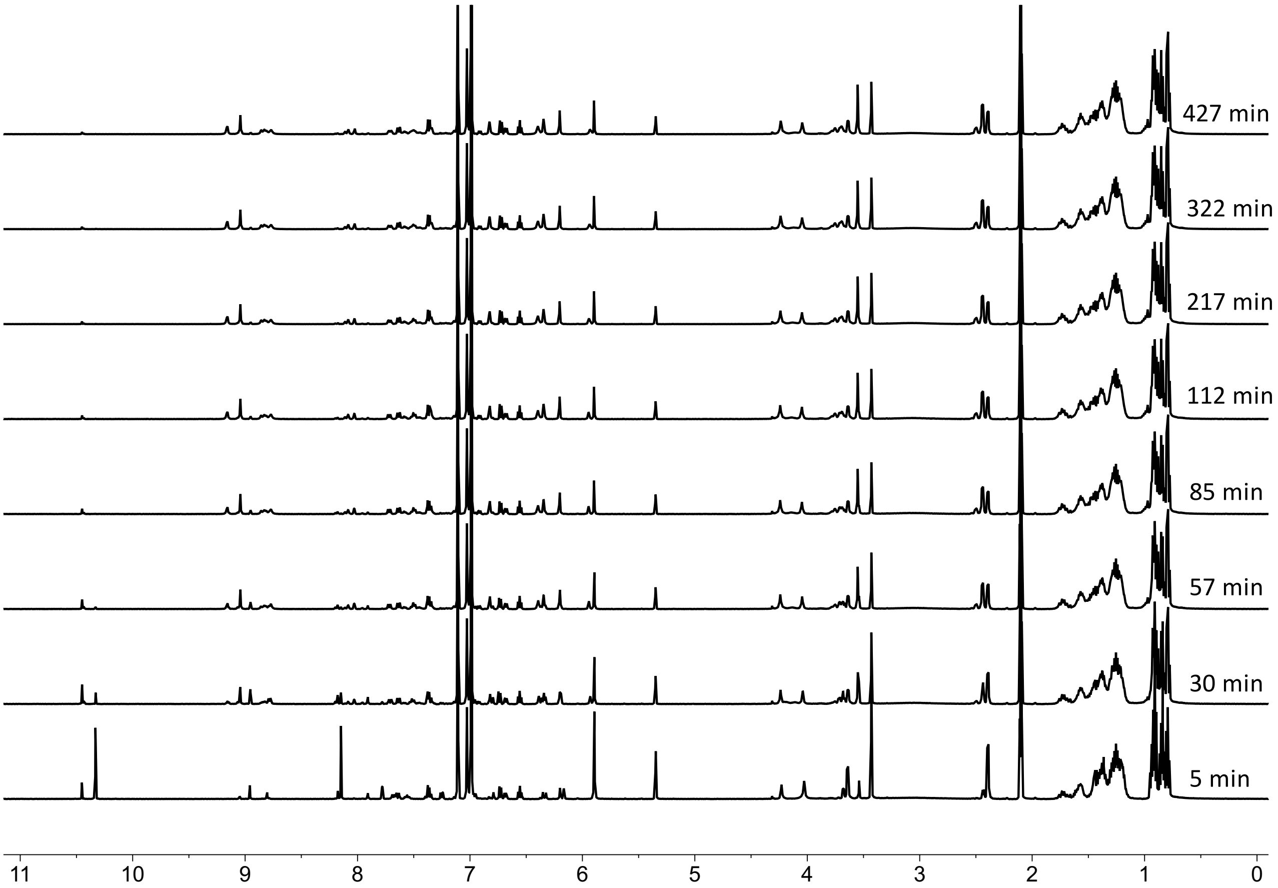

Figure S23. 500 MHz ^{1}H NMR spectra of a mixture of **N** (20 mM) and **D** (10 mM) in toluene-*d*8 in the presence of 3.3 mM **AAA** and 10 mM 2-trifluoromethyl phenol. On the right side of the figure is reported the time after the addition of **N**.

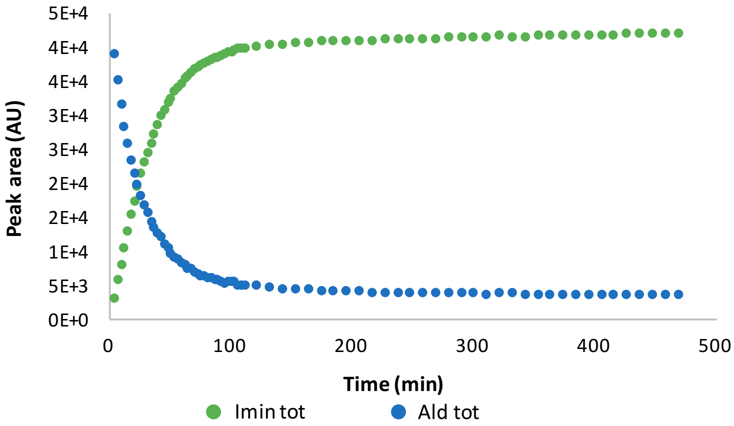

Figure S24 Kinetics of imine formation measured by integration of all imine and aldehyde signals in the 500 MHz ^{1}H NMR spectra (**D** 10 mM, **N** 16 mM, **AAA** 3.3 mM, **CF_3PhOH** 10 mM). Total aldehyde signals (**Ald tot**, 10.51-10.26 ppm) and total imine (**Imin tot**, 9.25-8.92 ppm) are integrated and reported.

2.5 ^{1}H-NMR Kinetic experiments: D-N'

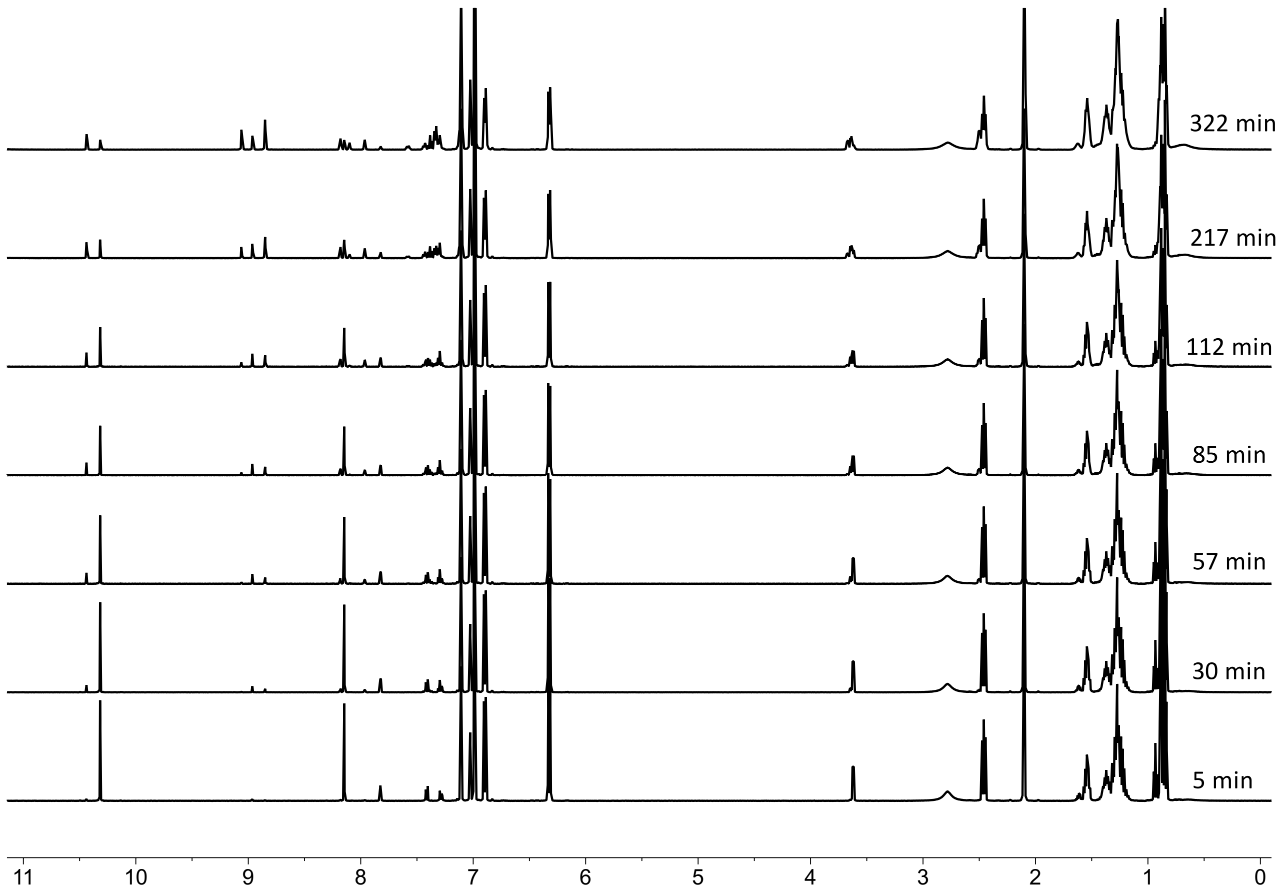

Figure S25. 500 MHz ^{1}H NMR spectra of a mixture of **N'** (40 mM) and **D** (10 mM) in toluene-*d*8 in the presence of 10 mM ***n*Bu₃PO.** On the right side of the figure is reported the time after the addition of **N**.

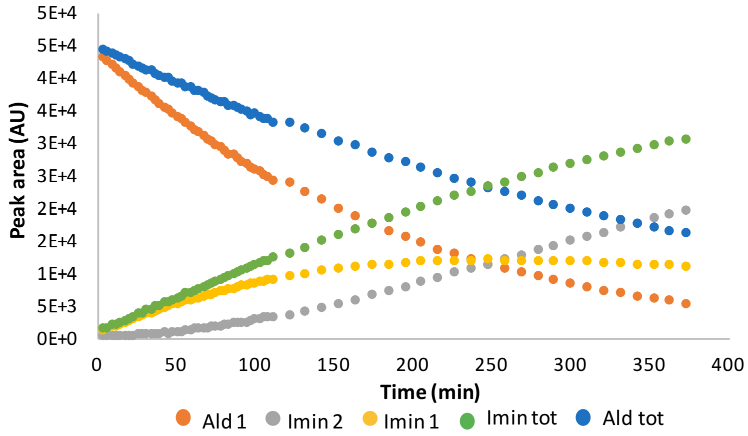

Figure S26. Kinetics of imine formation measured by integration of all imine and aldehyde signals in the 500 MHz ^{1}H NMR spectra (**D** 10 mM, **N'** 40 mM, ***n*Bu₃PO** 10 mM). Monoaldehyde (**Ald 1**, 10.37-10.28 ppm), total aldehyde signals (**Ald tot**, 10.48-10.26 ppm) and total imine (**Imin tot**, 9.11-8.92 ppm), together with monoimine (**Imin 1**, 8.99-8.93 ppm), monomeric bis imine (**Imin 2**, 9.10-9.03 ppm) are integrated and reported.

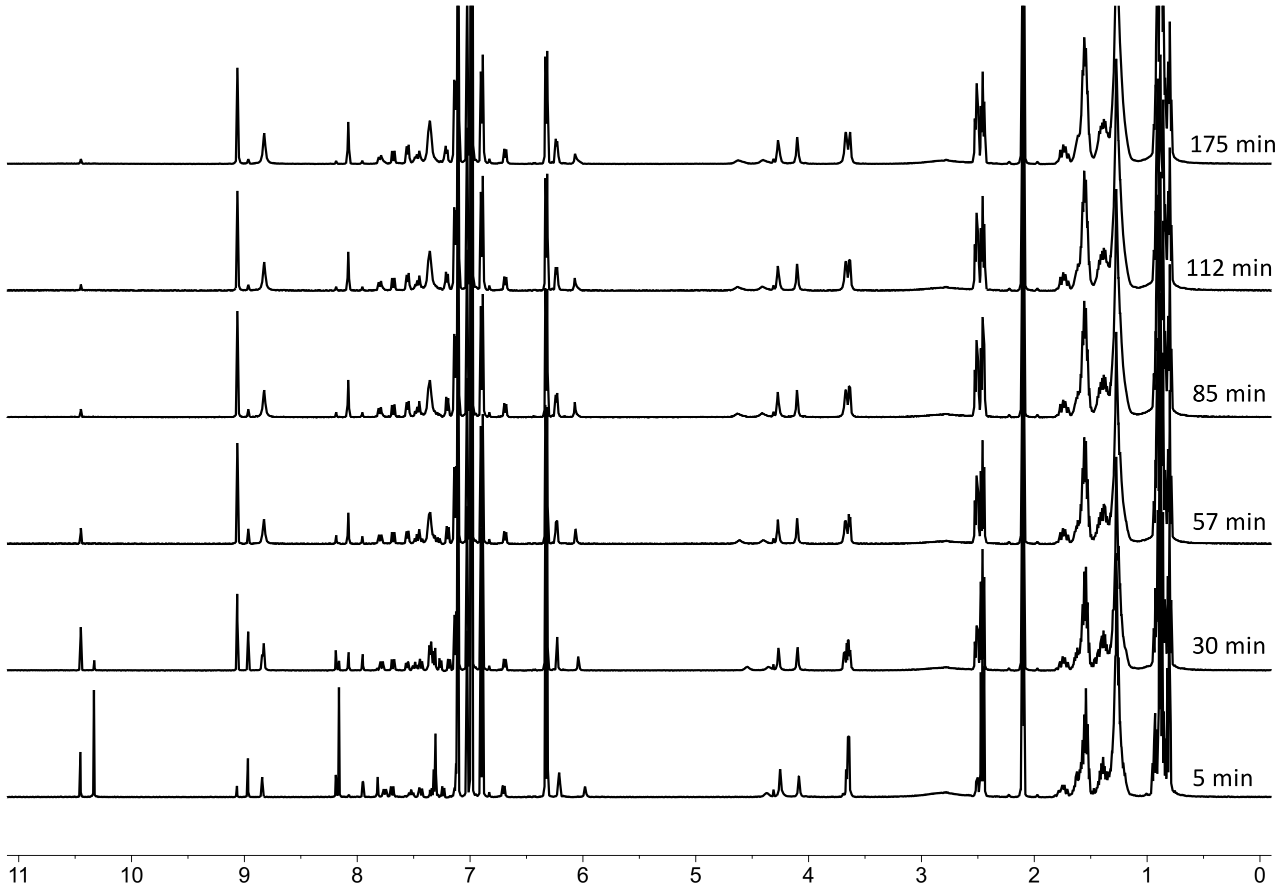

Figure S27. 500 MHz ^{1}H NMR spectra of a mixture of **N'** (40 mM) and **D** (10 mM) in toluene-*d8* in the presence of 3.3 mM **AAA.** On the right side of the figure is reported the time after the addition of **N**.

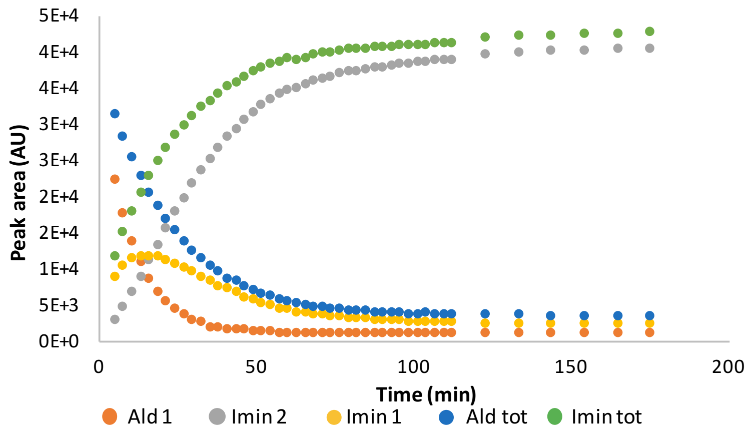

Figure S28. Kinetics of imine formation measured by integration of all imine and aldehyde signals in the 500 MHz ^{1}H NMR spectra (**D** 10 mM, **N'** 40 mM, **AAA** 3.3 mM). Monoaldehyde (**Ald 1**, 10.37-10.28 ppm), total aldehyde signals (**Ald tot**, 10.48-10.26 ppm) and total imine (**Imin tot**, 9.11-8.92 ppm), together with monoimine (**Imin 1**, 8.99-8.93 ppm), monomeric bis imine (**Imin 2**, 9.10-9.03 ppm) are integrated and reported.

2.6 ^{1}H-NMR Kinetic experiments: D'-N

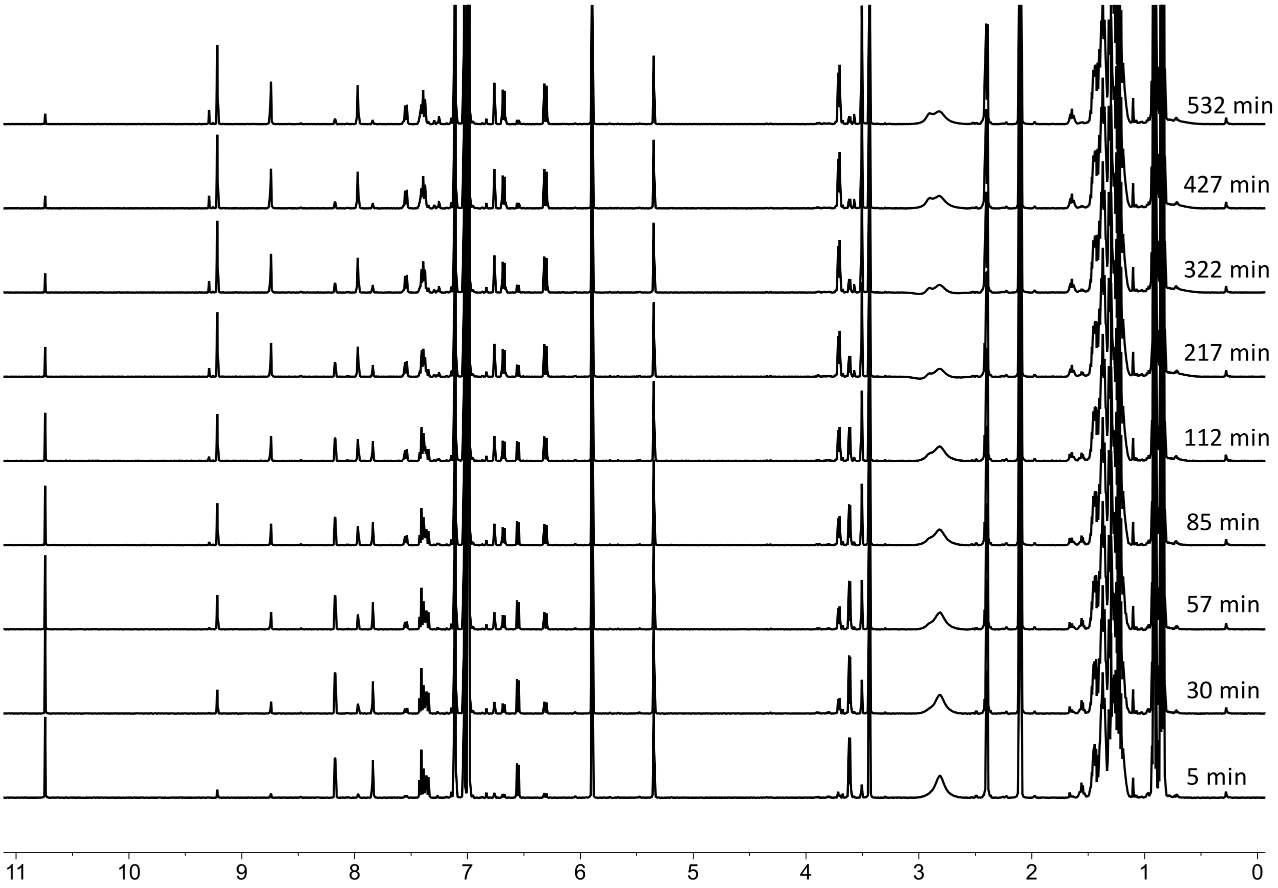

Figure S29. 500 MHz ^{1}H NMR spectra of a mixture of **N** (20 mM) and **D'** (10 mM) in toluene-*d*8 in the presence of 10 mM ***n*Bu₃PO.** On the right side of the figure is reported the time after the addition of **N**.

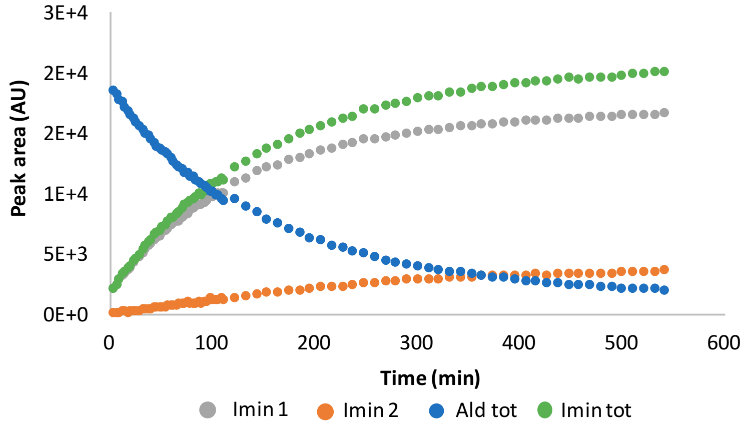

Figure S30. Kinetics of imine formation measured by integration of all imine and aldehyde signals in the 500 MHz ^{1}H NMR spectra (**D'** 10 mM, **N** 20 mM, ***n*Bu$_3$PO** 10 mM). Aldehyde signal (**Ald tot**, 10.77-10.71 ppm) and total imine (**Imin tot**, 9.18-9.32 ppm), together with monoimine (**Imin 1**, 9.24 – 9.19 ppm), dimeric imine (**Imin 2**, 9.31-9.24 ppm) are integrated and reported.

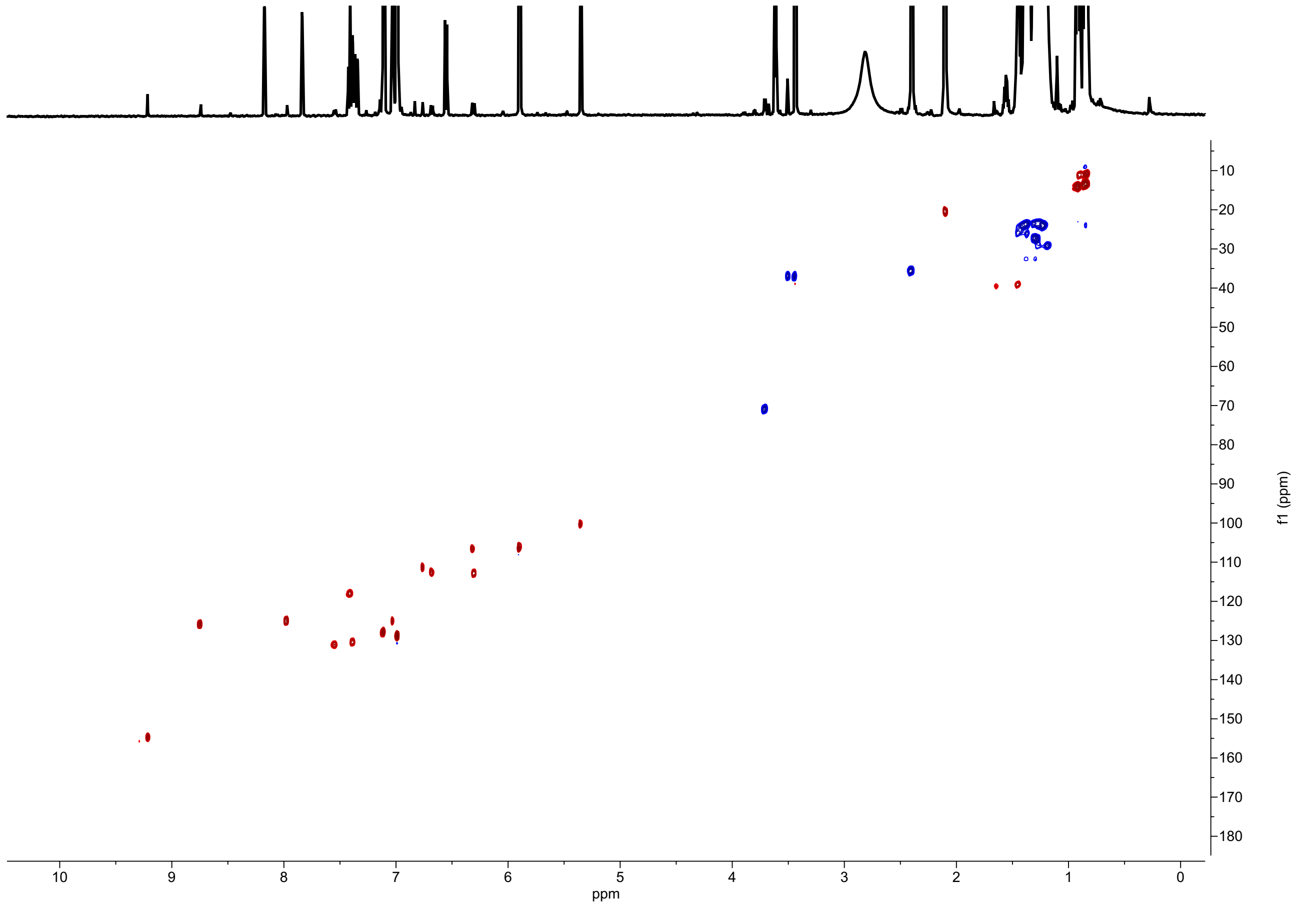

Figure S31. 500 MHz HSQC NMR spectra of a mixture of **N** (20 mM) and **D'** (10 mM) in toluene-*d*8 in the presence of 10 mM ***n*BuPO$_3$**, after 600 min of reaction.

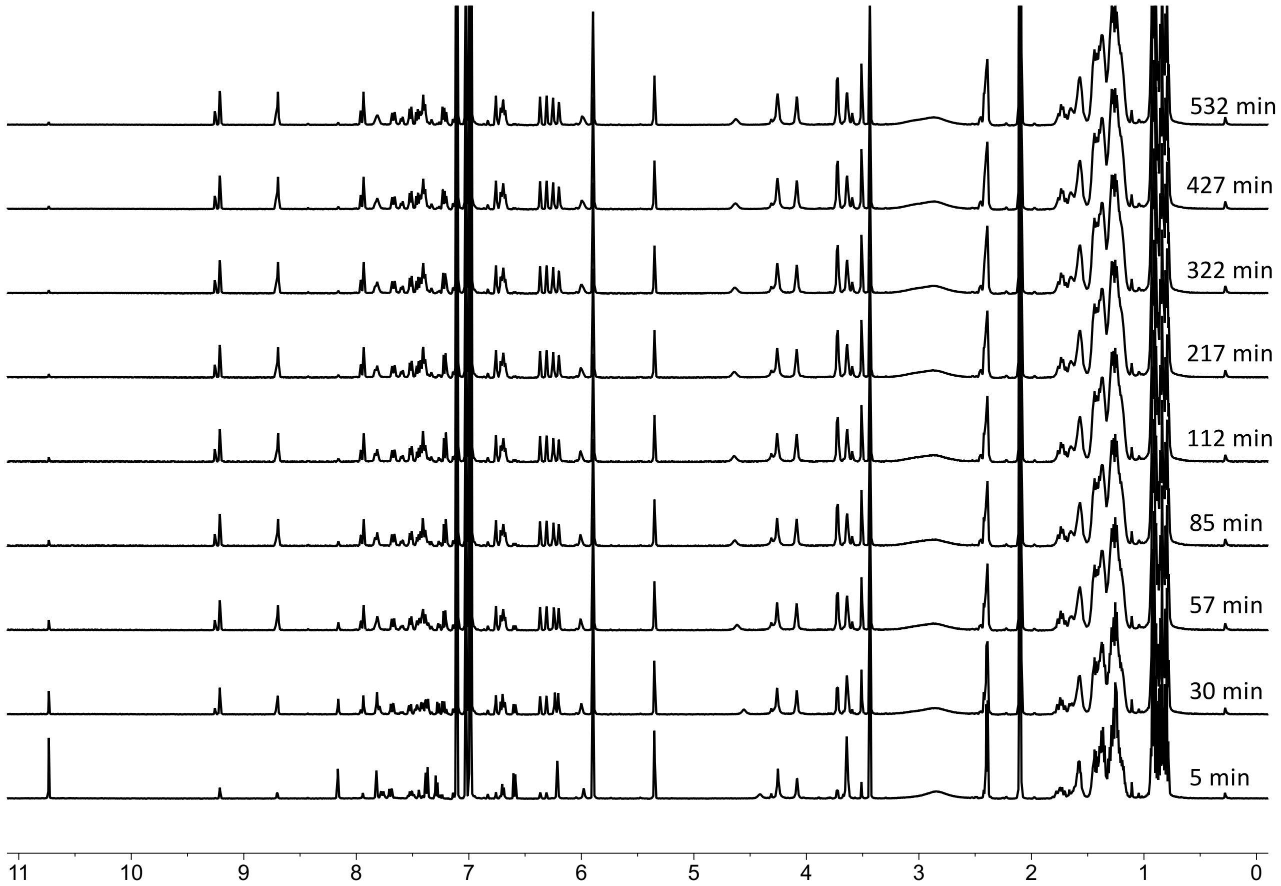

Figure S32. 500 MHz ^{1}H NMR spectra of a mixture of **N** (20 mM) and **D'** (10 mM) in toluene-*d*8 in the presence of 3.3 mM **AAA.** On the right side of the figure is reported the time after the addition of **N**.

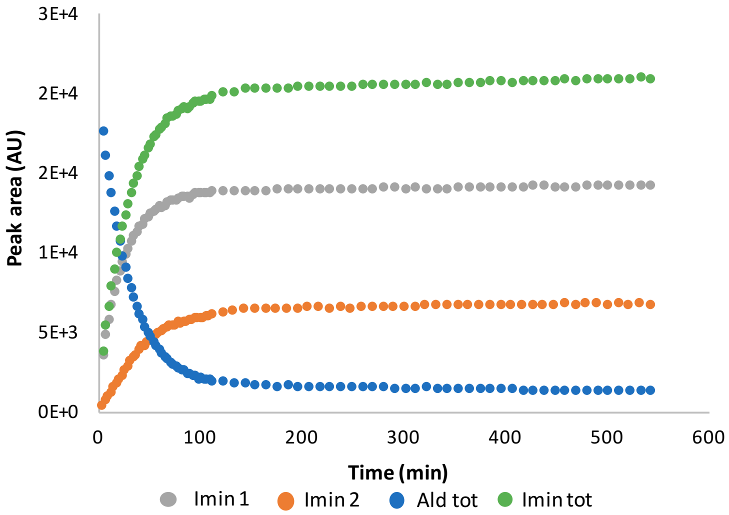

Figure S33. Kinetics of imine formation measured by integration of all imine and aldehyde signals in the 500 MHz ^{1}H NMR spectra (**D'** 10 mM, **N** 20 mM, **AAA** 3.3 mM). Aldehyde signal (**Ald tot**, 10.77-10.71 ppm) and total imine (**Imin tot**, 9.18-9.32 ppm), together with monoimine (**Imin 1**, 9.24 – 9.19 ppm), dimeric imine (**Imin 2**, 9.31-9.24 ppm) are integrated and reported.

2.7 ^{1}H-NMR Kinetic experiment: D'-N'

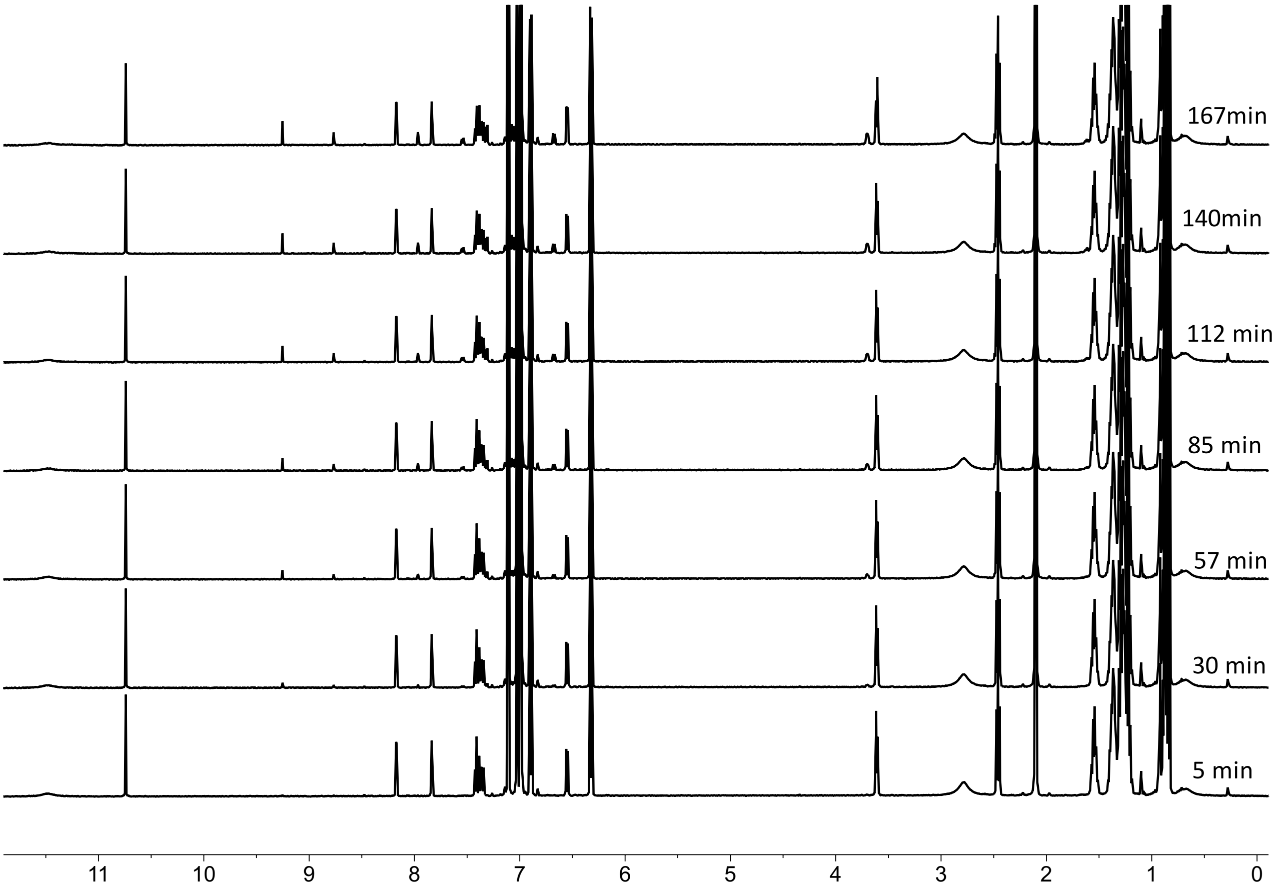

Figure S34. 500 MHz ^{1}H NMR spectra of a mixture of **N'** (40 mM) and **D'** (10 mM) in toluene-*d*8 in the presence of 10 mM ***n*Bu$_3$PO.** On the right side of the figure is reported the time after the addition of **N'**.

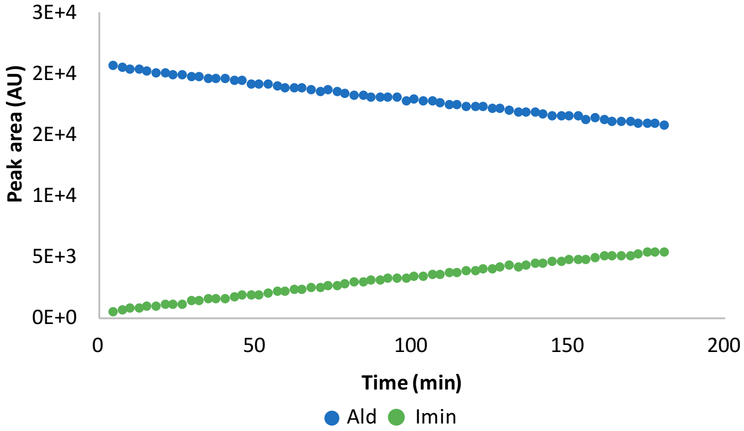

Figure S35. Kinetics of imine formation measured by integration of all imine and aldehyde signals in the 500 MHz ^{1}H NMR spectra (**D'** 10 mM, **N'** 40 mM, ***n*Bu$_3$PO** 10 mM). Aldehyde signals (**Ald tot**, 10.78-10.72 ppm) and imine (**Imin tot**, 9.27-9.23 ppm) signals are integrated and reported.

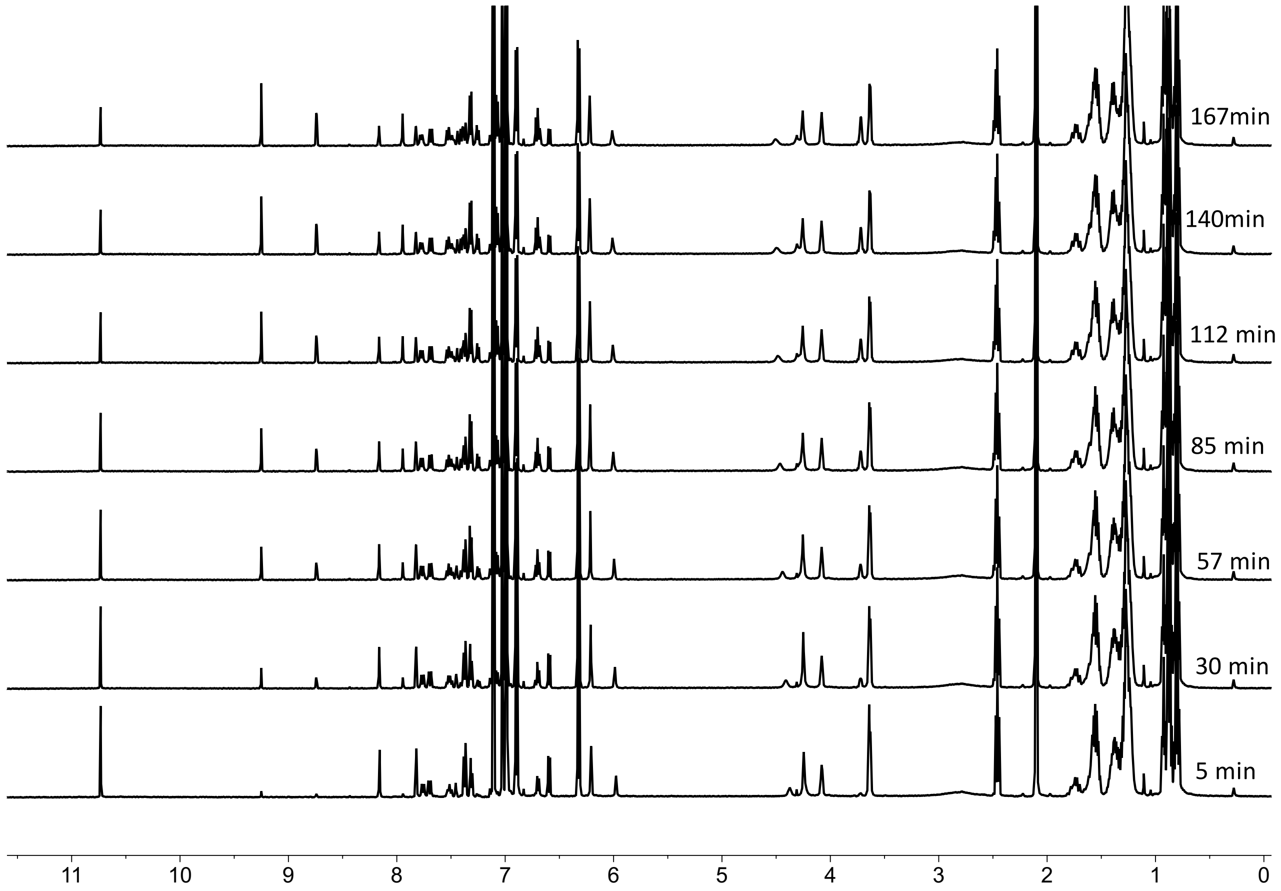

Figure S36. 500 MHz ^{1}H NMR spectra of a mixture of **N'** (40 mM) and **D'** (10 mM) in toluene-*d*8 in the presence of 3.3 mM **AAA.** On the right side of the figure is reported the time after the addition of **N'**.

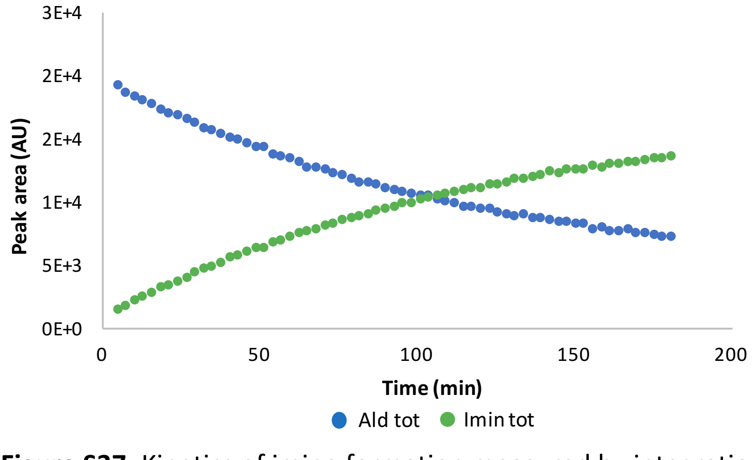

Figure S37. Kinetics of imine formation measured by integration of all imine and aldehyde signals in the 500 MHz ^{1}H NMR spectra (**D'** 10 mM, **N'** 40 mM, **AAA** 3.3 mM). Aldehyde signals (**Ald tot**, 10.78-10.72 ppm) and imine (**Imin tot**, 9.27-9.23 ppm) signals are integrated and reported.

2.8 ^{1}H-NMR Kinetic experiments: P-N'

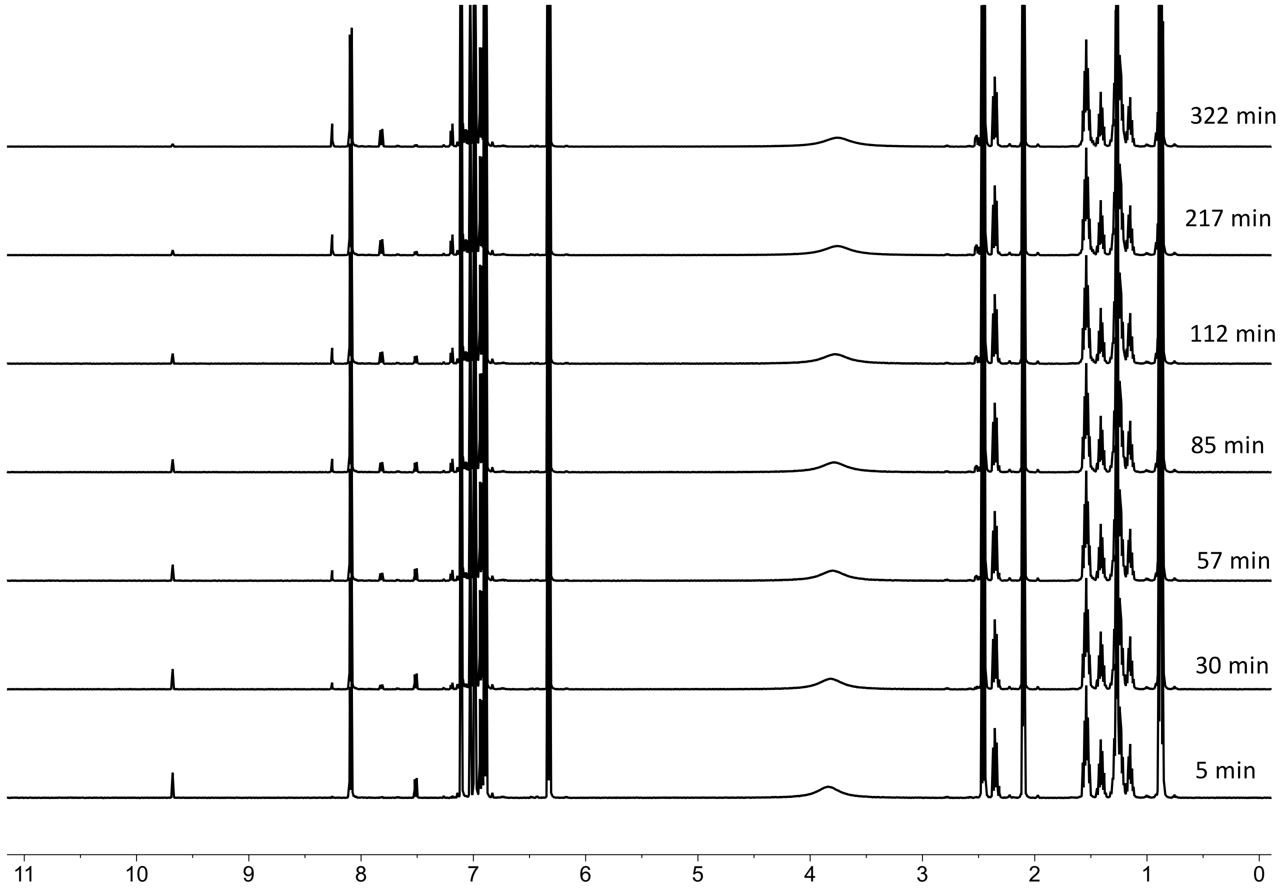

Figure S38. 500 MHz ^{1}H NMR spectra of a mixture of **N'** (40 mM) and **P** (10 mM) in toluene-*d*8 (**Ctr** experiment)**.** On the right side of the figure is reported the time after the addition of **N'**.

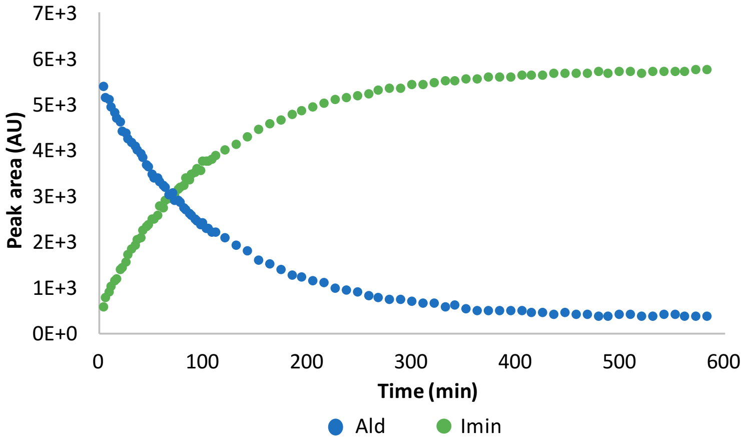

Figure S39. Kinetics of imine formation measured by integration of imine and aldehyde signals in the 500 MHz ^{1}H NMR spectra (**P** 10 mM, **N'** 40 mM). Aldehyde (**Ald**, 9,69 - 9,67 ppm) and imine (**Imin**, 8.27-8.25 ppm) signals are integrated and reported.

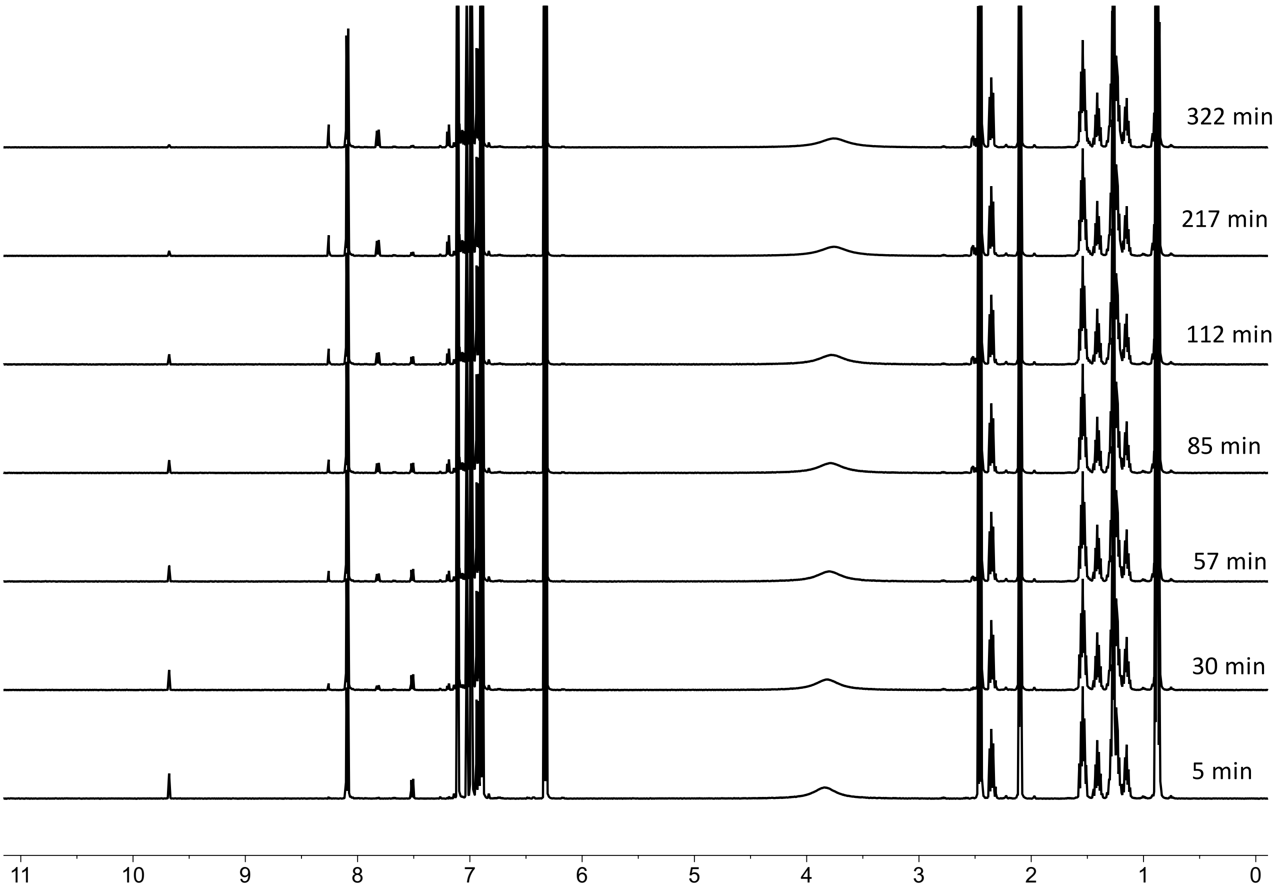

Figure S40. 500 MHz ^{1}H NMR spectra of a mixture of **N'** (40 mM) and **P** (10 mM) in toluene-*d*8 in the presence of 10 mM ***n*Bu$_3$PO.** On the right side of the figure is reported the time after the addition of **N'**.

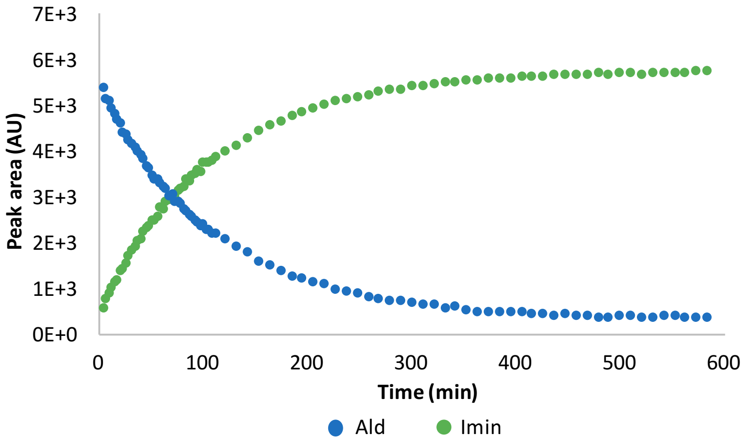

Figure S41. Kinetics of imine formation measured by integration of imine and aldehyde signals in the 500 MHz ^{1}H NMR spectra (**P** 10 mM, **N'** 40 mM, ***n*Bu$_3$PO** 10 mM). Aldehyde signals (**Ald**, 9,69 - 9,67 ppm) and imine (**Imin**, 8.27-8.25 ppm) signals are integrated and reported.

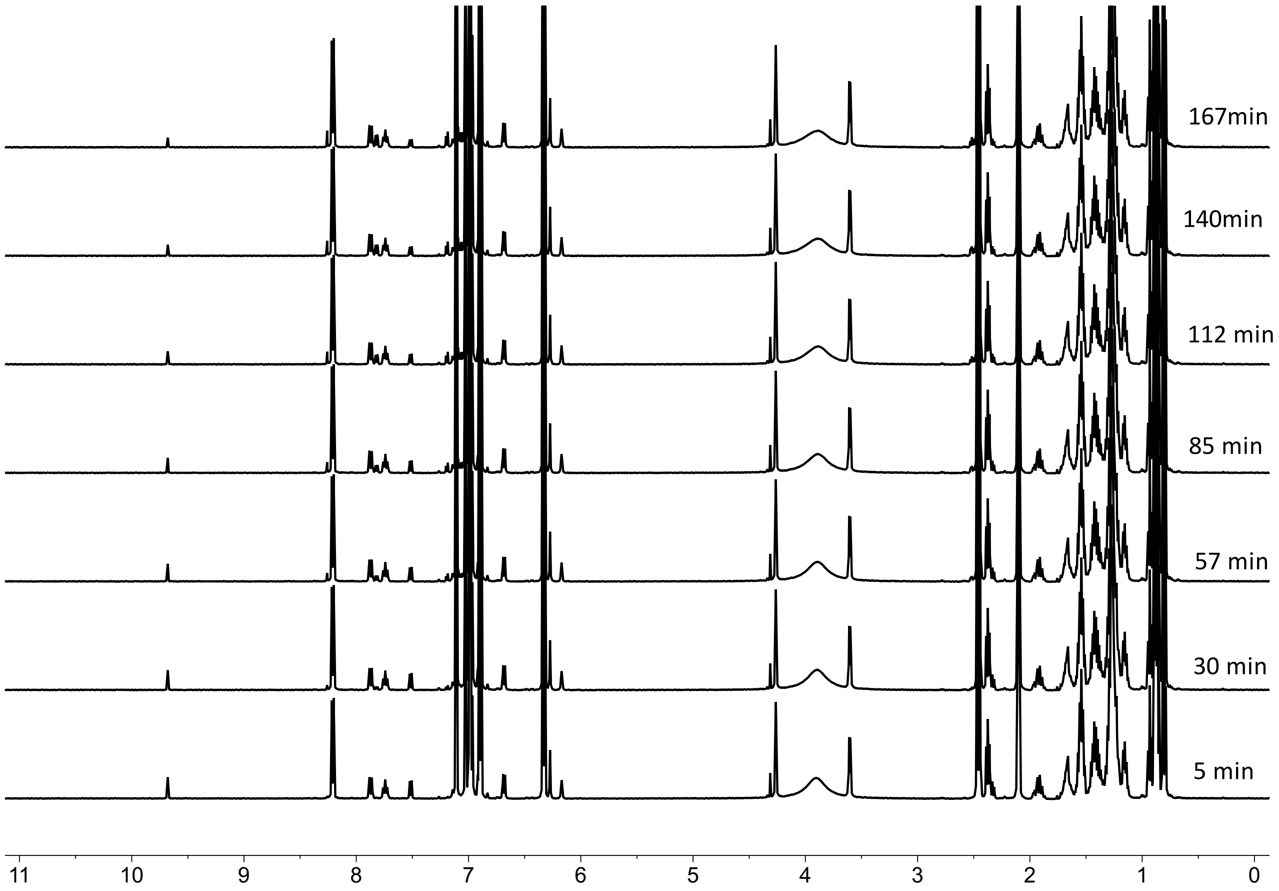

Figure S42. 500 MHz ^{1}H NMR spectra of a mixture of **N'** (40 mM) and **P** (10 mM) in toluene-*d*8 in the presence of 5 mM **AA.** On the right side of the figure is reported the time after the addition of **N'**.

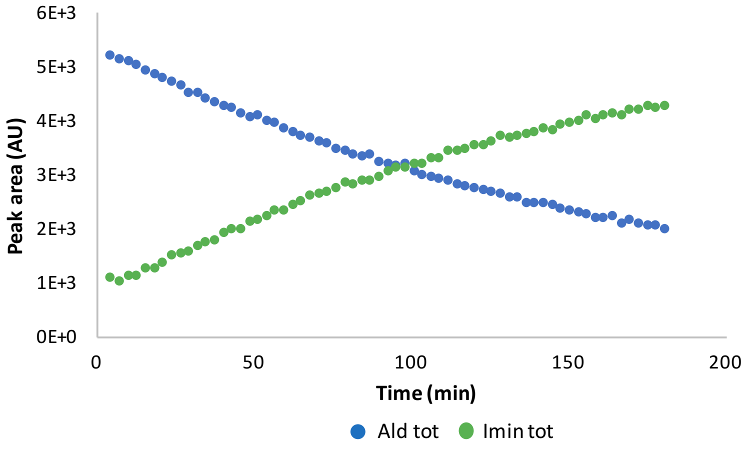

Figure S43. Kinetics of imine formation measured by integration of imine and aldehyde signals in the 500 MHz ^{1}H NMR spectra (**P** 10 mM, **N'** 40 mM, **AA** 5 mM). Aldehyde signals (**Ald**, 9,69 - 9,67 ppm) and imine (**Imin**, 8.27-8.25 ppm) signals are integrated and reported.

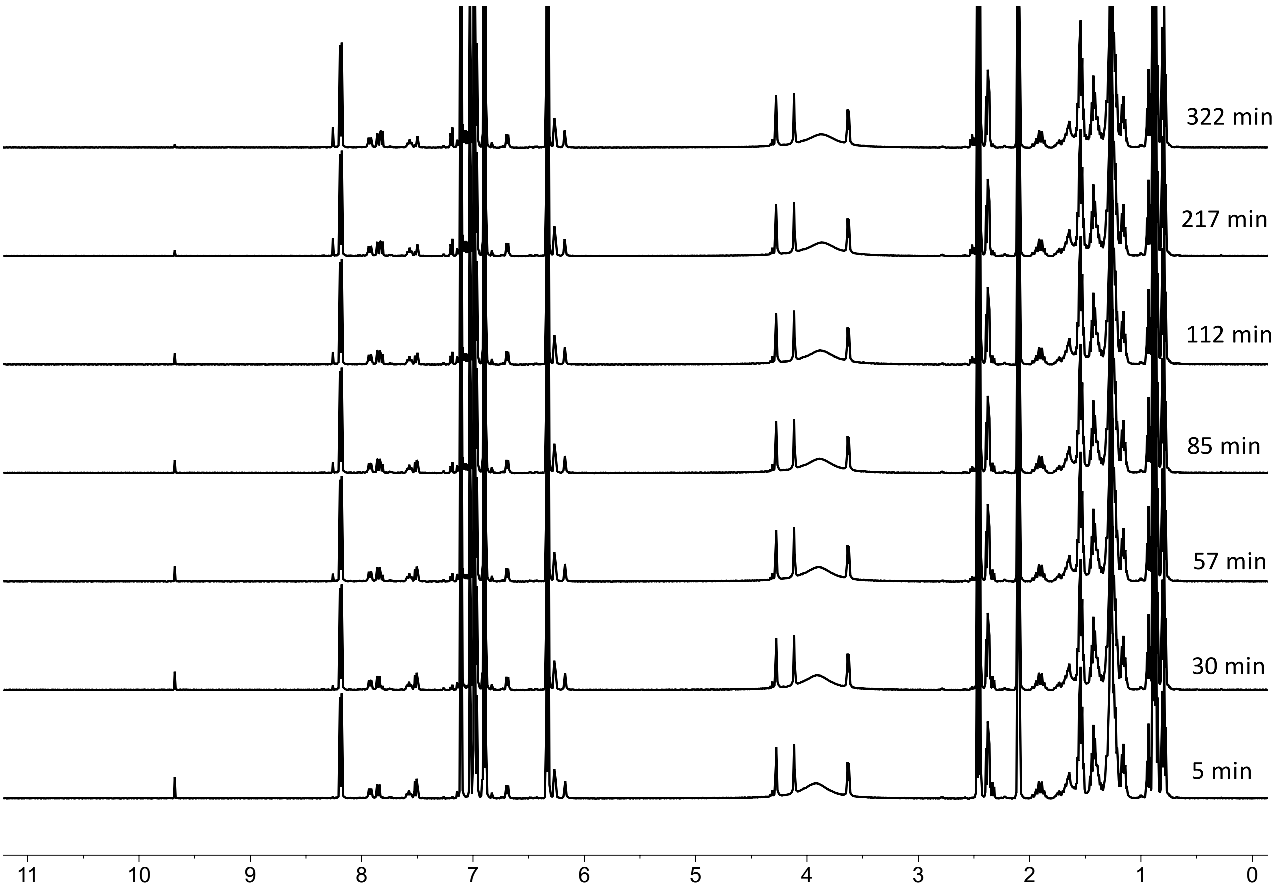

Figure S44. 500 MHz ^{1}H NMR spectra of a mixture of **N'** (40 mM) and **P** (10 mM) in toluene-*d*8 in the presence of 3.3 mM **AAA.** On the right side of the figure is reported the time after the addition of **N'**.

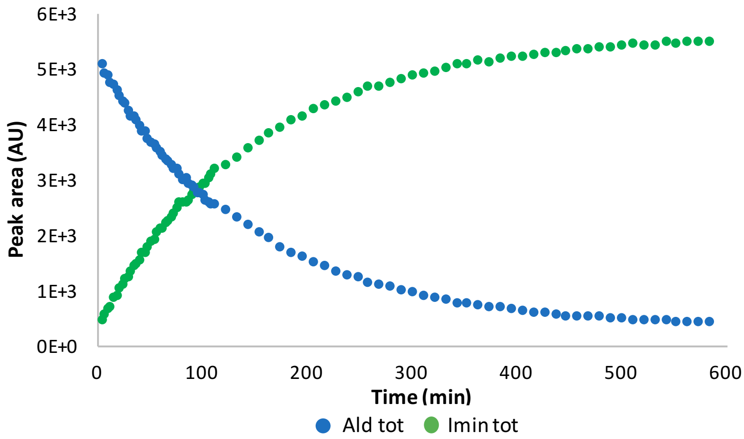

Figure S45. Kinetics of imine formation measured by integration of imine and aldehyde signals in the 500 MHz ^{1}H NMR spectra (**P** 10 mM, **N'** 40 mM, **AAA** 3.3 mM). Aldehyde signals (**Ald**, 9,69 - 9,67 ppm) and imine (**Imin**, 8.27-8.25 ppm) signals are integrated and reported.

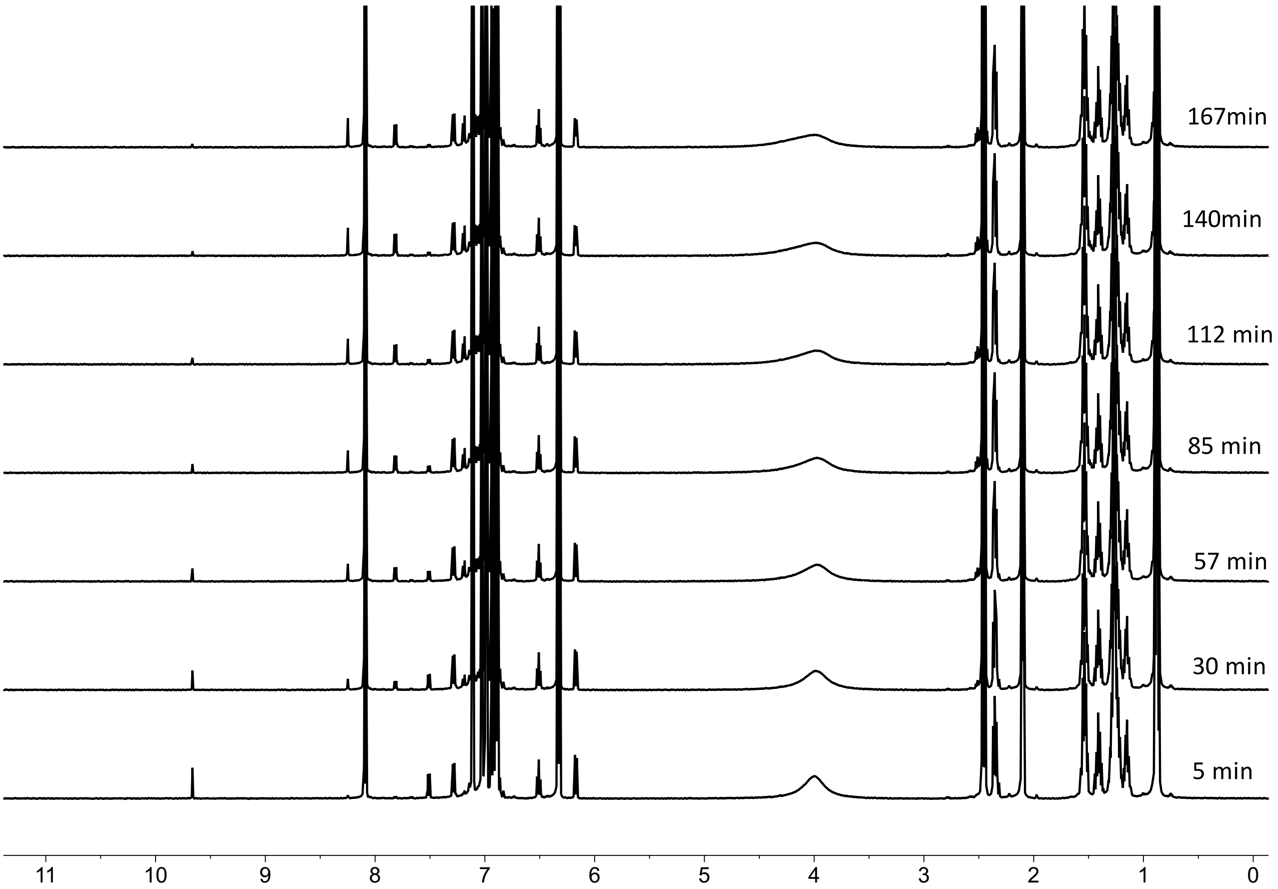

Figure S46. 500 MHz ^{1}H NMR spectra of a mixture of **N'** (40 mM) and **P** (10 mM) in toluene-*d*8 in the presence of 10 mM **CF_3PhOH.** On the right side of the figure is reported the time after the addition of **N'**.

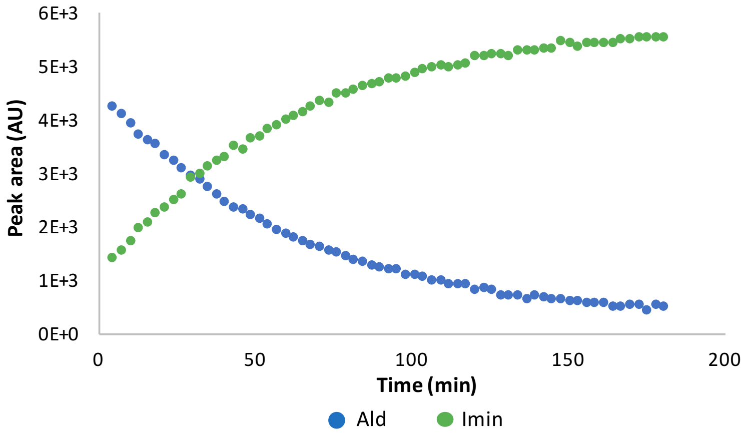

Figure S47. Kinetics of imine formation measured by integration of imine and aldehyde signals in the 500 MHz ^{1}H NMR spectra (**P** 10 mM, **N'** 40 mM, 10 mM **CF_3PhOH**). Aldehyde signals (**Ald**, 9,69 - 9,67 ppm) and imine (**Imin**, 8.27-8.25 ppm) signals are integrated and reported.

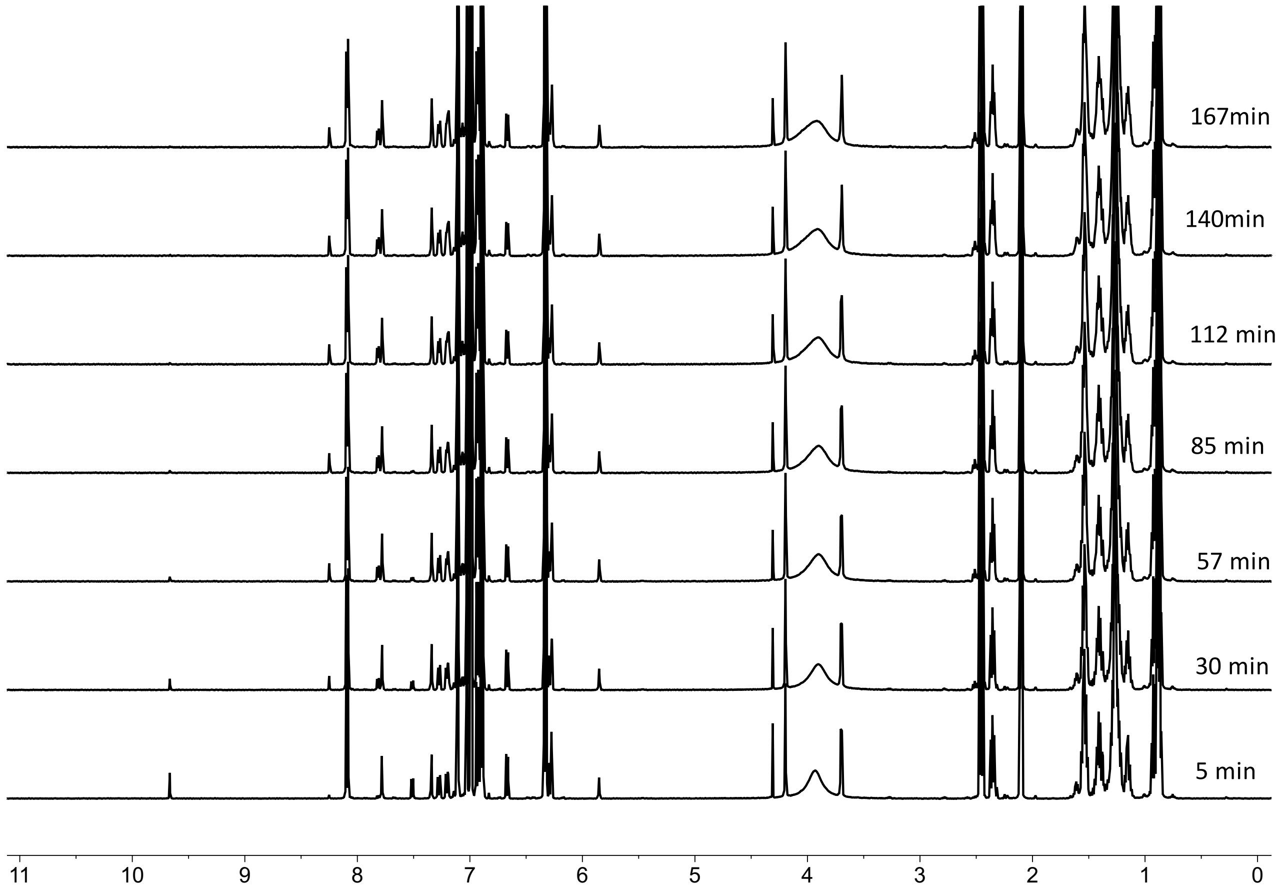

Figure S48. 500 MHz ^{1}H NMR spectra of a mixture of **N'** (40 mM) and **P** (10 mM) in toluene-*d*8 in the presence of 5 mM **DD.** On the right side of the figure is reported the time after the addition of **N'**.

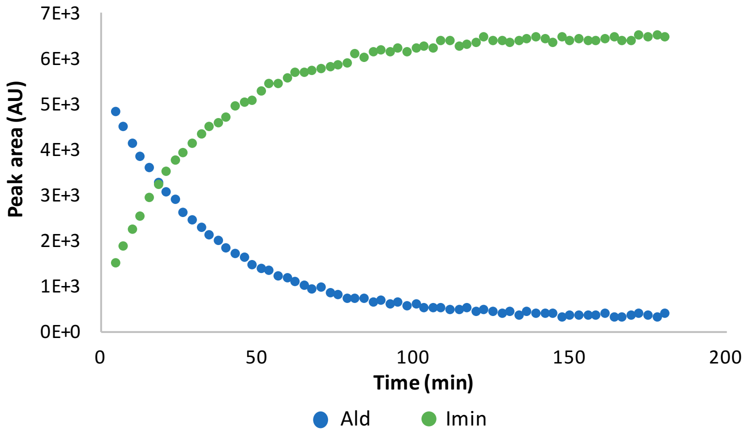

Figure S49. Kinetics of imine formation measured by integration of imine and aldehyde signals in the 500 MHz ^{1}H NMR spectra (**P** 10 mM, **N'** 40 mM, 5 mM **DD**). Aldehyde signals (**Ald**, 9,69 - 9,67 ppm) and imine (**Imin**, 8.27-8.25 ppm) signals are integrated and reported.

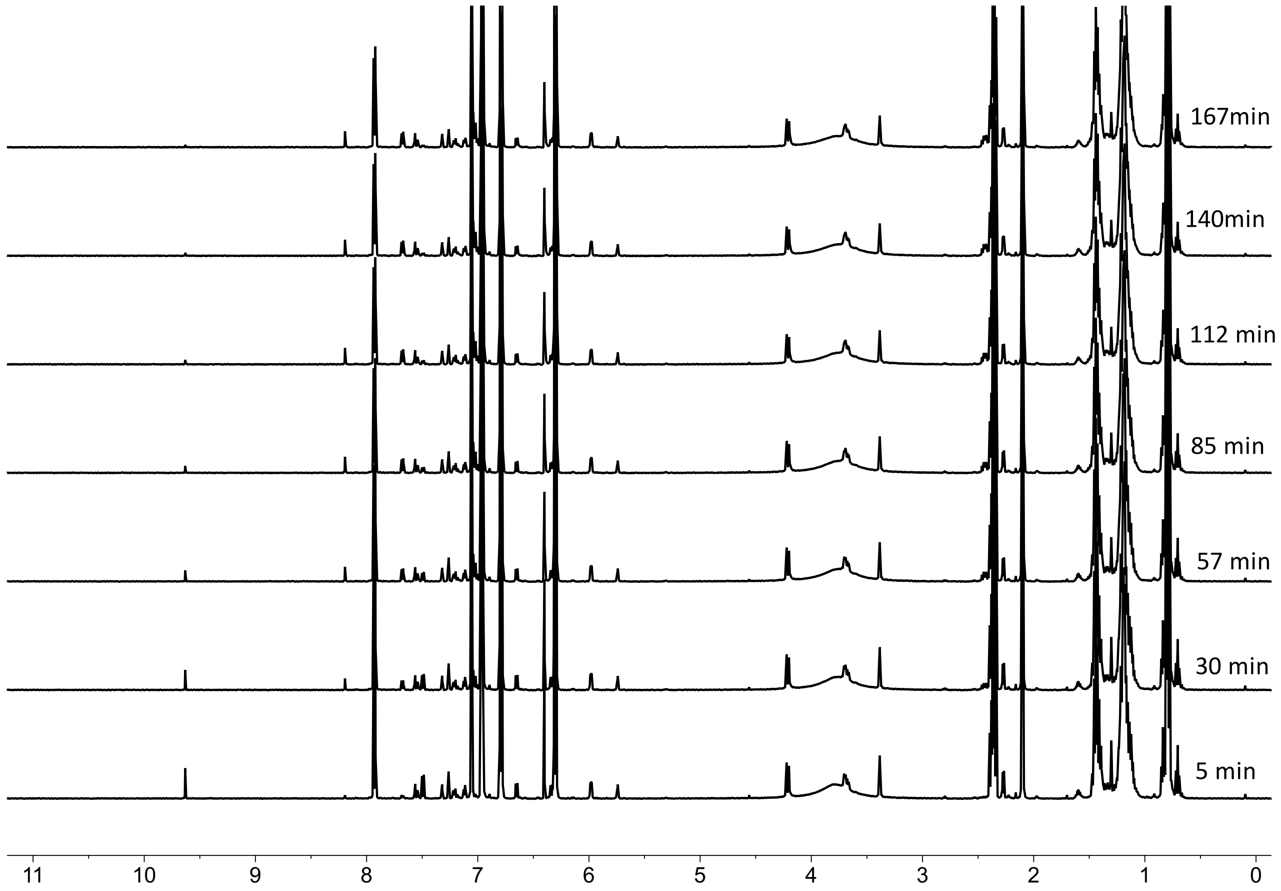

Figure S50. 500 MHz ^{1}H NMR spectra of a mixture of **N'** (40 mM) and **P** (10 mM) in toluene-*d*8 in the presence of 3.3 mM **DDD.** On the right side of the figure is reported the time after the addition of **N'**.

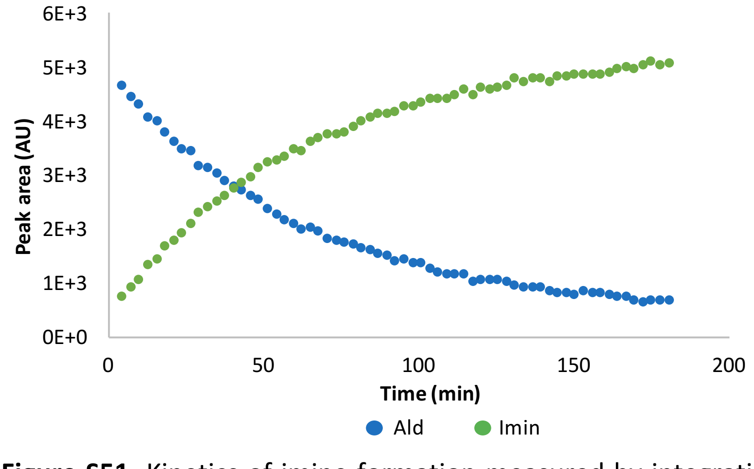

Figure S51. Kinetics of imine formation measured by integration of imine and aldehyde signals in the 500 MHz ^{1}H NMR spectra (**P** 10 mM, **N'** 40 mM, 3.3 mM **DDD**). Aldehyde signals (**Ald**, 9,69 - 9,67 ppm) and imine (**Imin**, 8.27-8.25 ppm) signals are integrated and reported.

a)

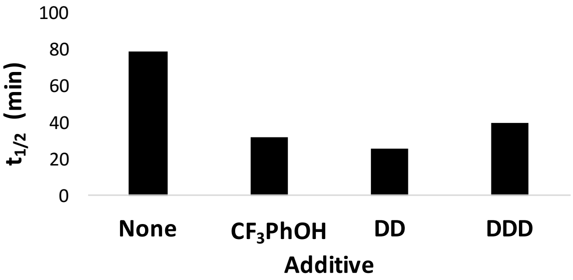

b)

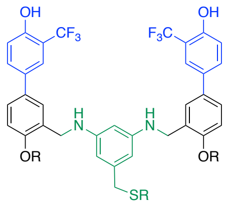

Figure S52. Effects of donor oligomers on the rate of imine formation in a system with no recognition units. **a)** Half-life of the aldehyde starting material measured by integration of 500 MHz ^{1}H-NMR signals as a function of time. Reaction conditions: 20 mM aldehyde **P**, 40 mM aniline **N'** and 10 mM phenol in toluene-*d*8 (i.e. 10 mM 2-trifluoromethyl phenol, 5 mM **DD**, or 3.3 mM **DDD**). **b)** Chemical structure of **DD**.

Figure S52 shows the control experiment to establish the effect of phenol oligomers on the rate of imine formation between an aldehyde and an aniline that do not have any recognition sites (**P** and **N'**). The presence of the phenol groups increases the reaction rate by a factor of 2-3, but there is no effect of oligomer length, and the half-lives in the presence of 2-trifluoromethyl phenol, **DD** and **DDD** are very similar.

2.9 ^{1}H-NMR Kinetic experiments: A-N

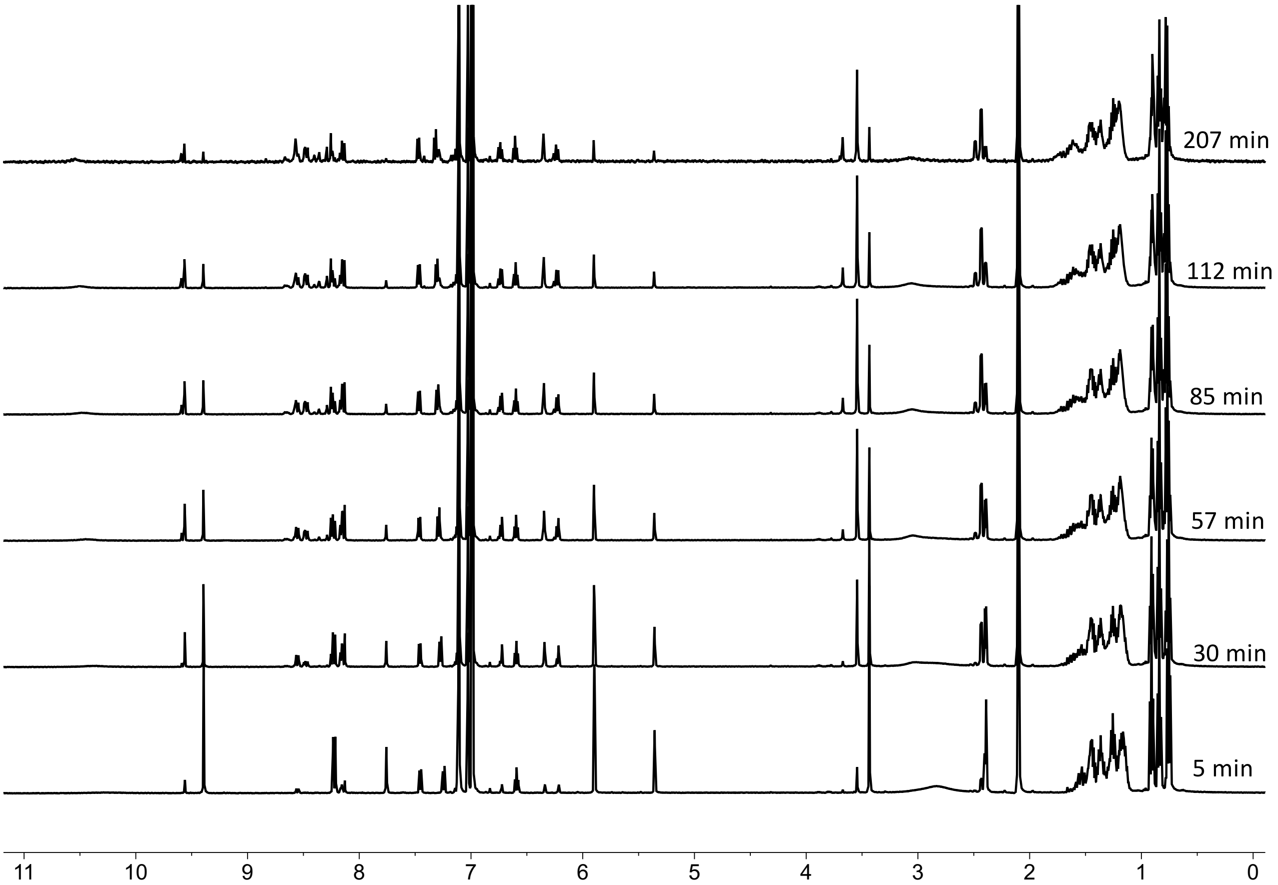

Figure S53. 500 MHz ^{1}H NMR spectra of a mixture of **N** (20 mM) and **A** (10 mM) in toluene-*d*8 in the presence of 10 mM **CF_3PhOH.** On the right side of the figure is reported the time after the addition of **N'**.

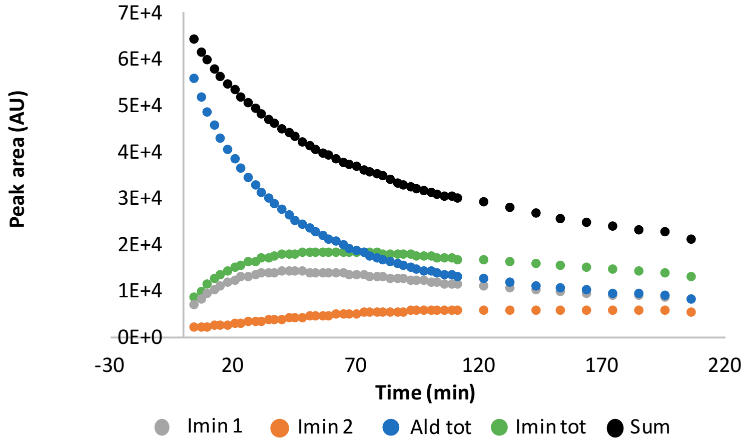

Figure S54. Kinetics of imine formation measured by integration of all imine and aldehyde signals in the 500 MHz ^{1}H NMR spectra (**A** 10 mM, **N** 20 mM, **CF_3PhOH** 10 mM). Aldehyde signals (**Ald tot**, 9.43-9.35 ppm), monoimine (**Imin 1**, 9.58-9.55 ppm), bis imine (**Imin 2**, 9.61-9.58 ppm) and total imine (**Imin tot**, 9.62-9.55 ppm) signals are integrated and reported. Sum of signals is decreasing during the time, because of the imines precipitation.

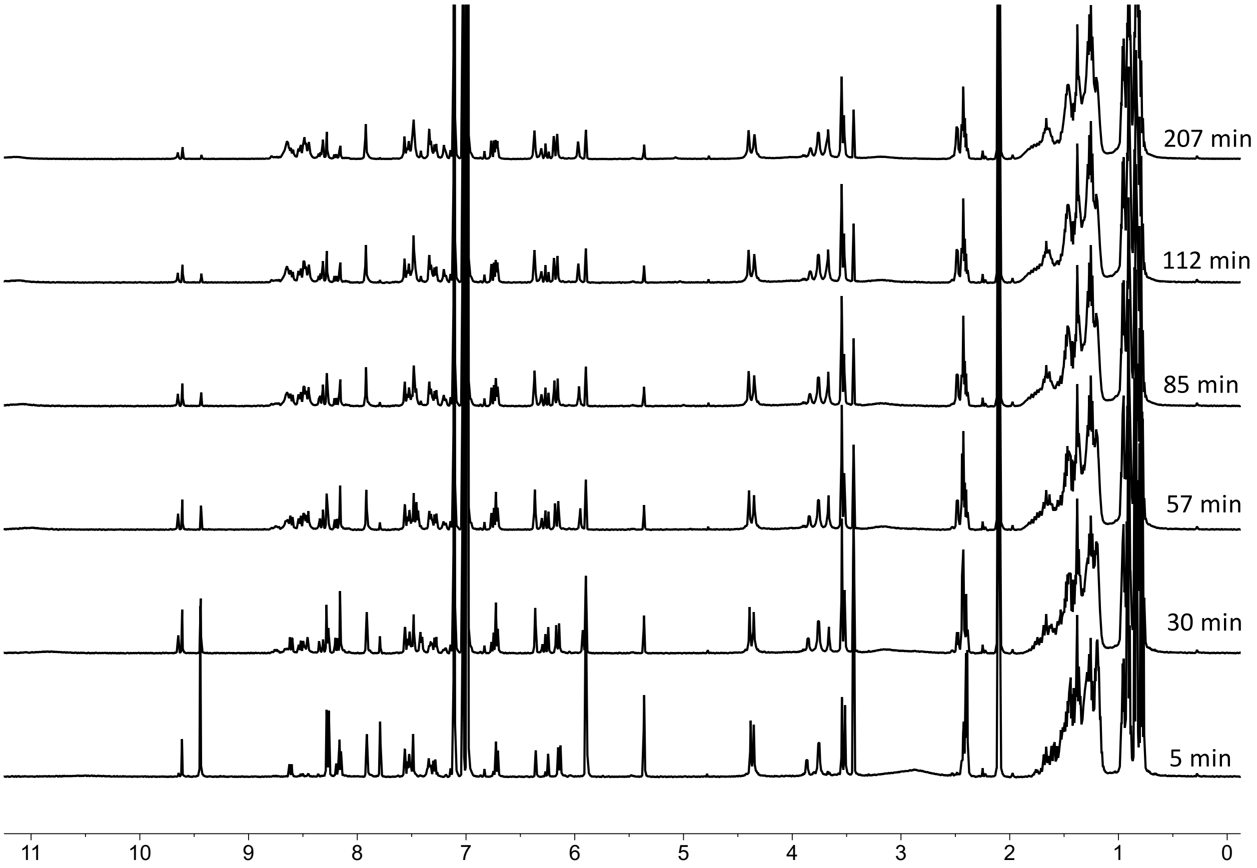

Figure S55. 500 MHz ^{1}H NMR spectra of a mixture of **N** (20 mM) and **A** (10 mM) in toluene-*d*8 in the presence of 3.3 mM **DDD.** On the right side of the figure is reported the time after the addition of **N'**.

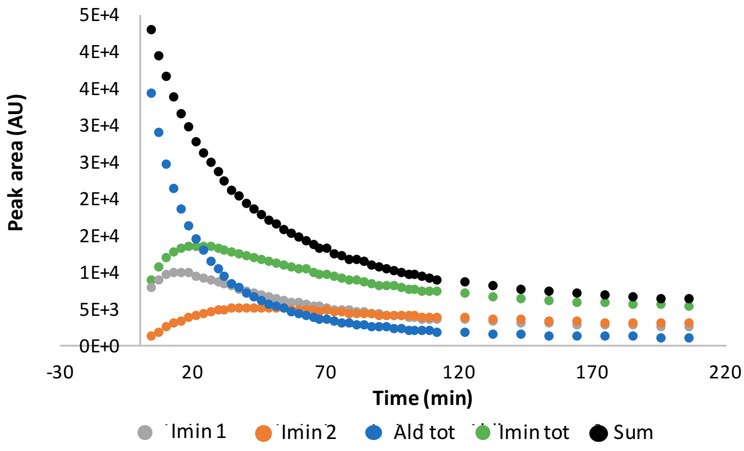

Figure S56. Kinetics of imine formation measured by integration of all imine and aldehyde signals in the 500 MHz ^{1}H NMR spectra (**A** 10 mM, **N** 20 mM, **DDD** 3.3 mM). Aldehyde signals (**Ald tot**, 9.43-9.35 ppm), monoimine (**Imin 1**, 9.58-9.55 ppm), bis imine (**Imin 2**, 9.61-9.58 ppm) and total imine (**Imin tot**, 9.62-9.55 ppm) signals are integrated and reported. Sum of signals is decreasing during the time, because of the imines precipitation.

2.10 ^{1}H-NMR Kinetic experiments: A'-N

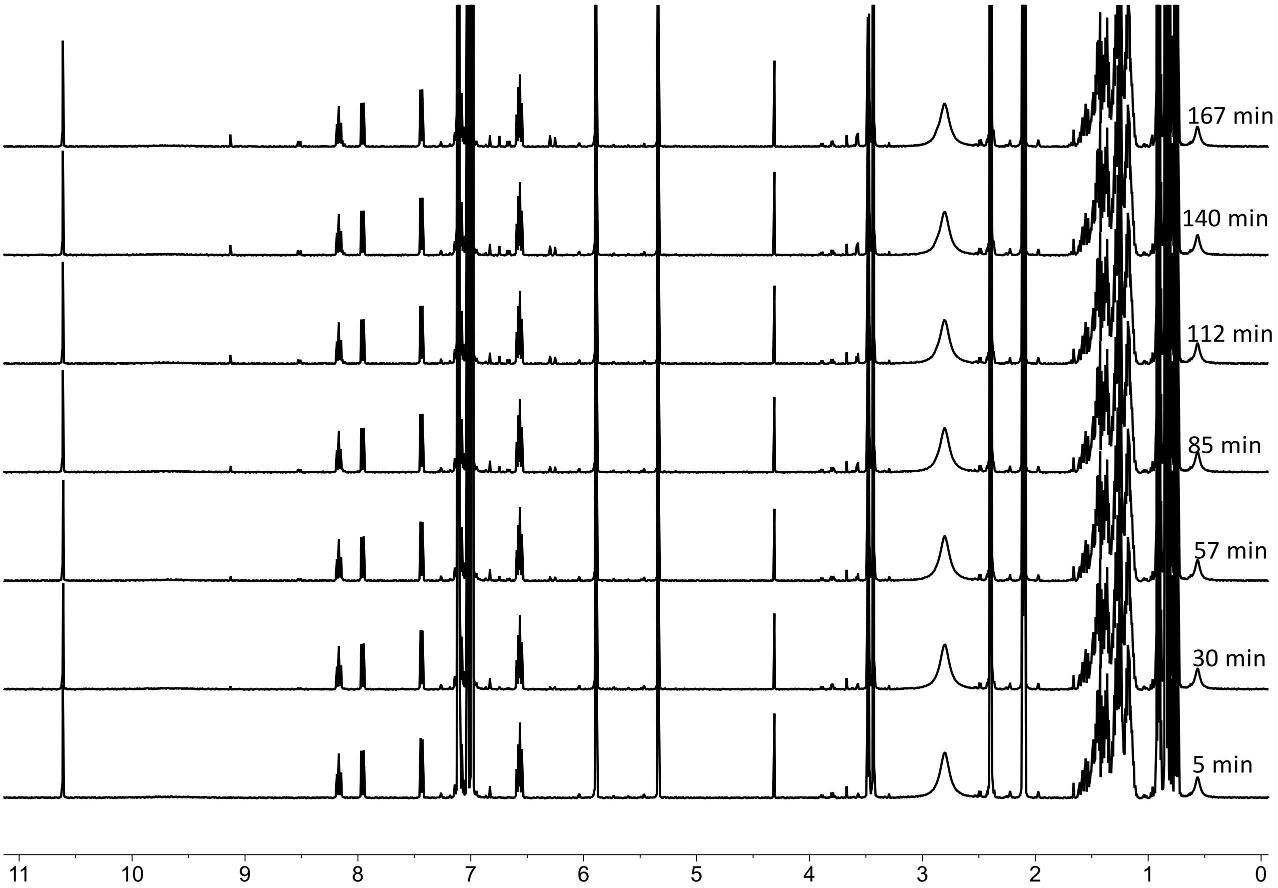

Figure S57. 500 MHz ^{1}H NMR spectra of a mixture of **N** (20 mM) and **A'** (10 mM) in toluene-*d*8 in the presence of 10 mM **CF_3PhOH.** On the right side of the figure is reported the time after the addition of **N'**.

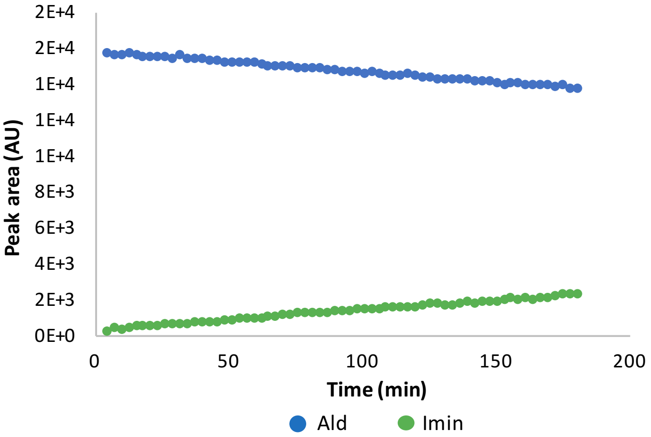

Figure S58. Kinetics of imine formation measured by integration of all imine and aldehyde signals in the 500 MHz ^{1}H NMR spectra (**A'** 10 mM, **N** 20 mM, **CF_3PhOH** 10 mM). Aldehyde (**Ald**, 10.64-10,59 ppm), and total imine (**Imin**, 9.15-9.10 ppm) signals are integrated and reported.

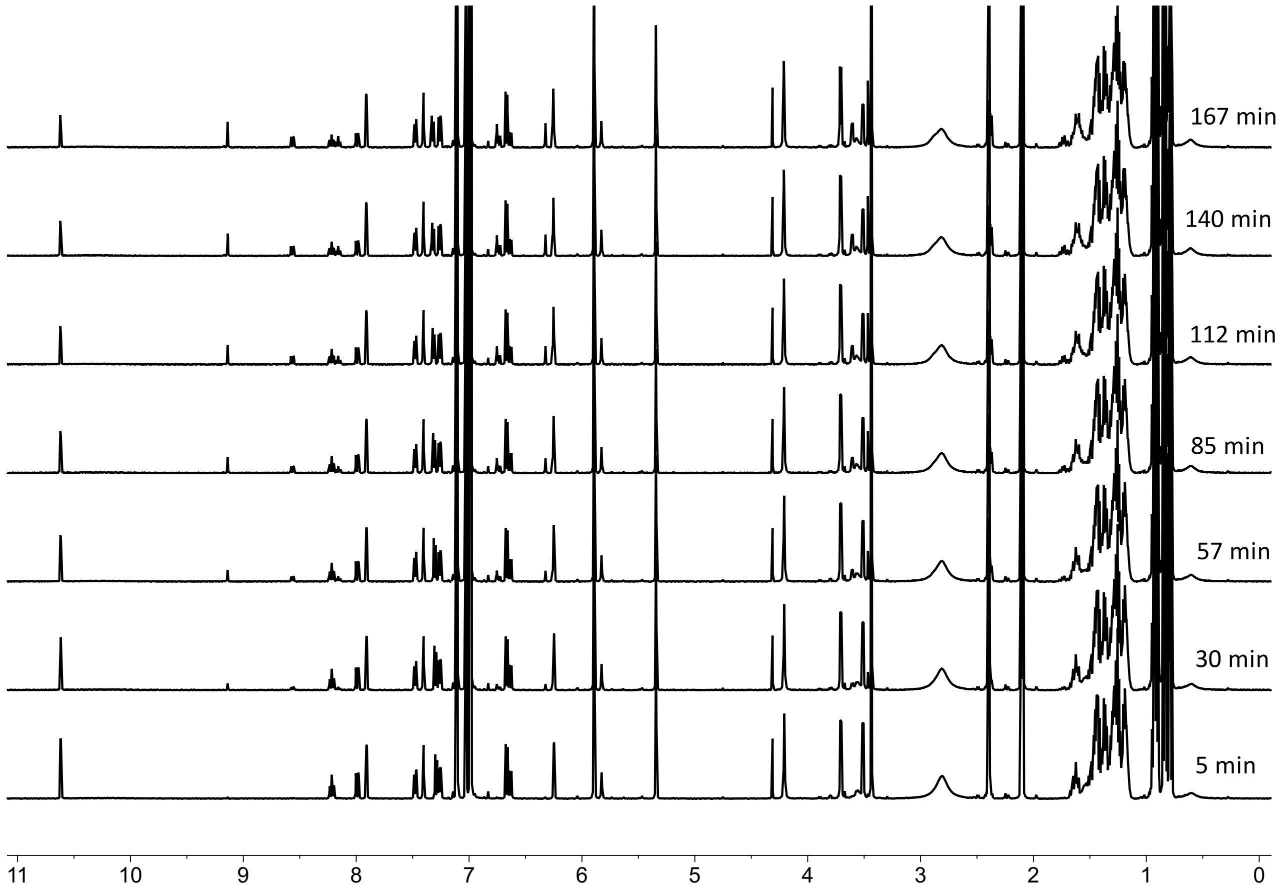

Figure S59. 500 MHz ^{1}H NMR spectra of a mixture of **N** (20 mM) and **A'** (10 mM) in toluene-*d*8 in the presence of 5 mM **DD.** On the right side of the figure is reported the time after the addition of **N'**.

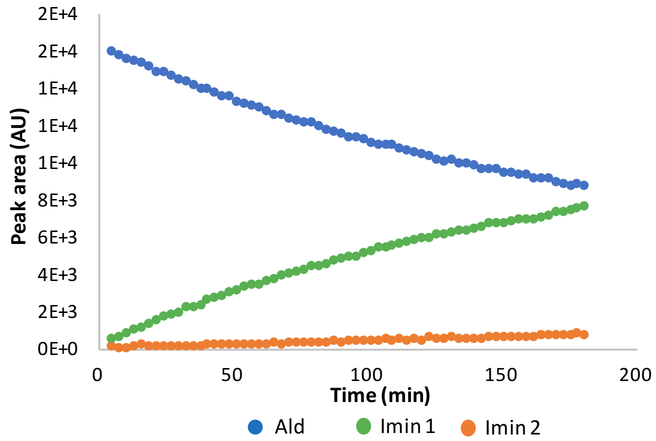

Figure S60. Kinetics of imine formation measured by integration of all imine and aldehyde signals in the 500 MHz ^{1}H NMR spectra (**A'** 10 mM, **N** 20 mM, **DD** 5 mM). Aldehyde (**Ald**, 10.64-10,59 ppm), monomeric imine (**Imin 1**, 9.16-9.11ppm) and dimeric imine (**Imin 2**, 9.18-9.16 ppm) signals are integrated and reported.

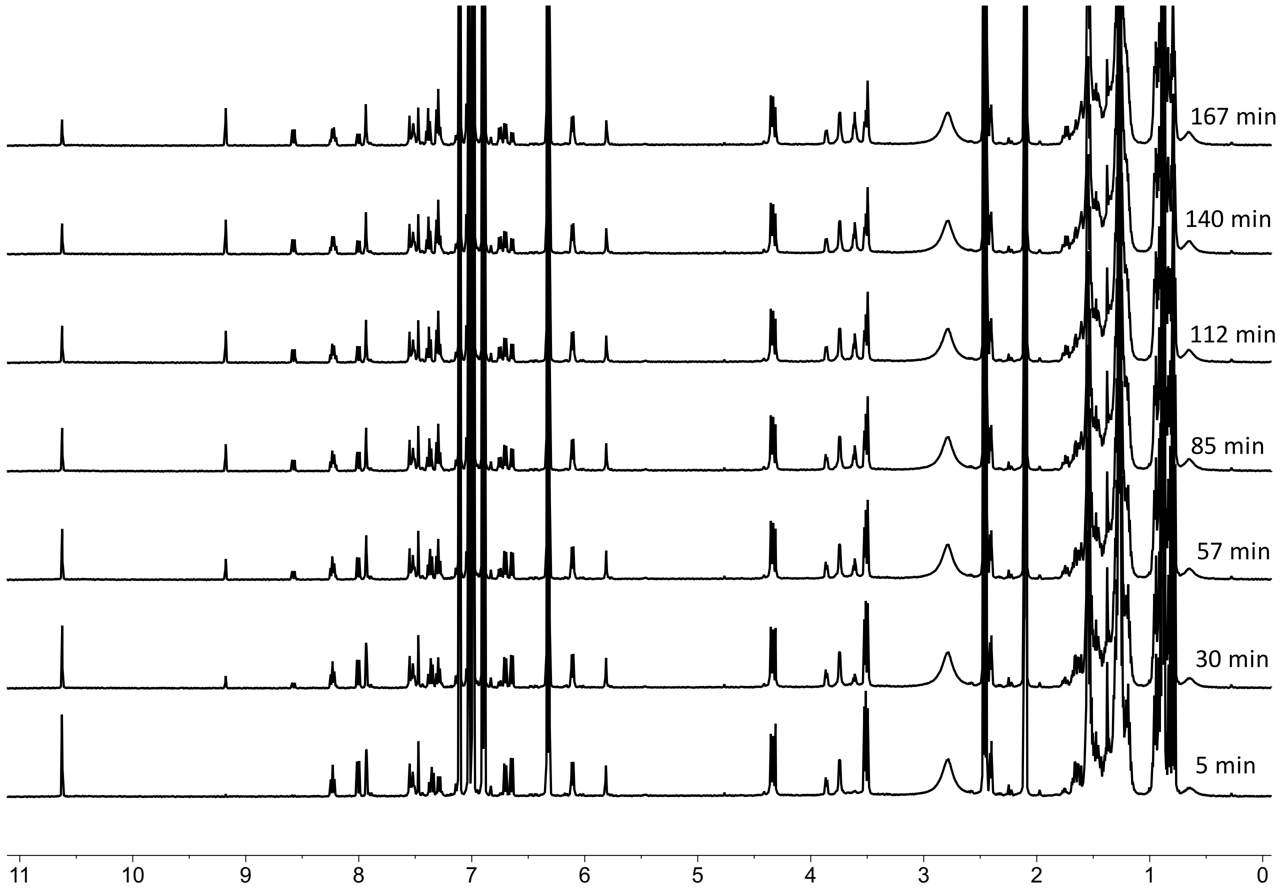

Figure S61. 500 MHz ^{1}H NMR spectra of a mixture of **N** (20 mM) and **A'** (10 mM) in toluene-*d*8 in the presence of 3.3 mM **DDD.** On the right side of the figure is reported the time after the addition of **N'**.

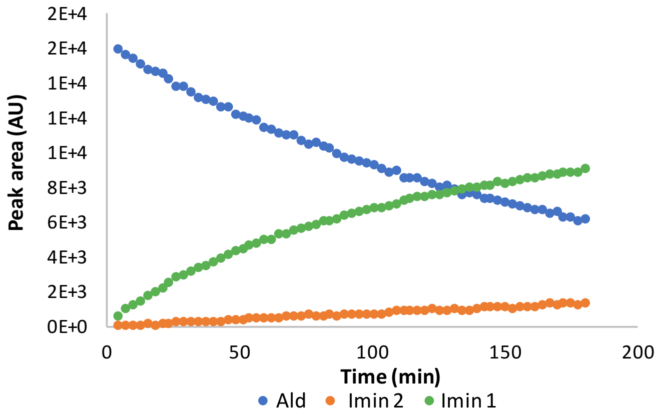

Figure S62. Kinetics of imine formation measured by integration of all imine and aldehyde signals in the 500 MHz ^{1}H NMR spectra (**A'** 10 mM, **N** 20 mM, **DDD** 3.3 mM). Aldehyde (**Ald**, 10.64-10,59 ppm), monomeric imine (**Imin 1**, 9.16-9.11 ppm) and dimeric imine (**Imin 2**, 9.18-9.16 ppm) signals are integrated and reported.

2.11 ^{1}H-NMR Kinetic experiments: A'-N'

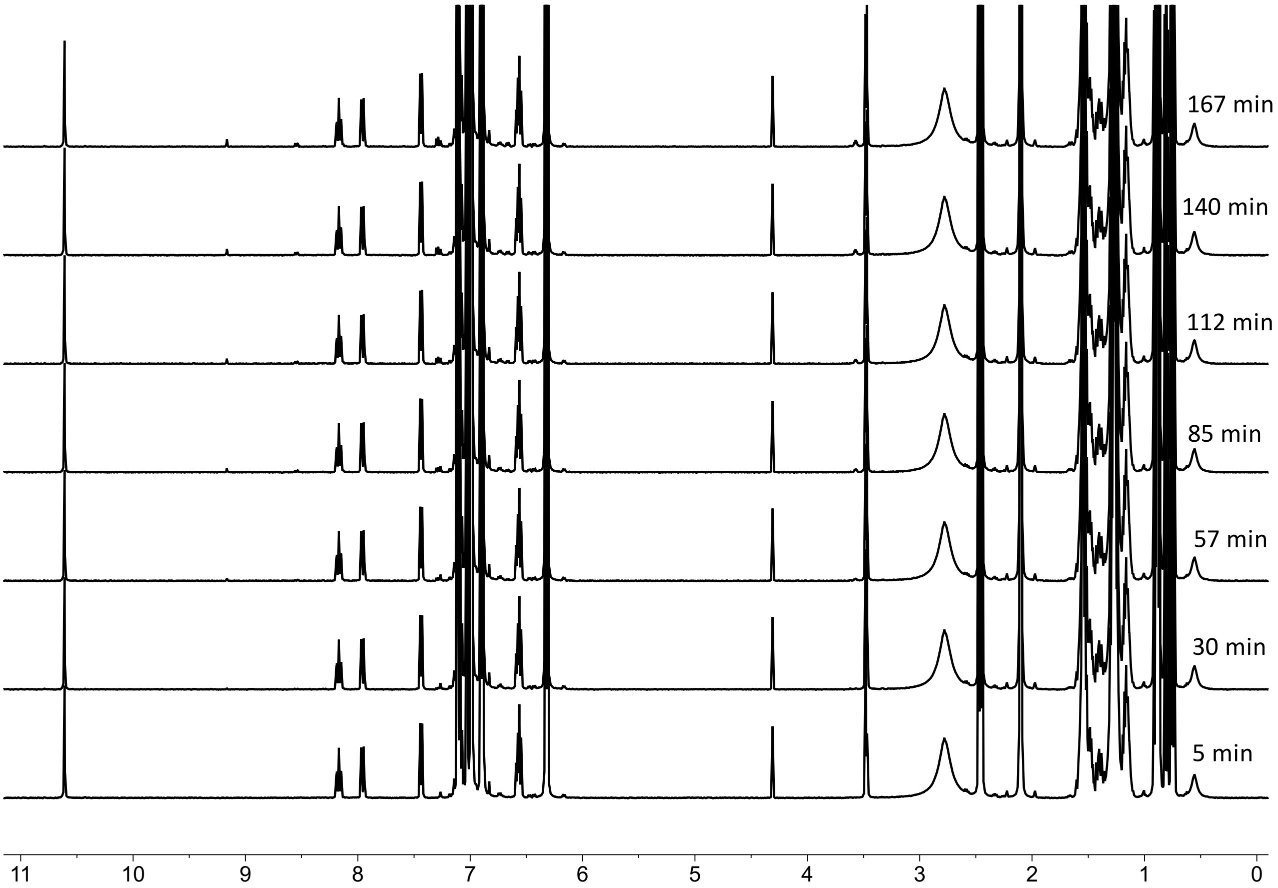

Figure S63. 500 MHz ^{1}H NMR spectra of a mixture of **N'** (40 mM) and **A'** (10 mM) in toluene-*d*8 in the presence of 10 mM **CF_3PhOH.** On the right side of the figure is reported the time after the addition of **N'**.

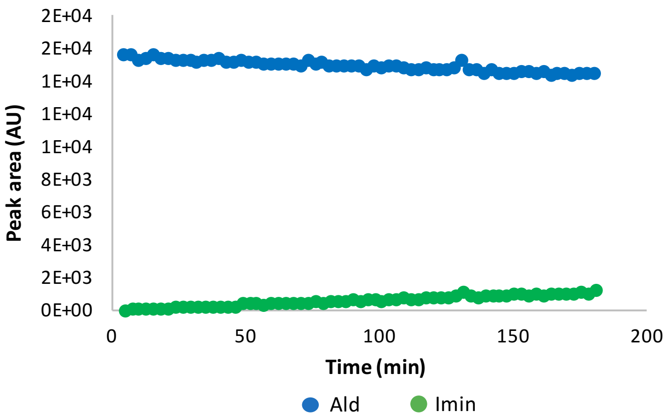

Figure S64. Kinetics of imine formation measured by integration of all imine and aldehyde signals in the 500 MHz ^{1}H NMR spectra (**A'** 10 mM, **N'** 40 mM, **CF_3PhOH** 10 mM). Aldehyde (**Ald**, 10.65-10,57 ppm), and total imine (**Imin**, 9.19-9.14 ppm) signals are integrated and reported.

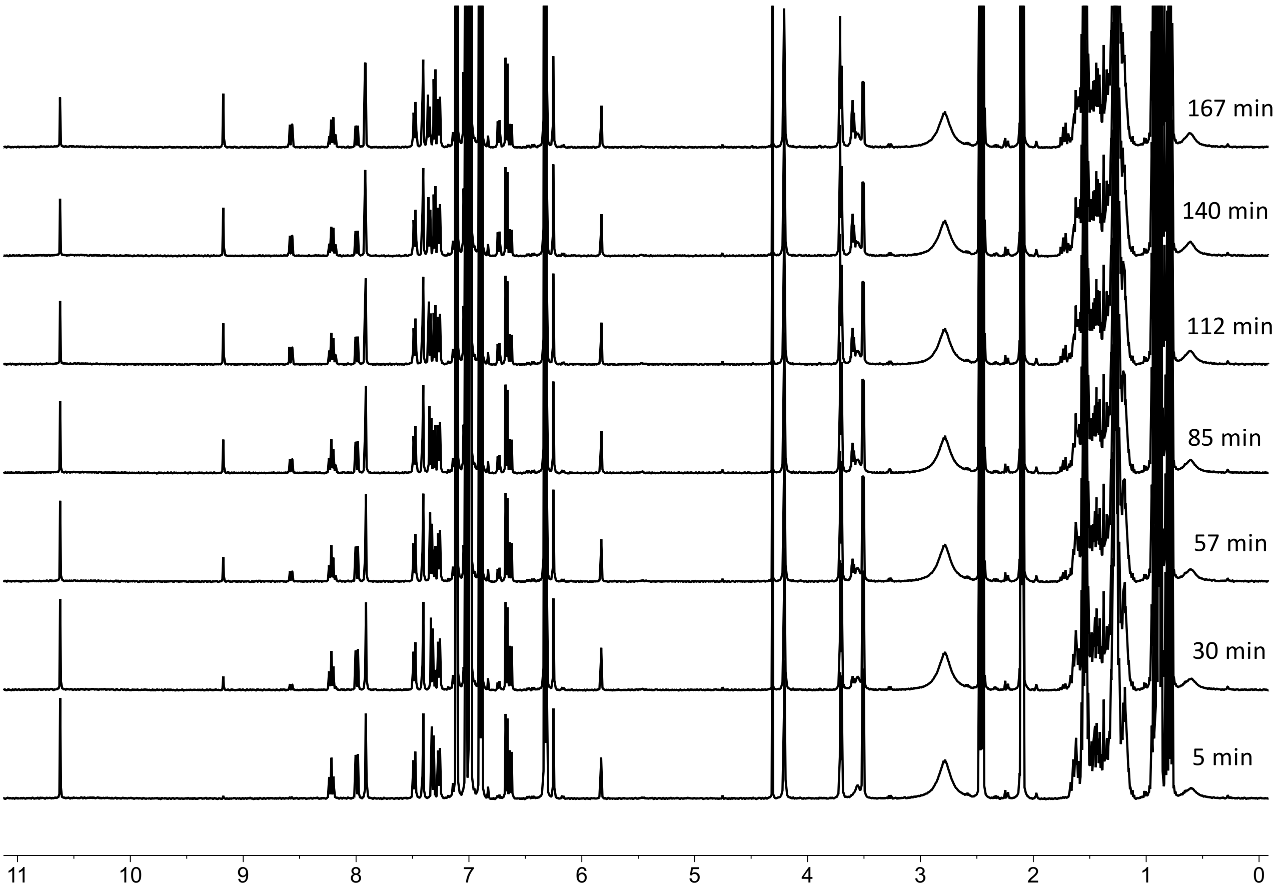

Figure S65. 500 MHz ^{1}H NMR spectra of a mixture of **N'** (40 mM) and **A'** (10 mM) in toluene-*d*8 in the presence of 5 mM **DD.** On the right side of the figure is reported the time after the addition of **N'**.

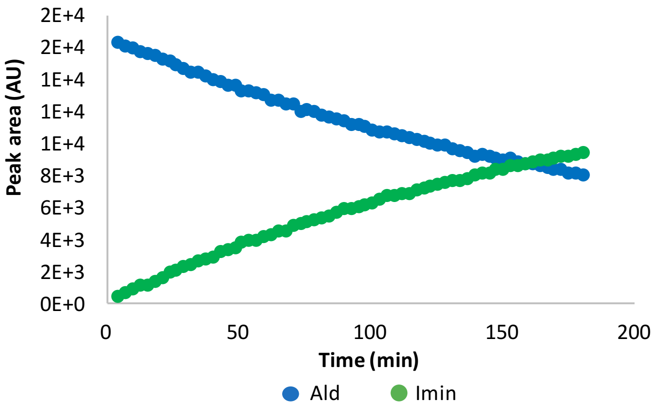

Figure S66. Kinetics of imine formation measured by integration of all imine and aldehyde signals in the 500 MHz ^{1}H NMR spectra (**A'** 10 mM, **N'** 40 mM, **DD** 5 mM). Aldehyde (**Ald**, 10.65-10,57 ppm), and total imine (**Imin**, 9.19-9.14 ppm) signals are integrated and reported.

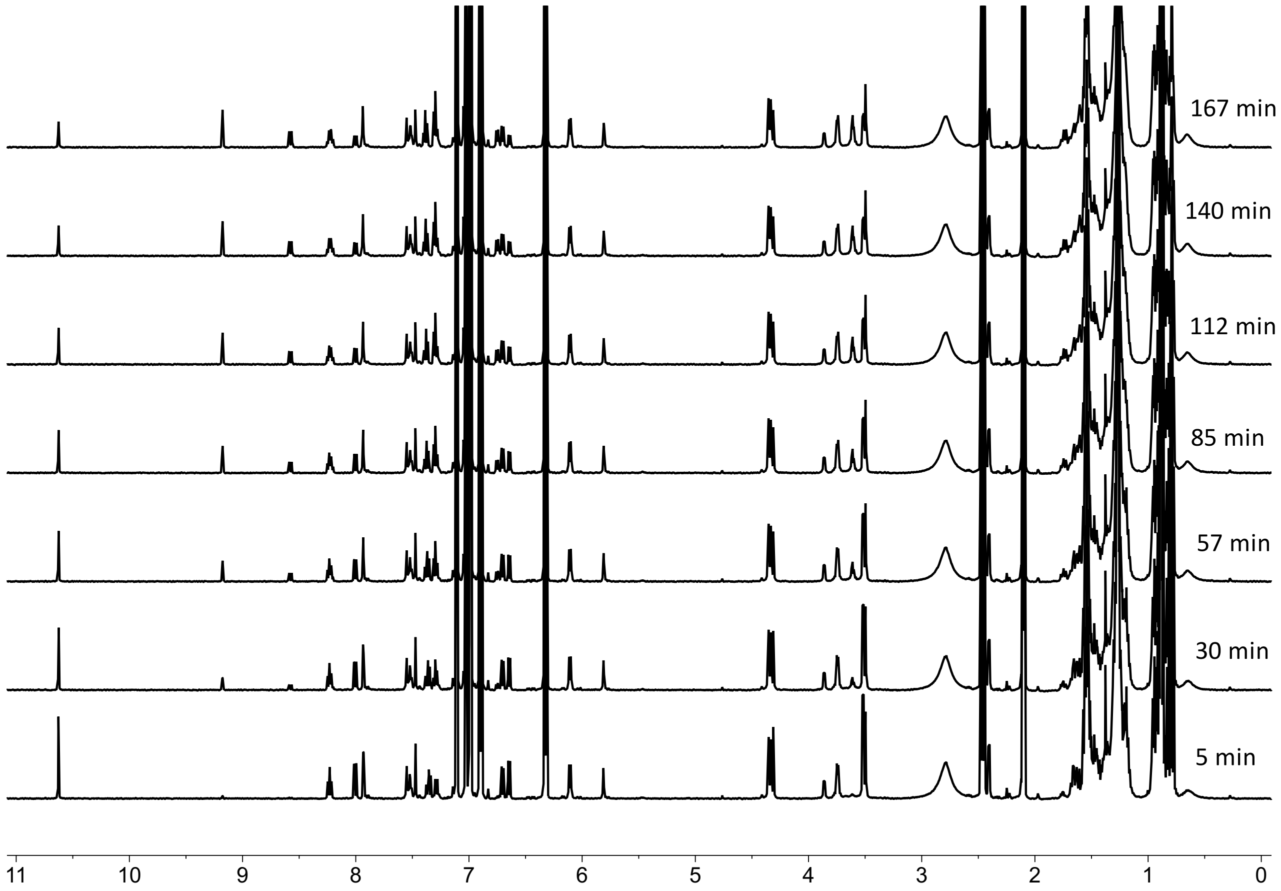

Figure S67. 500 MHz ^{1}H NMR spectra of a mixture of **N'** (40 mM) and **A'** (10 mM) in toluene-*d*8 in the presence of 3.3 mM **DDD.** On the right side of the figure is reported the time after the addition of **N'**.

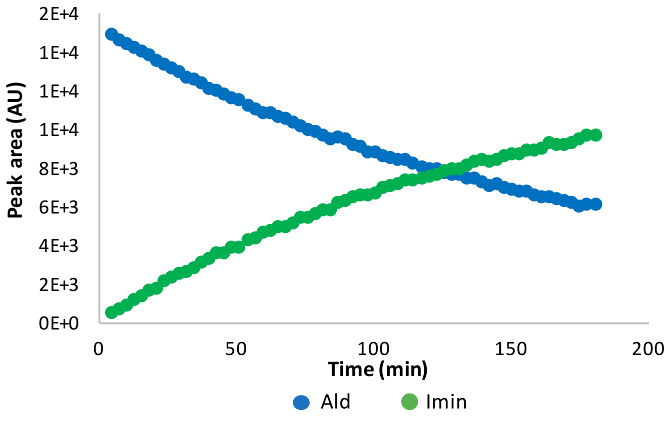

Figure S68. Kinetics of imine formation measured by integration of all imine and aldehyde signals in the 500 MHz ^{1}H NMR spectra (**A'** 10 mM, **N'** 40 mM, **DDD** 3.3 mM). Aldehyde (**Ald**, 10.65-10,57 ppm), and total imine (**Imin**, 9.19-9.14 ppm) signals are integrated and reported.

2.12 ^{1}H-NMR Kinetic experiments: AAA selectivity

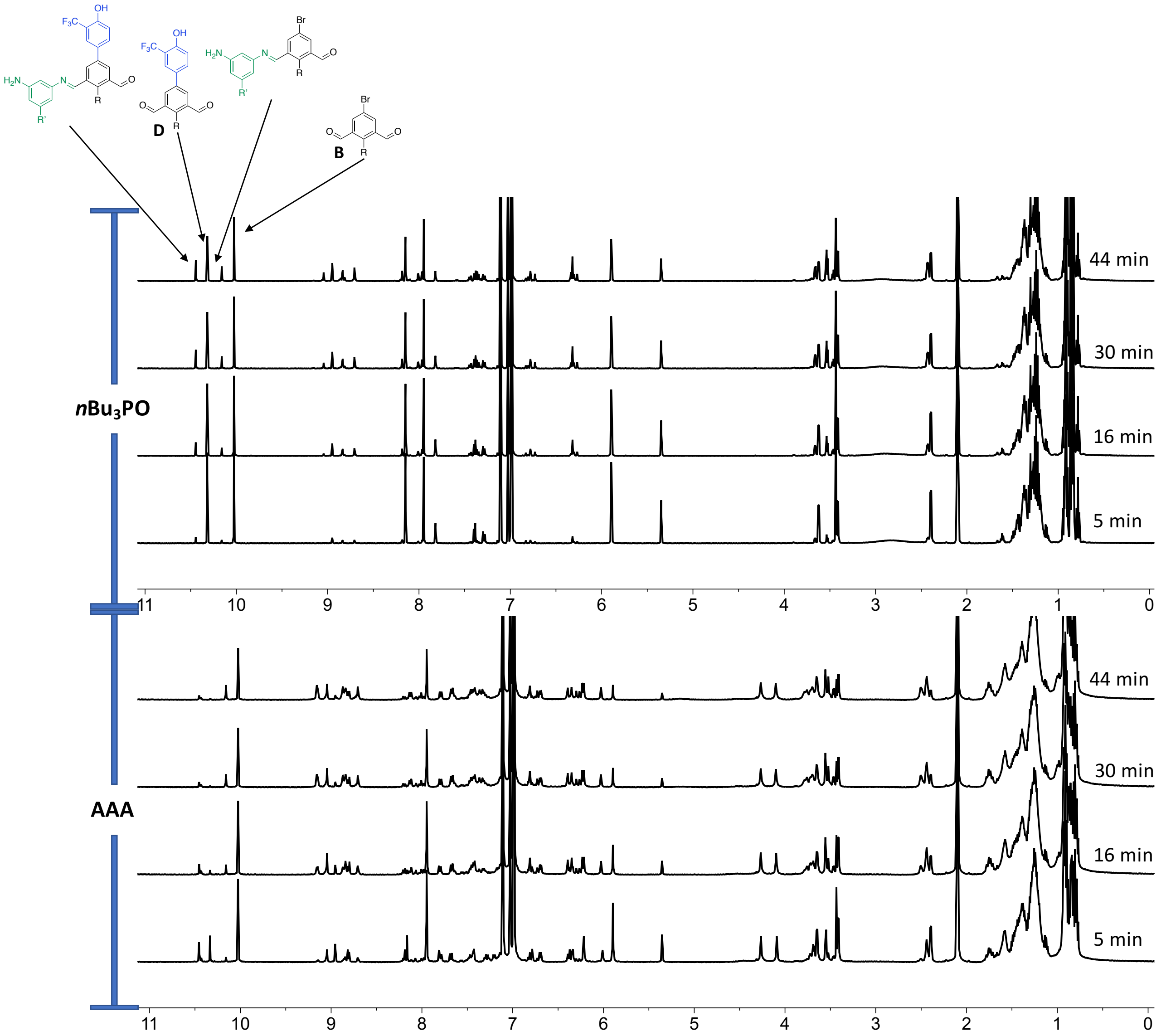

Figure S69. 500 MHz ^{1}H-NMR spectra of a mixture of **D** (10 mM), **B** (10 mM) and **N** (20 mM) in toluene-*d*8 and in the presence of (bottom) **AAA** (3.3 mM) or (top) *n*Bu$_3$PO (10 mM). The signals at 10.3-10.5 ppm are due to the aldehyde protons of **D** monomers and oligomers, the signal at 10.0-10.2 ppm is due to the aldehyde proton of **B**. On the right side of the figure is reported the time after the addition of **N**.

3. HPLC-MS studies

The chemical composition of the imine libraries was analysed by reverse phase HPLC using an Agilent 1200 Series composed of a HPLC high pressure binary pump, autosampler, column oven, a Diode Array Detector and an Agilent XCT ion trap mass spectrometer. 2.2 μm Coagent 2o Phenyl Hydride 3cmx3mm was used as the HPLC column. The conditions of the HPLC method are as follows:

Solvent **A**: 60% NH_4OAc 10 mM, pH 5.70, 40% THF;
Solvent **B**: 85% THF, 10% IPA, 5% Solvent A.
45% B for 1 min, then from 45% to 65% in 3.5 min and to 80% in 2 min. Flowrate 0.4 ml/min, Injection volume 0.1 uL, with needle wash before injection. Column temperature 45°C.

HPLC samples were prepared using Jaytee vials screw clear 1.5 mL, using 0.2 mL (31x6 mm) VWR micro insert. For every experiment the last addition was a toluene solution of the required aniline, then the vial was closed, vigorously shaken and then immediately injected.

Imine hydrolysis on the HPLC column is minimal, as evidenced by the following observations:
- imines are directly observed by mass spec of the HPLC peaks
- comparison of Figures S6 and S79 shows that the rate of imine formation measured by NMR is similar to that measured by HPLC
- the HPLC data in Figure 5 of the main text shows some aldehyde peaks, because it was recorded after 5 minutes, when imine formation is not complete. The HPLC data recorded after an hour in Figure S79 shows almost no aldehyde end groups, similar to what was observed directly by NMR.

3.1 HPLC-MS experiments: peaks characterisation

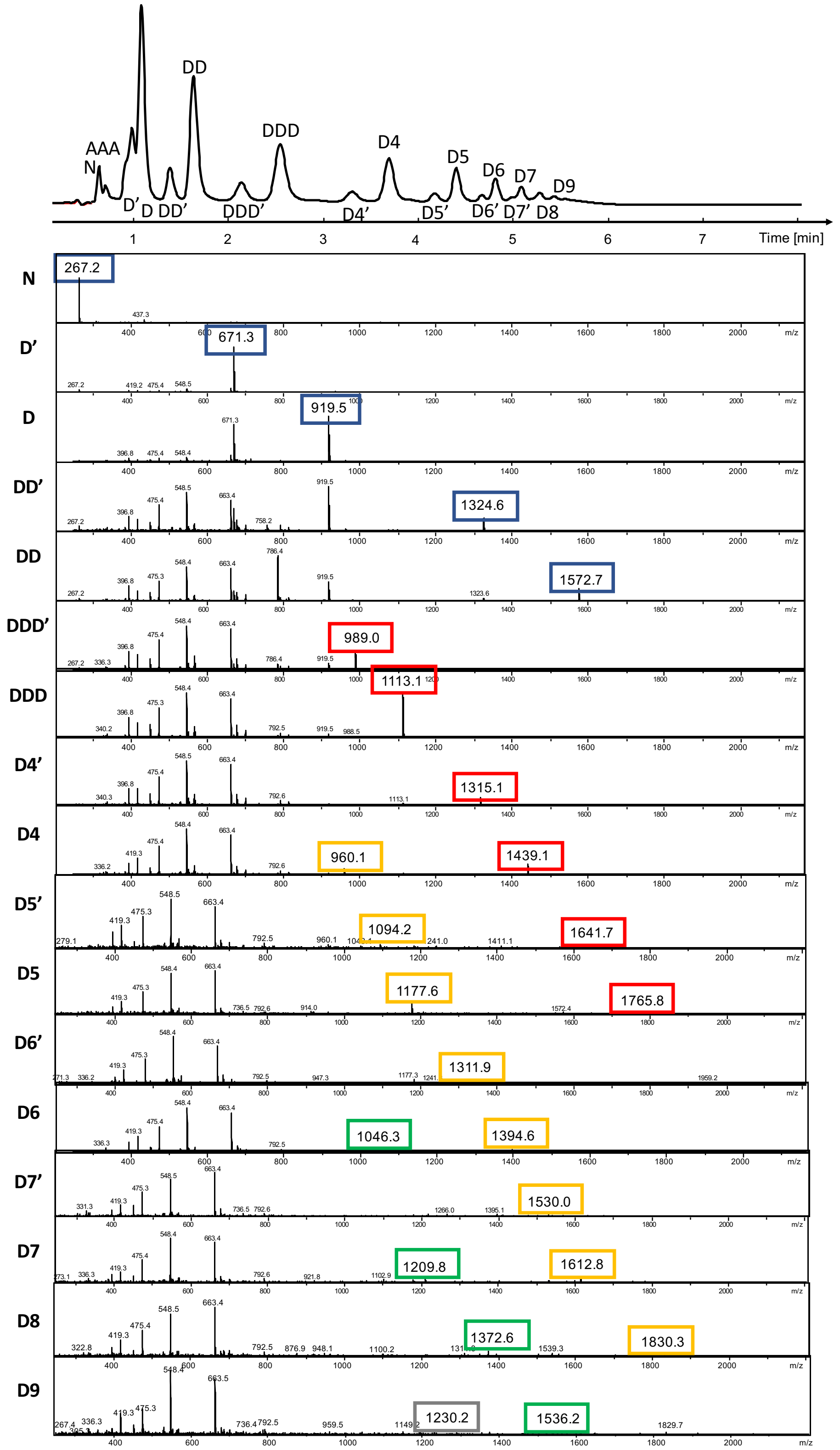

Figure S70. a) HPLC chromatogram (absorbance 284 –316 nm) of a mixture of **N** (20 mM) and **D** (10 mM) in toluene in the presence of 3.3 mM **AAA**, 15 hours after the addition of **N**. **b)** MS spectra of the monomeric and oligomeric components of the library. Characteristic mass values are highlighted with blue ($M+H^+$), red [$(M+2H^+)/2$], yellow [$(M+3H^+)/3$], green [$(M+4H^+)/4$ or in the case of **D9** $(M+3H^++Na^+)/4$] and grey [$(M+4H^++Na^+)/5$] squares. The dash in e.g. **D'** is used to indicate species with mono-imine mono-aldehyde terminal groups.

3.2 HPLC-MS Kinetic experiments: D-N

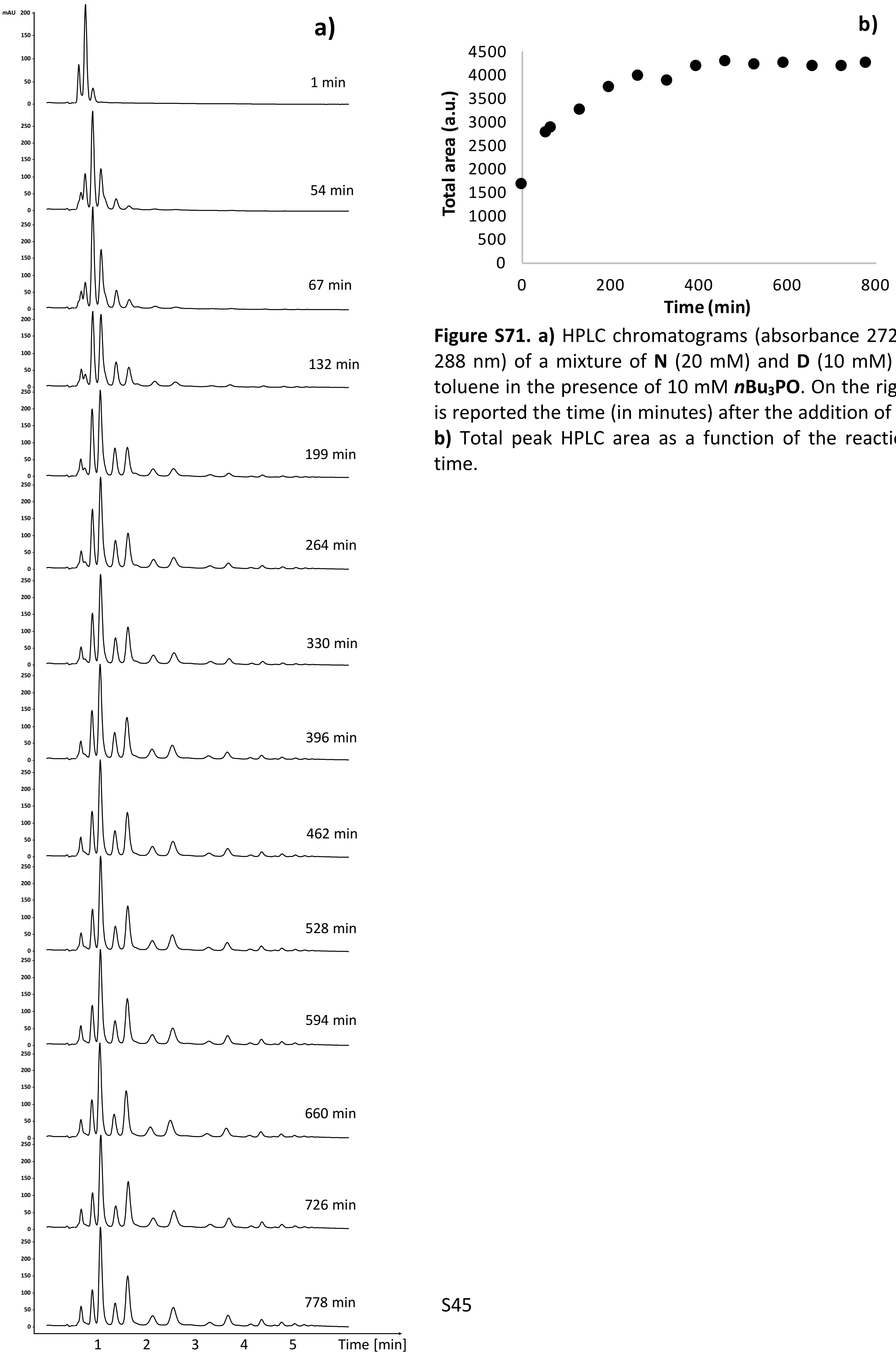

Figure S71. a) HPLC chromatograms (absorbance 272 – 288 nm) of a mixture of **N** (20 mM) and **D** (10 mM) in toluene in the presence of 10 mM ***n*Bu$_3$PO**. On the right is reported the time (in minutes) after the addition of **N**. **b)** Total peak HPLC area as a function of the reaction time.

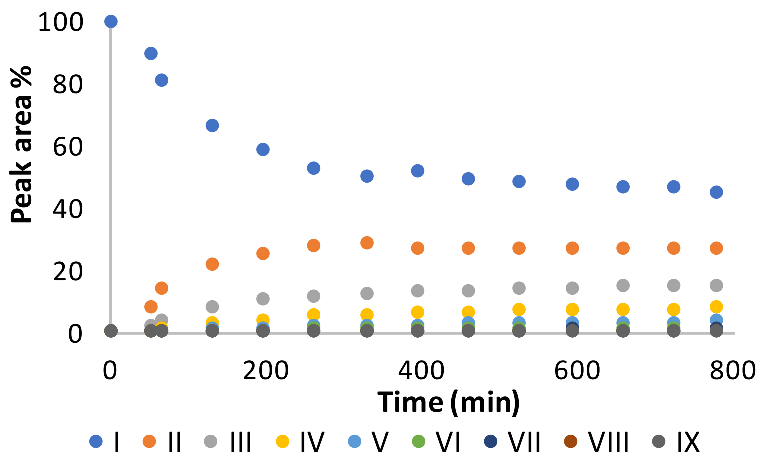

Figure S72. Kinetics of oligomers formation measured by integration of all the chromatogram peaks of a mixture of **N** (20 mM) and **D** (10 mM) in toluene in the presence of 10 mM ***n*Bu$_3$PO**. Every monomer or oligomer (identified with Roman numerals) is the sum of analogue species with imine and aldehyde terminal groups.

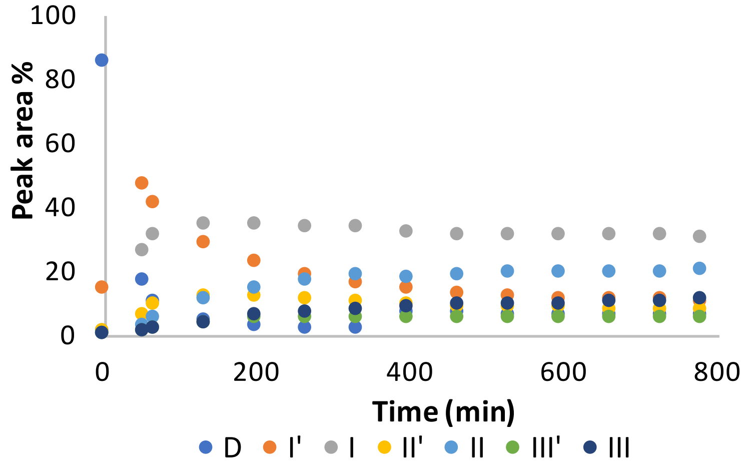

Figure S73. Kinetics of oligomers formation measured by integration of all the chromatogram peaks of a mixture of **N** (20 mM) and **D** (10 mM) in toluene in the presence of 10 mM ***n*Bu$_3$PO**. The main monomeric, dimeric and trimeric species having dialdehyde (**D**), mono-imine mono-aldehyde and diimine terminal groups are reported.

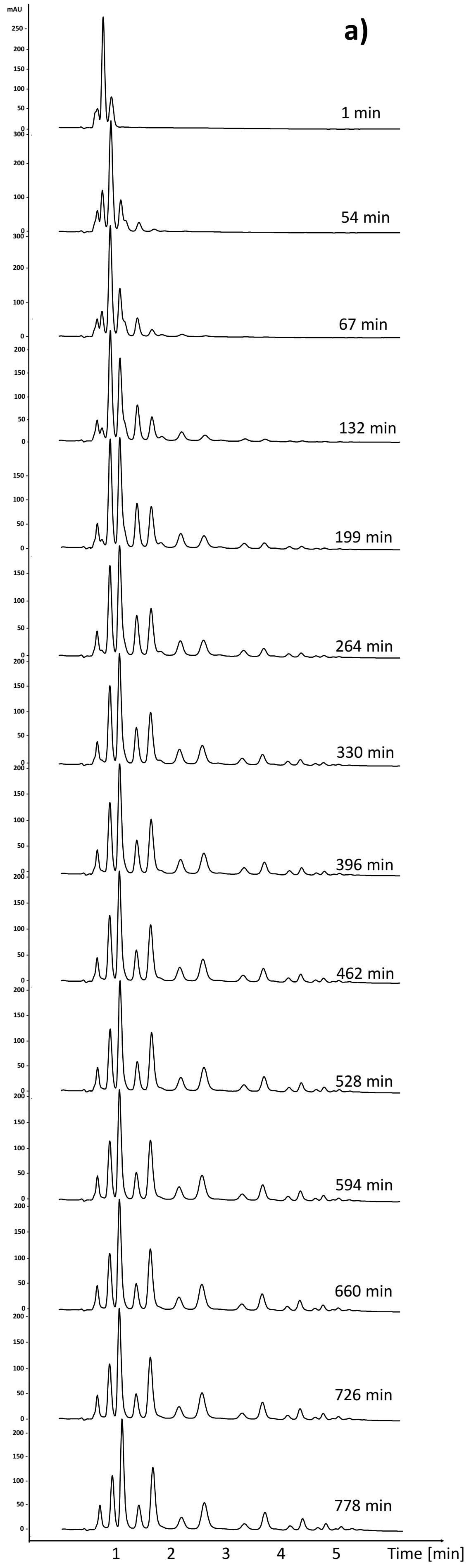

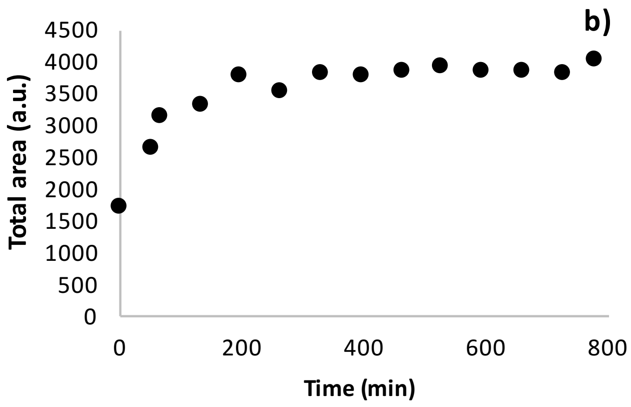

Figure S74. a) HPLC chromatograms (absorbance 272 – 288 nm) of a mixture of **N** (20 mM) and **D** (10 mM) in toluene in the presence of 5 mM **AA**. On the right is reported the time (in minutes) after the addition of **N**. **b)** Total HPLC peak area as a function of the reaction time.

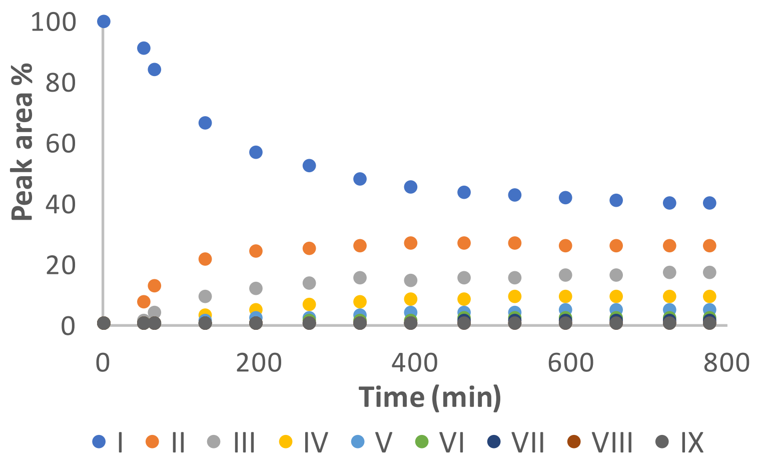

Figure S75. Kinetics of oligomers formation measured by integration of all the chromatogram peaks of a mixture of **N** (20 mM) and **D** (10 mM) in toluene in the presence of 5 mM **AA**. Every monomer or oligomer (identified with Roman numerals) is the sum of analogue species with imine and aldehyde terminal groups.

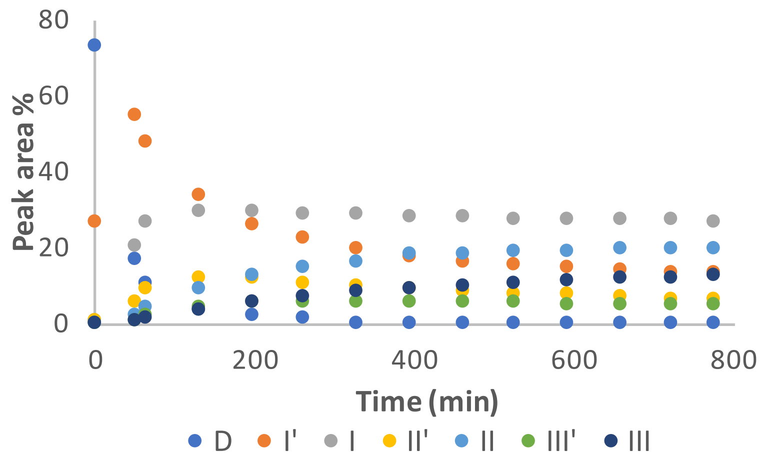

Figure S76. Kinetics of oligomers formation measured by integration of all the chromatogram peaks of a mixture of **N** (20 mM) and **D** (10 mM) in toluene in the presence of 5 mM **AA**. The main monomeric, dimeric and trimeric species having dialdehyde (**D**), mono-imine mono-aldehyde and diimine terminal groups are reported.

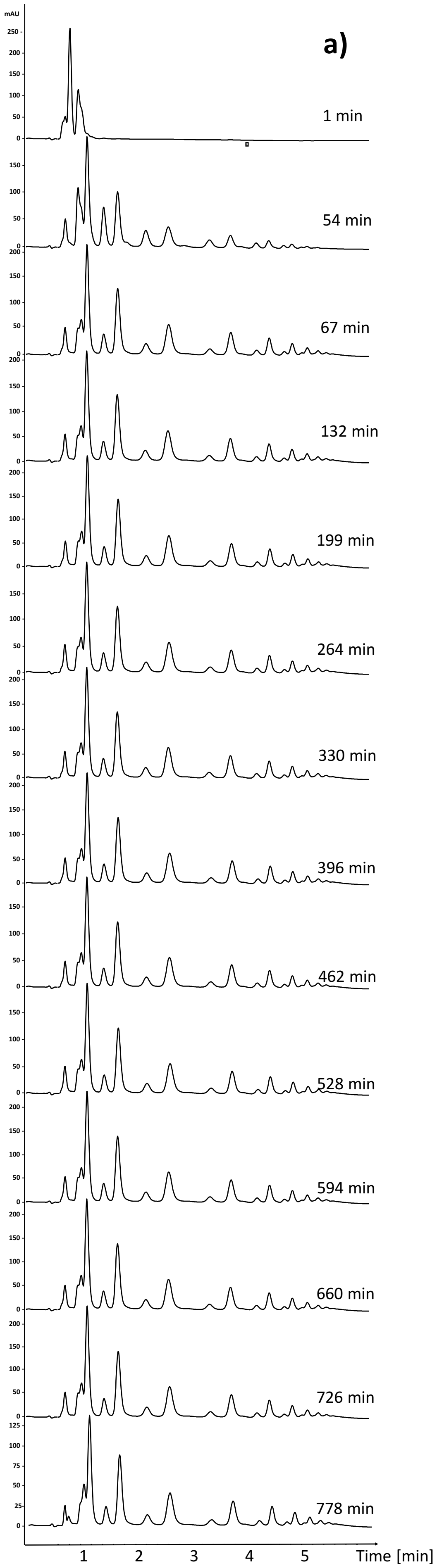

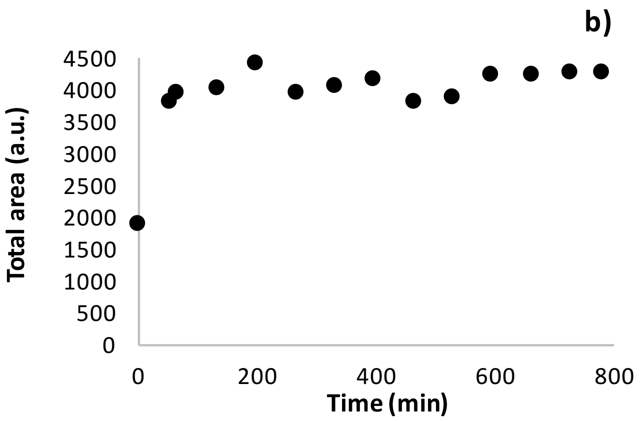

Figure S77. a) HPLC chromatograms (absorbance 272 – 288 nm) of a mixture of **N** (20 mM) and **D** (10 mM) in toluene in the presence of 3.3 mM **AAA**. On the right is reported the time (in minutes) after the addition of **N**. **b)** Total HPLC peak area as a function of reaction time.

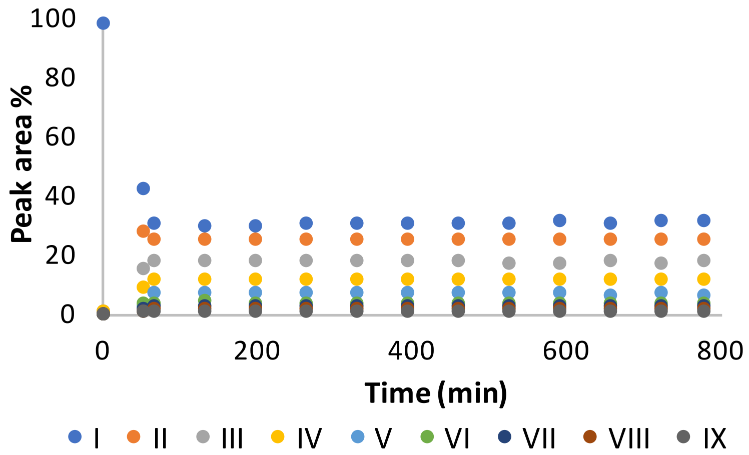

Figure S78. Kinetics of oligomers formation measured by integration of all the chromatogram peaks of a mixture of **N** (20 mM) and **D** (10 mM) in toluene in the presence of 3.3 mM **AAA**. Every monomer or oligomer (identified with Roman numerals) is the sum of analogue species with imine and aldehyde terminal groups.

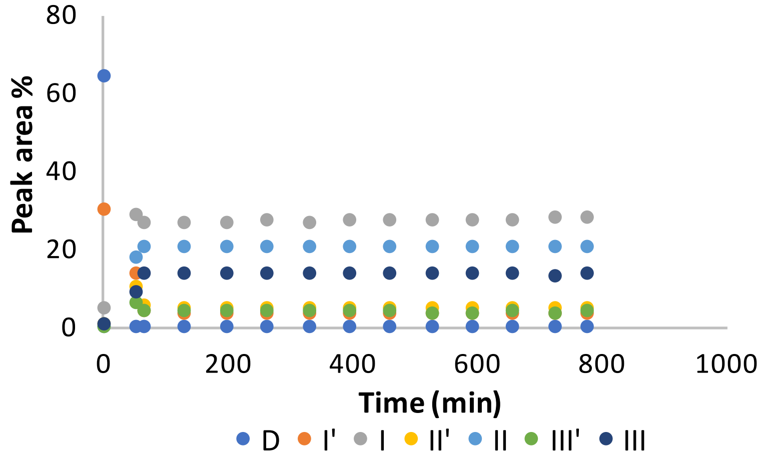

Figure S79. Kinetics of oligomers formation measured by integration of all the chromatogram peaks of a mixture of **N** (20 mM) and **D** (10 mM) in toluene in the presence of 3.3 mM **AAA**. The main monomeric, dimeric and trimeric species having dialdehyde (**D**), mono-imine mono-aldehyde and diimine terminal groups are reported.

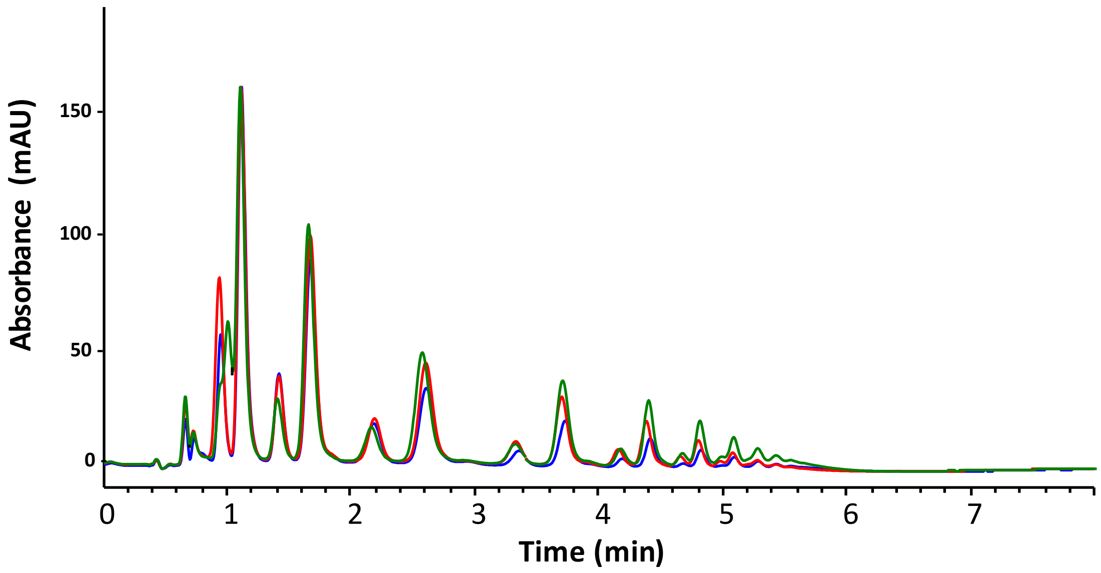

Figure S80. HPLC chromatograms (284-316 nm) of a toluene solution of donor **D** (10 mM) 15 hours after the addition of dianiline **N** (20 mM), in the presence of 10 mM ***n*Bu₃PO** (blue), 5 mM **AA** (red) or 3.3 mM **AAA** (green).

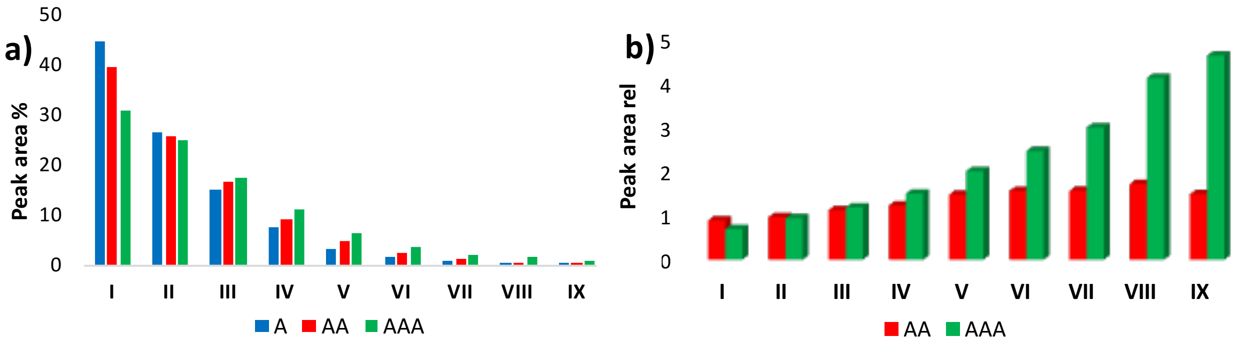

Figure S81. a) Library composition of a toluene solution of donor **D** (10 mM) 15 hours after the addition of dianiline **N** (20 mM), in the presence of 10 mM ***n*Bu₃PO** (blue), 5 mM **AA** (red) or 3.3 mM **AAA** (green). **b)** Peak area percentage for reactions in the presence of **AA** (5 mM, red) and **AAA** (3.3 mM, green), relative to the corresponding peak area percentage in the presence of ***n*Bu₃PO** (10 mM).

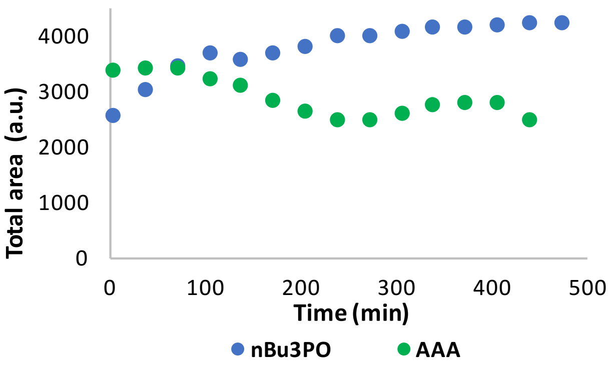

Figure S82. Total HPLC peak area as a function of the reaction time for a mixture of **N** (16 mM) and **D** (10 mM) in toluene, in the presence of ***n*Bu₃PO** (10 mM, blue circles) or **AAA** (3.3 mM, green circles). The decrease observed after 100 min for the **AAA** experiment is due to visible precipitation, presumably of longer oligomers (*cf* NMR experiments in Figure SS13).

4. References

1 L. Gabrielli, D Núñez-Villanueva, C. A. Hunter, *Chem. Sci.*, **2020**, *11*, 561.
